# Mechanically Programmable DNA Hydrogel Microparticles for 3D Cellular Systems

**DOI:** 10.1002/adma.202514218

**Published:** 2026-05-31

**Authors:** Tobias Walther, Eleni Dalaka, Gotthold Fläschner, Manuel Gómez‐González, Ilia Platzman, Sadaf Pashapour, Michelle Emmert, Pere Roca‐Cusachs, Xavier Trepat, Kerstin Göpfrich

**Affiliations:** ^1^ Center for Molecular Biology of Heidelberg University (ZMBH) Biophysical Engineering Group Heidelberg University Heidelberg Germany; ^2^ Institute for Bioengineering of Catalonia (IBEC) The Barcelona Institute of Science and Technology (BIST) Barcelona Spain; ^3^ Departament de Genètica Microbiologia i Estadística Facultat de Biologia Universitat de Barcelona Barcelona Spain; ^4^ Department of Cellular Biophysics Max Planck Institute for Medical Research Heidelberg Germany; ^5^ Institute for Molecular Systems Engineering and Advanced Materials (IMSEAM) Heidelberg University Heidelberg Germany; ^6^ Microfabrication and Microfluidics Core Facility (μFluCF) Institute for Molecular Systems Engineering and Advanced Materials (IMSEAM) Heidelberg University Heidelberg Germany; ^7^ Facultat de Medicina Universitat de Barcelona Barcelona Spain; ^8^ Institució Catalana de Recerca i Estudis Avançats (ICREA) Barcelona Spain; ^9^ Centro de Investigación Biomédica en Red en Bioingeniería Biomateriales y Nanomedicina (CIBER‐BBN) Barcelona Spain; ^10^ Cluster of Excellence SynthImmune Heidelberg University Heidelberg Germany

**Keywords:** 3D cell culture, biomaterials, DNA hydrogel, DNA nanotechnology, hydrogel microparticles, mechanobiology, microfluidics

## Abstract

Hydrogel microparticles (HMPs) are powerful tools to study and manipulate cellular behavior in 3D cell culture systems and animal models. Here, fully DNA‐based HMPs are presented, whose material properties can be precisely tuned by sequence‐programmable design of self‐assembling DNA nanostructures. These DNA‐HMPs offer control over size, stiffness, viscoelasticity and ligand presentation. They are formed by microfluidic encapsulation of two types of orthogonal DNA nanostars and a sequence‐complementary DNA linker in water‐in‐oil droplets. By varying the valency of the DNA nanostar designs, tunable mechanical properties are achieved – spanning three orders of magnitude in Young's modulus from 30Pa to 6.5kPa with distinct viscoelastic behavior. Click‐chemistry based functionalization with the small fibronectin‐derived peptide cyclic‐RGD (c[RGD]) enables integration into fibroblast spheroids. DNA‐HMPs are stably retained within the spheroids for several days and undergo remodeling, indicating active interactions between the cells and the DNA‐HMPs. Combining programmable material properties and inherent biocompatibility of DNA with straightforward functionalization and stimuli‐responsiveness, these DNA‐HMPs represent a versatile tool to probe and manipulate tissue behaviors in 3D cell cultures.

## Graphical abstract

DNA hydrogel microparticles are designed to exhibit controllable viscoelasticity and stiffness across three orders of magnitude from 30Pa to 6.5kPa. They are uptaken into fibroblast spheroids where they are actively remodeled by cellular forces depending on their mechanical properties.

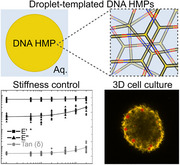

## 1 Introduction

Tools that enable localized perturbation and monitoring of the cell microenvironment are essential to advance 3D cell culture for tissue engineering, disease modeling, and precision medicine [1, 2]. Hydrogel microparticles (HMPs) – micron‐sized gel beads – are well‐suited for this task. Unlike conventional bulk hydrogels, they are cell‐sized objects that allow biochemical and mechanical cues to be applied from within complex multicellular systems, as well as enabling real‐time readouts [3, 4, 5, 6]. HMPs have been produced from polymers such as alginate [7, 8, 9, 10], polyacrylamide [11], polyethylene glycol [12], and agarose [13, 14]. They are prominently used as cell culture substrates [15, 16] or as adjuvants that provide physical or chemical signaling [6, 17, 18]. Beyond this, they serve as spatially discrete sensors in tissues owing to their elastic material properties [11, 19, 20, 21, 22]. However, most conventional polymeric HMPs lack versatility in terms of the capacity for targeted chemical functionalization, fine‐scale mechanical programmability, and integration of dynamic or stimuli‐responsive behavior, such as controlled structural changes or molecular release in response to environmental cues. DNA‐based materials offer a promising alternative due to their sequence‐defined self‐assembly, precise mechanical tuning, and straightforward chemical modification [23, 24, 25]. DNA hydrogels have been applied in various biomedical contexts [26, 27, 28, 29], yet efforts have focused on bulk gels. Despite extensive rheological studies of bulk DNA networks showing storage moduli ranging from 10Pa to 100kPa [30, 31, 32, 33, 34, 35], HMPs made from DNA hydrogels with controllable size, mechanical properties, and biofunctionality have not yet been realized. Moreover, such materials have not been explored as deformable force‐responsive elements within living tissues. Here, we thus introduce fully DNA‐based hydrogel microparticles (DNA‐HMPs) with programmable viscoelastic properties and functional biointerfaces. These particles are formed by microfluidic co‐encapsulation of self‐assembling DNA nanostars and sequence‐complementary linkers. They exhibit finely tunable Young's moduli over three orders of magnitude from 30Pa to 6.5kPa that offer exceptional resolution in material design. Beyond microfluidics, DNA‐HMPs can also be generated by simple vortexing of the solution making their production broadly accessible without specialized equipment. Once formed, the DNA‐HMPs are highly stable and retain their structure for at least 1 year under refrigerated conditions. They behave predominantly elastic under low‐frequency indentation, but their viscoelastic response can be modulated through DNA nanostar and linker design. The HMPs can be further functionalized via click chemistry with cyclic‐RGD (c[RGD])‐peptides to enable active cellular interaction. When added to fibroblast spheroids, the DNA‐HMPs are readily internalized, stably retained, and undergo deformation and remodeling based on compressive forces inside the spheroids. Combining modular mechanical programming, biochemical customization, and biocompatibility, DNA‐HMPs establish a new class of materials to interact with the 3D cell microenvironment. In the future, DNA‐HMPs can therefore serve as a platform for tissue engineering approaches and biophysical analysis in a variety of setups.

## 2 Results and Discussion

### 2.1 Size‐Controlled Formation of DNA‐HMPs by Microfluidics

**FIGURE 1:**
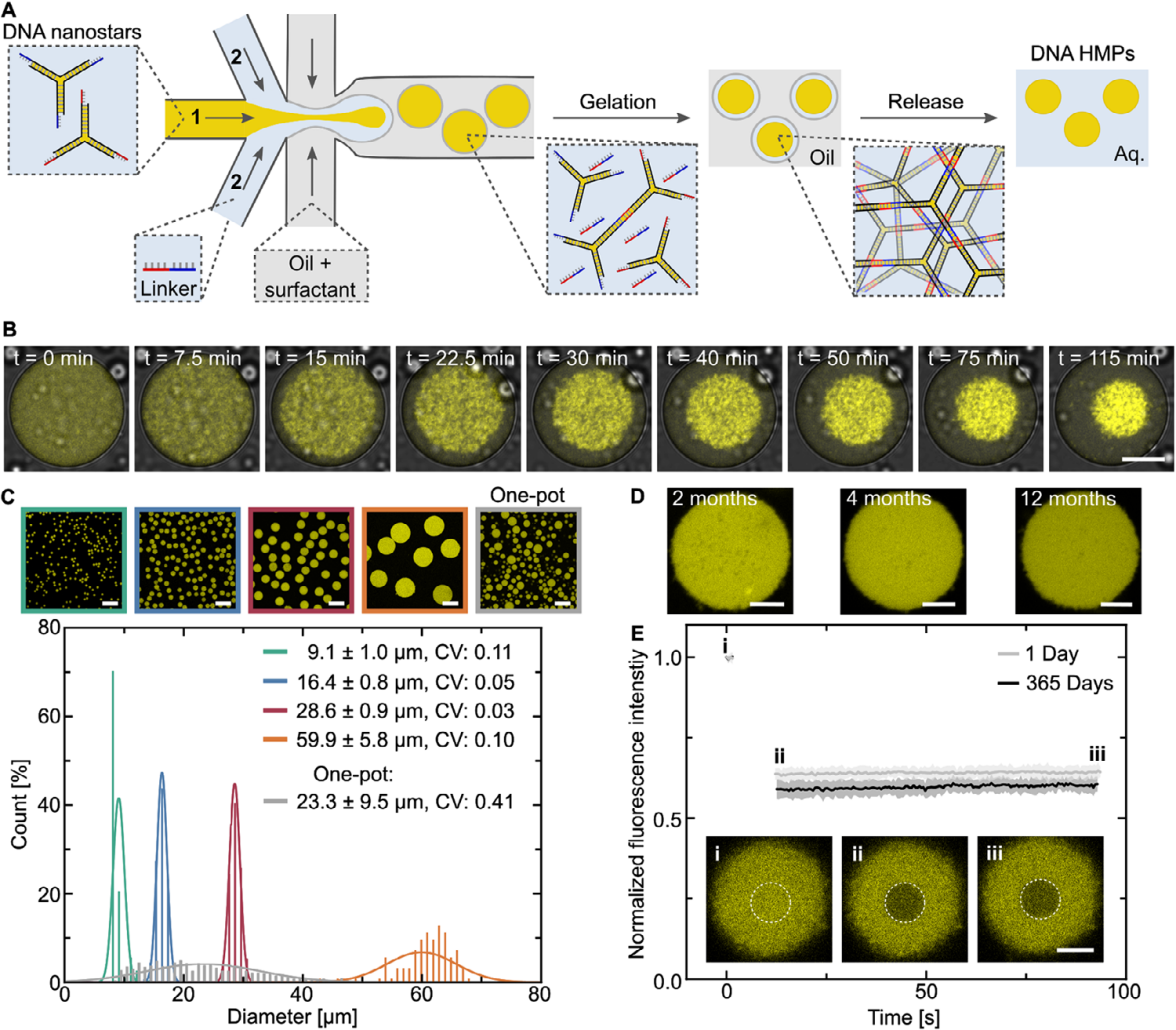
Microfluidic formation of droplet‐templated DNA‐HMPs. (A) Schematic of the procedure for DNA‐HMP formation. Two DNA nanostars (A and B) with non‐complementary overhangs are encapsulated with a DNA linker using a double‐inlet microfluidic device. Upon encapsulation, DNA hydrogel formation is initiated. After full gelation, DNA‐HMPs are released from their droplet shell into an aqueous solution. (B) Overlay of confocal microscopy ($\lambda_{ex}=561$ nm, Cy3‐labeled DNA, yellow) and brightfield images of a DNA‐HMP forming inside a water‐in‐oil droplet over the course of 115min, showing how the DNA condenses to form a single DNA‐HMP per droplet. Scale bar: 20μm. (C) Confocal microscopy ($\lambda_{ex}=561$ nm, Cy3‐labeled DNA, yellow) images of DNA‐HMPs prepared at different monodisperse sizes by controlling the flow rates of the aqueous and oil phases in the microfluidic device. Less uniform DNA‐HMPs formed via the one‐pot method are shown for comparison (gray distribution). Scale bars: 50μm. Histograms of the size distributions of the DNA‐HMPs with Gaussian fits. n_green_ = 268, n_blue_ = 432, n_red_ = 205, n_orange_ = 125, n_gray_ = 1438. Mean and standard deviation correspond to the mean and standard deviation of the Gaussian fit of each distribution. (D) Long‐term stability of DNA‐HMPs. Confocal fluorescence microscopy images after 2, 4, and 12 months storage at 4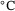. Scale bars: 10μm. (E) FRAP of DNA‐HMPs (mean ± standard deviation, n = 3) on the day of release (1 Day) and after a 12 months storage period. Inserted confocal micrographs before (*i*) and after bleaching (*ii*, *iii*) after 12 months of storage. Scale bar: 10μm.

Toward applications in 3D cell culture, we first derived a strategy to produce DNA‐HMPs in a reproducible and size‐controlled manner. We used microfluidics to encapsulate the hydrogel‐forming DNA components into water‐in‐oil droplets. After gel formation, stable DNA‐HMPs were released from the droplet shell into an aqueous environment as illustrated in Figure 1A. We selected a DNA nanostar design which has been shown to form stable hydrogels in bulk [36, 37]. The design is composed of three single strands of DNA, which form a 3‐arm nanostructure upon thermal annealing (for details see Experimental Section). To enable imaging of the resulting DNA‐HMPs by fluorescence microscopy, one of the single strands was covalently labeled with a Cyanine‐3 (Cy3) dye (for details see Experimental Section). Two sets of such DNA nanostars (A and B), each equipped with orthogonal nine nucleotide‐long overhangs on all three arms, form the monomeric units of the hydrogel. As A and B have non‐complementary overhangs, cross‐linking and hydrogel formation is only initiated upon addition of an 18 nucleotide long single‐stranded DNA linker (for DNA sequences see Table S1) [36, 37]. In the following, we will refer to this design as “3‐arm short” to distinguish it from other designs that will be introduced later.

To prevent clogging of the microfluidic device by gel‐forming DNA species, we designed a double‐inlet microfluidic device where A and B only encounter the DNA linker after the formation of the water‐in‐oil droplet (for chip layout see Figure S1). To form the DNA‐HMPs, A and B were supplied in one inlet, while the second inlet delivered the DNA linker, added at three times molar excess to the 3‐arm short DNA nanostars, such that each of the three arms binds one linker. At the intersection of the aqueous phases with a surfactant‐oil phase, water‐in‐oil droplets formed, encapsulating the DNA components.

Sequence‐specific binding of the DNA linker to both types of sticky‐end overhangs inside the water‐in‐oil droplets then resulted in the formation of spherical DNA hydrogels which condensed into stable DNA‐HMPs over the course of several hours (Figure 1B). Note that for maximum yield, DNA‐HMPs were normally matured for 72 h (Figure S2). The DNA‐HMPs were then released from their droplet shells by adding a droplet‐destabilizing surfactant, perfluoro‐1‐octanol, as previously used for the formation and release of droplet‐stabilized giant unilamellar vesicles [38]. This allowed us to collect the fully‐formed, stable DNA‐HMPs in aqueous solution (Figure 1A). Control experiments confirmed that formation of the DNA‐HMPs only occurred when both the nanostars and the DNA linker were co‐encapsulated. DNA‐HMP formation critically depends on the linker concentration, whereby the optimal concentration can be derived from the valency of the DNA nanostar (Figure S3).

Tracking the formation of the DNA‐HMPs in water‐in‐oil droplets over time, we observe the formation of smaller DNA clusters that then condense into a singular spherical DNA‐HMP per droplet (Figure S4A, Video S1). During this process, the area fraction of the fluorescent signal inside the droplets and thus the size of the forming DNA‐HMP decreases over the course of 2 h before reaching a plateau, indicating maturation of the DNA‐HMP (Figure 1B, Figure S4B–D). Cryogenic scanning electron microscopy (cryoSEM) further revealed that the DNA‐HMPs consist of interconnected domains of higher DNA density with a diameter on the order of 200nm (Figure S4E/F). This architecture indicates that the condensation of the DNA into HMPs is aided by liquid‐liquid phase separation on the nanoscale [39]. Lastly, we conducted fluorescence recovery after photobleaching (FRAP) experiments on the covalently‐attached fluorophores using different bleaching areas, laser intensities, temperatures and dye concentrations to understand network connectivity. We find no signal recovery for any of the tested conditions (Figure S5), denoting very low fluidity of the DNA network. Hence, we do not observe translational motion of connected DNA nanostars inside the particle network. Control bleaching experiments on fluorophores which were not covalently bound to the DNA, such as DNA‐intercalating Hoechst dyes and fluorescently‐labeled DNA nanostars with orthogonal sticky‐end overhangs, however, showed clear signal recovery within the observed time‐frame (Figure S6). We therefore conclude that the DNA‐HMPs depict a “gel‐like state” within the conditions presented here in agreement with previous work in the field [40, 41].

In order to create uniform DNA‐HMPs of varying sizes, we adjusted the flow‐rates of the oil‐ and the aqueous phase, respectively, fine‐tuning the diameter of the resulting droplets. We formed DNA‐HMPs with narrow size distributions of 9.1μm ± 1.0μm, 16.4μm ± 0.8μm and 28.6μm ± 0.9μm (Figure 1C, for corresponding flow rates see Experimental Section). Utilizing a wider microfluidic channel of 60μm instead of 30μm, we were able to produce DNA‐HMPs as large as 59.9μm ± 5.8μm (Figure 1C), allowing us to mimic a vast range of cell sizes found in mammalian tissues [42]. For the production of large quantities of DNA‐HMPs, we additionally developed a scalable one‐pot method. It involves layering the DNA‐containing solution on top of the oil surfactant mix in a reaction tube or any larger reaction container. Instead of using microfluidics, the formation of water‐in‐oil droplets is induced by vortexing. While the one‐pot method results in a broader size distribution (23.3μm ± 9.5μm, Figure 1C), it is fast, scalable and does not require specialized expertise or equipment. It thus facilitates the implementation of DNA‐HMPs in laboratories without access to microfluidics when stringent size control is not required. Analyzing the particle aspect ratio of the DNA‐HMPs, we confirm formation of intact spherical particles for both methods (Figure S7).

DNA‐HMPs can be stored in solution at $4^{\circ }C$ for at least 1 year without any apparent morphological changes, loss of structural integrity or changes in particle size (Figure 1D, Figure S8), making their handling straightforward. FRAP experiments on 1 year old DNA‐HMPs show no recovery and hence confirm network stability (Figure 1E, Video S2). Additionally, DNA‐HMPs settle inside a reaction tube within minutes and can be pelleted by centrifugation using a simple table‐top spinner. This allows for quick buffer exchange, washing and functionalization which is key for downstream applications.

### 2.2 Characterization of the Material Properties of DNA‐HMPs Programmable by Sequence Design

**FIGURE 2:**
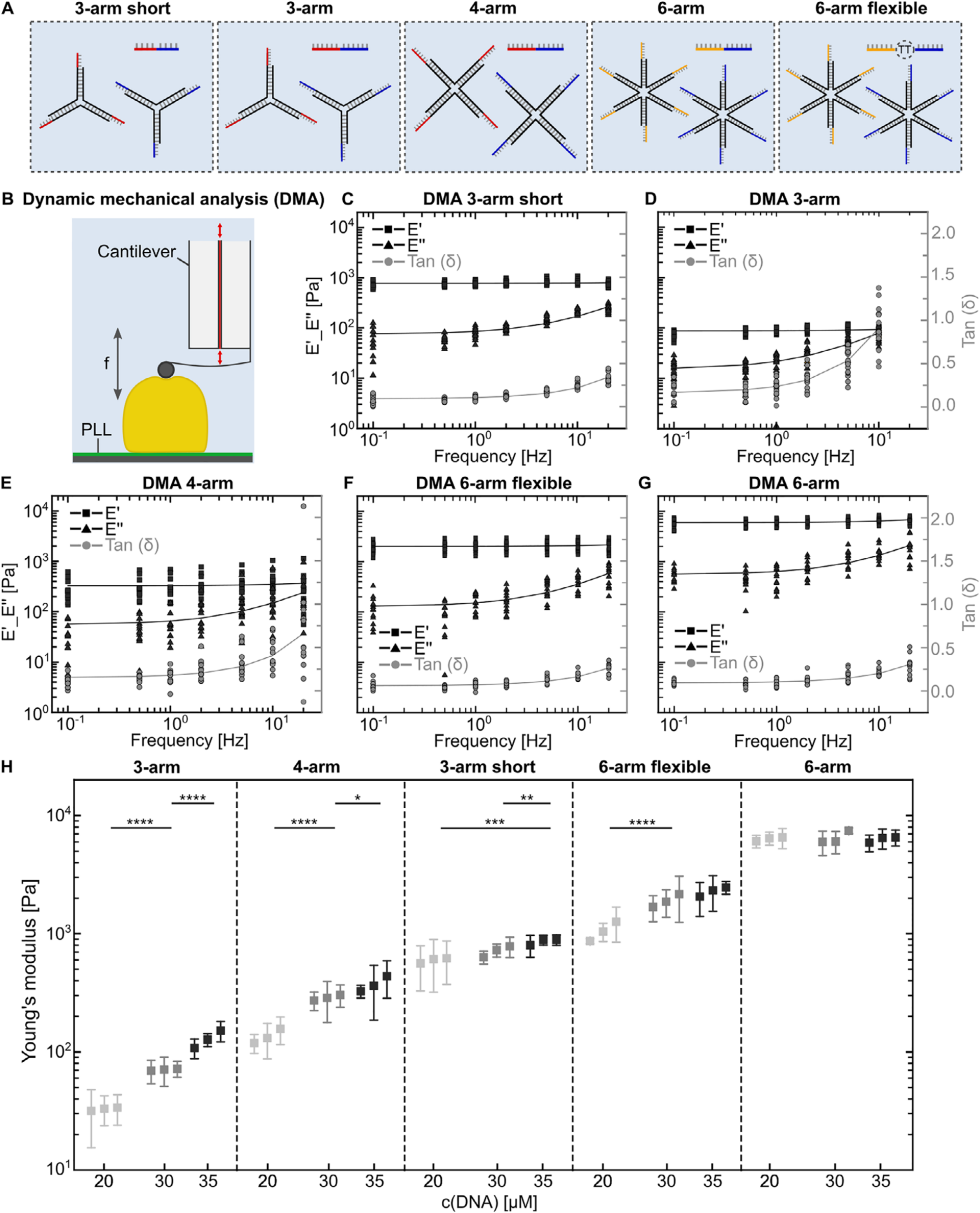
Influence of DNA nanostar sequence design on the material properties of DNA‐HMPs. (A) Schematic representation of the five different DNA nanostar designs with their respective linkers used to form DNA‐HMPs. (B) Schematic of HMP indentation using dynamic mechanical analysis (DMA). DNA‐HMPs are adhered to a glass substrate by electrostatic interaction with PLL and indented with increasing frequency *f* using a cantilever. (C–G) DMA of 3‐arm short (C), 3‐arm (D), 4‐arm (E), 6‐arm flexible (F) and 6‐arm (G) DNA‐HMPs at 35 μM DNA nanostar concentration. The storage modulus E' (squares) and the loss modulus E″ (triangles) are plotted on a logarithmic scale, tan (δ) (E″/E', filled circles) is plotted on a linear scale. H) Full analysis of Young's moduli of DNA‐HMPs across different designs and DNA concentrations as extracted from Hertz‐model fits of the indentation curves during microindentation. The Young's moduli for 3‐arm, 4‐arm, 3‐arm short, 6‐arm flexible and 6‐arm DNA‐HMPs at DNA monomer concentrations of 20, 30, and 35 μM (from left to right) are displayed. The data is presented as mean ± standard deviation of each measurement for n = 3 independent experiments per condition measuring at least 3 particles each. 3‐arm ****p‐values: 9.9 × 10^−8^, 3.3 × 10^−11^. 4‐arm *p‐value: 0.039, ****p‐value: 8 × 10^−8^. 3‐arm short **p‐value: 0.003, ***p‐value: 0.0008. 6‐arm flexible ****p‐value: 0.0001. Statistical significance was assessed using unpaired Student's *t*‐tests.

Having demonstrated the feasibility of DNA‐HMPs, we next explored whether their network connectivity can be tuned by altering the valency of the DNA nanostars [25, 28, 41, 43] and their arm lengths. Thus, we designed three more DNA nanostars, named 3‐arm, 4‐arm and 6‐arm (Figure 2A). In the updated 3‐arm design, the sticky‐end overhangs of the 3‐arm short DNA nanostar (Figure 2A) were elongated by three additional nucleotides. The DNA linker was extended accordingly, resulting in an increase of the melting temperature from $46^{\circ }C$ (unbound fraction at $37^{\circ }C$ = 23%) to $66^{\circ }C$ (unbound fraction at $37^{\circ }C$ = 8.8%). We reasoned that this higher thermal stability would improve the suitability of the DNA‐HMPs for applications in cell culture at physiological temperature. Hence, we used the elongated linker also for the higher‐valency nanostars with 4 and 6 arms (Figure 2A, for DNA sequences see Table S1). Linker melting curves, ΔG analysis and binding behavior of all structures were analyzed and verified using NuPack [44] (Figures S9/S10).

While DNA‐HMPs from the 3‐arm and 4‐arm DNA nanostars were readily formed at room temperature (Figure S11A/B), 6‐arm DNA‐HMPs required thermal annealing. The need for annealing of the 6‐arm DNA‐HMPs is most likely due to their valency. As the number of junction points increases, so does the connectivity within the network until a critical value, i.e. isostatic point, is reached after which networks drastically increase in rigidity and strain. For DNA nanostar networks, this isostatic point is expected around a valency of 6 [45]. DNA nanostar networks of lower valency are thus more flexible and allow for easier network perturbations and rearrangements than higher valency structures. We observe behavior consistent with this for the 6‐arm design, as non‐annealed DNA‐HMPs show irregular and brittle behavior (Figure S12A). Thermal annealing ensures binding in a more controlled way and enables the formation of stable 6‐arm DNA‐HMPs beyond the isostatic point (Figures S11C/S12A, for details see Experimental Section).

To overcome the need for annealing, we reasoned that introducing flexibility in the DNA linkers would also enhance the chances of complete binding of the network [46, 47]. We thus added two flexible thymine‐nucleotides to the center of the 6‐arm linker (6‐arm flexible), which rescued 6‐arm DNA‐HMP formation at room temperature (Table S1, Figures 2A, S11D/S12B). We further confirmed hybridization stability within the DNA‐HMP designs based on heating analysis showing good agreement with DNA linker melting temperatures and higher network connectivity for the 6‐arm designs (Figure S13). FRAP analysis on the 3‐arm, 4‐arm, 6‐arm flexible and 6‐arm DNA‐HMP finally also show intact “gel‐like state” for all designed HMPs (Figures S14‐S17).

In summary, we developed five distinct DNA nanostar designs that allow for the controlled formation of DNA‐HMPs inside water‐in‐oil droplets (Figure S18).

Based on these designs, we then set out to investigate how nanostar valency and design influence the material properties of DNA‐HMPs. Both the stiffness and viscoelasticity of cells and tissues play critical roles in regulating cellular functions [48]. These mechanical properties should therefore be carefully considered when designing materials for tissue engineering applications [49, 50]. Cells exhibit type‐specific Young's moduli ranging from around 0.1kPa in neurons to 20kPa and above for osteoblasts [49, 51]. Similarly, the environment and tissues surrounding cells vary greatly from less than 0.1kPa in mucus up to the GPa range in bone [49, 52]. In addition, cells and their microenvironment also display a broad range of viscoelastic properties [48, 53]. To analyze the different designs and determine their material properties, we turned to microindentation measurements by dynamic mechanical analysis (DMA) as illustrated in Figure 2B. In DMA, a material is exposed to sinusoidal stress as a function of outer parameters such as frequency. Hence, DMA gives insights into the responsiveness of a given material to repeated physical change, allowing for the determination of material properties [54]. For this, the DNA‐HMPs were adhered to a glass substrate coated with positively‐charged poly‐l‐lysine (PLL, MW = 150 –300 kDa) by electrostatic interaction (Figure S19), as unbound HMPs did not allow for a stringent experimental setup. Since substrate adhesion can affect measurement results during DMA, indentation events were closely controlled during measurements (for details see Experimental Section).

3‐arm short DNA‐HMPs exhibited frequency‐independent storage moduli (E') of 607Pa ± 287Pa at 20 μM nanostar concentration to 885Pa ± 88Pa at 35 μM nanostar concentration (Figure 2C, Figure S20). In the low frequency range (<1 Hz) the loss moduli (E″) of the DNA‐HMPs were substantially lower and likewise frequency‐independent. A slight increase of E″ was found at higher frequencies (>5 Hz), but never exceeding E'. This is also reflected in the loss tangent tan(δ), which even at the highest frequency tested (20 Hz) remains <0.5. These data indicate that the 3‐arm short HMPs behave largely as elastic materials, displaying viscous dissipation only at higher frequencies. These findings are in line with previous work on the rheological behavior of comparable macroscale bulk DNA hydrogels [32, 47, 55, 56].

We hypothesized that elongating DNA nanostar arm lengths would result in a more porous and softer network, owing to the formation of larger nanogel clusters taking up a greater volume fraction of the resulting hydrogel [57, 58]. Conversely, increasing nanostar valency was expected to yield stiffer DNA‐HMPs due to higher connectivity within the DNA network [58].

In agreement with these expectations, DMA revealed that the 3‐arm DNA‐HMPs are softer and more viscous than those formed with the 3‐arm short design (Figure 2D, Figure S21). HMPs assembled from the 4‐arm design displayed similar viscoelastic behavior at monomer concentrations below 35 μM (Figure S22), while those formed at 35 μM showed increased elasticity (Figure 2E). These results indicate that the viscoelastic properties of our DNA‐HMPs can be programmed by nanoscale design. Networks with lower connectivity exhibit more viscous behavior, whereas networks with higher valency and DNA concentration are more elastic. DMA of the 6‐arm flexible and 6‐arm HMPs further underline this trend. Across all tested concentrations and frequencies both types of DNA‐HMPs showed predominantly elastic behavior with storage moduli reaching up to 2.4kPa ± 303Pa for the 6‐arm flexible HMPs and 6.5kPa ± 1kPa for the 6‐arm HMPs (Figure 2F/G, Figures S23/S24). Comparing these findings with literature, we find that they are well in line with reports showing lower elastic moduli in macroscale bulk DNA hydrogels with increased flexibility [32, 46, 47, 59]. Consistent with DMA analysis, the relaxation behavior of the DNA‐HMPs following indentation showed a more viscous response for the 3‐arm and 4‐arm designs compared to the other HMPs (Figure S25). Indeed, 3‐arm and 4‐arm HMPs showed stronger decrease in initial load prior to relaxation, indicating higher viscosity, while both types of 6‐arm HMPs exhibited less load decrease and thus a more elastic response (Figure S25). Finally, increasing the DNA monomer concentration also increased DNA‐HMP stiffness, albeit to a lesser extent than sequence design (Figure 2H). Taken together, our findings indicate that DNA‐HMPs function as microscale DNA hydrogels whose mechanical properties can be precisely modulated within the cell stiffness and viscoelasticity range, exhibiting cell‐like loss tangent behavior [60], by adjusting nanostructural design.

### 2.3 Real‐Time Deformability Cytometry of DNA‐HMPs

To examine the material properties of DNA‐HMPs at high‐frequency manipulation and to verify their Young's moduli with an independent measurement, we subjected our DNA‐HMPs to real‐time deformability cytometry (RT‐DC); a microfluidic method used to measure cell stiffness in a contact‐free and high‐throughput manner [61]. Cells are flushed through a narrow channel such that they deform under hydrodynamic shear force. From the deformation and flow speed, it is possible to infer Young's moduli on the order of 0.1kPa to around 5kPa of thousands of cells within minutes based on mapping particle deformation to numerical simulations [13, 61, 62, 63]. RT‐DC is highly complementary to indentation based methods as it offers contact‐free measurements in solution at much higher throughput – we obtain data on tens of thousands of HMPs per experiment instead of tens measured by indentation. As RT‐DC measurements are conducted in a contact‐free manner, we can further cross‐validate the stringency of our DMA measurements.

**FIGURE 3:**
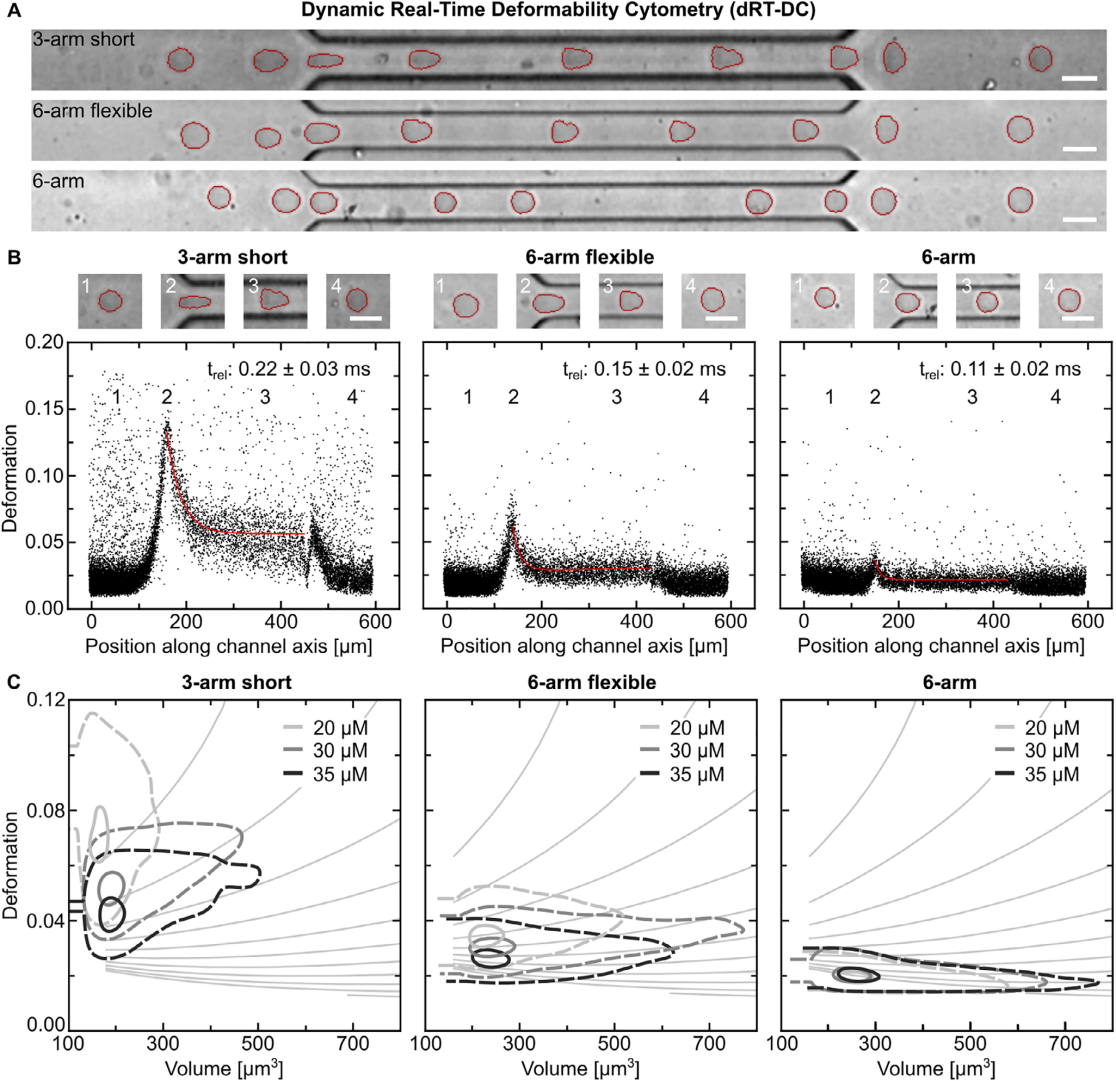
Analysis of the material properties of DNA‐HMPs using real‐time deformability cytometry (RT‐DC). (A) Composite images of DNA‐HMPs (3‐arm short, 6‐arm flexible and 6‐arm) inside the flow channel during dynamic RT‐DC (dRT‐DC). The red lines indicate the detected contours of the HMPs. DNA‐HMPs are initially spherical and deform upon entering the channel. Within milliseconds, they reach a steady‐state deformation. After leaving the channel, they return to their spherical shape. Scale bar: 20μm. (B) Deformation scatter plots of 35 μM 3‐arm short, 6‐arm flexible and 6‐arm HMPs plotted over the channel length during dRT‐DC. Each point corresponds to an individual DNA‐HMP (n = 13000). Insets 1–4 show DNA‐HMPs at the corresponding positions during dRT‐DC depicted in the scatter plots. The relaxation time t_rel_ of the DNA‐HMPs was extracted from the exponential fit of the relaxation scatter plot (red curve). For calculation of the flow time see Experimental Section. Scale bars: 20μm. (C) Exemplary contour plots showing DNA‐HMP deformation over the particle volume for different DNA nanostar concentrations for the 3‐arm short, 6‐arm flexible and 6‐arm designs. The data is presented using contour plots showing the 50th percentile (dashed line) and 95th percentile (solid line). Isoelasticity lines derived from numerical simulations are shown additionally, indicating stiffness changes where a steeper slope corresponds to softer particles. Only one repeat per condition is depicted to improve readability. For the full set of replicates see Figure S26.

The DNA‐HMPs deformed upon entering the RT‐DC channel, relaxing into a steady‐state deformation once fully inside the channel. After leaving the channel, they returned to their initial spherical shape, indicating the absence of plastic deformation under the applied shear force (Figure 3A). The images presented in Figure 3A were acquired by increasing the size of the imaged channel region during RT‐DC to span the entire length of the channel and beyond, while increasing the imaging speed to 7000 f/s, tracking HMP deformation in a quasi‐dynamic RT‐DC (dRT‐DC) experiment [63]. Given the low optical contrast of our HMPs, we adjusted the RT‐DC workflow to include a 20× phase‐contrast objective (see Experimental Section). Comparison of the deformation during RT‐DC for the 3‐arm short, 6‐arm flexible, and 6‐arm DNA‐HMPs reveals clear design‐dependent differences in deformability. The 3‐arm short HMPs show the highest deformation adopting a bullet shape as they transverse the channel, while deformation is less pronounced for the 6‐arm flexible design and nearly absent for 6‐arm HMPs (deformation < 0.03, Figure 3A/B).

As shown in Figure 3B, DNA‐HMP deformation peaked for all three designs just after entering the channel and rapidly decreased during relaxation into a steady‐state followed by a return to a spherical shape upon leaving the channel. The differences between the three designs can be seen from the marked decrease in initial deformation from 0.15 for the 3‐arm short HMPs to 0.075 for the 6‐arm flexible design and 0.03 for the 6‐arm HMPs, which thus remained almost spherical inside the channel, exhibiting a weak bullet shape. Exponential fits to the slope of DNA‐HMP relaxation following initial deformation revealed characteristic response times τ of 0.22 ± 0.03 ms (3‐arm short), 0.15 ± 0.02 ms (6‐arm flexible) and 0.11 ± 0.02 ms (6‐arm) respectively (Figure 3B), showing faster relaxation responses for higher valency, stiffer DNA‐HMPs.

Plotting DNA‐HMP deformation against their volume, we show that an increase in DNA nanostar concentration also resulted in a decrease in deformability, in line with our results obtained by DMA (Figure 3C). As already seen during dRT‐DC measurements, the 6‐arm HMPs did not exhibit strong deformation and in fact remained almost spherical with all conditions showing a mean deformation of around 0.02 and thus at the lower detection threshold of RT‐DC given the applied flow rate (full data presented in Figure S26). Based on numerical simulations for fully elastic spheres, RT‐DC is used to determine apparent Young's moduli from such deformation data [61, 62]. As the DMA data suggests that our DNA‐HMPs behave as viscoelastic solids, we can also extracted their Young's moduli from the RT‐DC. We find that Young's moduli determined with RT‐DC are in good agreement with the microindentation data for the 3‐arm short and 6‐arm flexible HMPs (Figures S27/S28).

While DMA by microindentation allowed us to investigate the rheological properties of the DNA‐HMPs on the second time scale, RT‐DC allows us to study relaxation on the millisecond time scale. Based on both the response time τ, extracted by fitting the dynamic RT‐DC data, and Young's modulus *E* we were able to calculate apparent viscosities η of the DNA‐HMPs following τ=η/E as 0.167Pas (3‐arm short) and 0.211Pas (6‐arm flexible) [63]. DNA‐HMPs thus show a mostly elastic response to the applied forces exhibiting low, but measurable, viscosity.

Similar analysis of the DNA‐HMPs at 20 μM DNA nanostar concentration revealed slower relaxation times and thus a decreased elasticity, well in line with our data gathered by DMA (Figures S29/S30).

Given the low deformation of the 6‐arm HMPs during RT‐DC at the tested flow rate of 0.04μL/s, accurate stiffness determination is not possible for this design under these conditions. Nevertheless, we obtain the highest stiffness values for the 6‐arm DNA‐HMPs (Figure S31), consistent with microindentation. To obtain higher deformation forces and thus more accurate values, we increased the flow rate by a factor of 10 to 0.4μL/s. We then obtain a viscosity of 0.09Pas for the 6‐arm DNA‐HMPs (Figure S32).

We were not able to fit the shape of the soft DNA‐HMPs of the 3‐arm and 4‐arm designs accurately owing to their low optical contrast. Similar findings have been reported for polyacrylamide hydrogel microparticles below a stiffness of 700Pa [20].

### 2.4 Biofunctionalization of DNA‐HMPs for Applications in a 3D Cellular System

**FIGURE 4:**
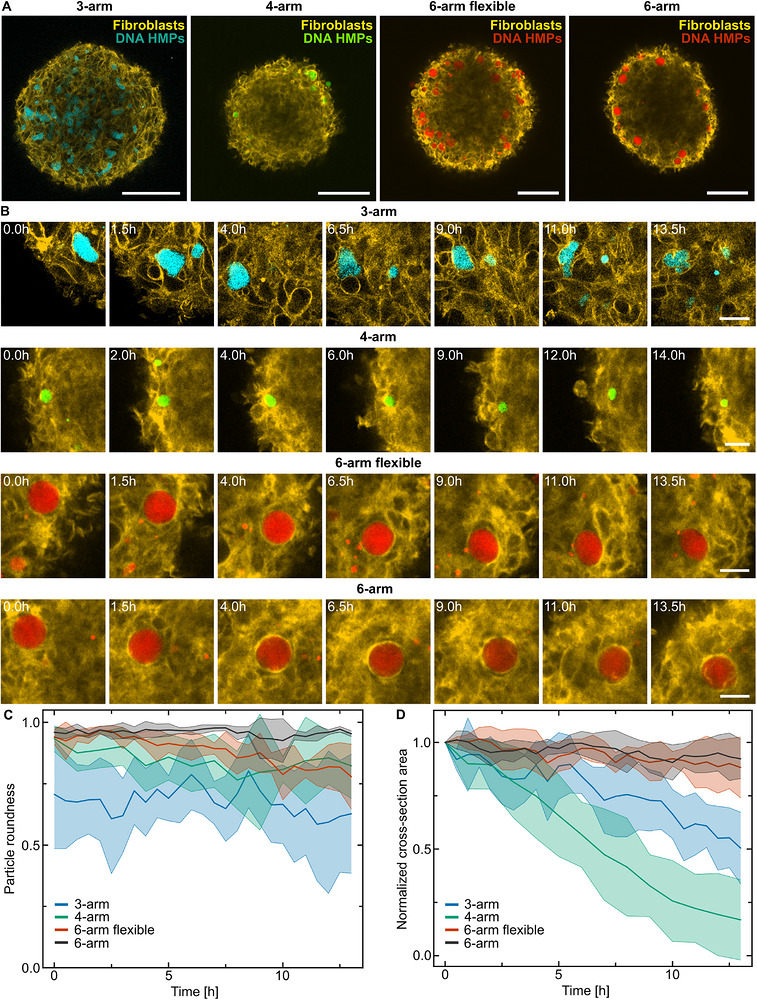
Integration of DNA‐HMPs into 3D fibroblast spheroids. (A) Confocal fluorescence microscopy of fibroblast spheroids ($\lambda_{ex}=561$ nm, td‐Tomato, yellow) with embedded 3‐arm ($\lambda_{ex}=405$ nm, ATTO‐390‐labeled DNA, cyan), 4‐arm ($\lambda_{ex}=488$ nm, ATTO‐488‐labeled DNA, green), 6‐arm flexible ($\lambda_{ex}=640$ nm, ATTO‐647N‐labeled DNA, red) and 6‐arm ($\lambda_{ex}=640$ nm, ATTO‐647N‐labeled DNA, red) DNA‐HMPs after 48 h of hanging drop co‐culture. Scale bars: 100μm. (B) Equatorial z‐slices of 3‐arm, 4‐arm, 6‐arm flexible and 6‐arm DNA‐HMPs embedded in 3D fibroblast spheroids showing particle deformation observed over time. Scale bars: 20μm. (C) Particle roundness of DNA‐HMPs inside 3D fibroblast spheroids extracted from 2D z‐slices plotted over time. (D) Cross‐sectional area of DNA‐HMP equatorial planes extracted from 2D z‐slices over time, normalized to the first time‐point. The data in C and D is presented as mean ± standard deviation per DNA nanostar design measuring five DNA‐HMPs from three separate spheroid experiments each.

The transduction of mechanical information is a major driver in biological systems during developmental processes, tumor progression and tissue homeostasis steering cellular behavior [49, 64, 65, 66]. Studying how forces are experienced and exerted in 3D cell cultures is thus vital for our understanding of cellular behavior both in growing tissues as well as in disease progression. Tools enabling local probing of these forces are continuously being developed. To this end, gel‐like particles have been used as non‐invasive in situ force sensors in 3D cell culture [11, 19, 20, 21, 22]. We thus examined whether DNA‐HMPs can be used to probe mechanical stress in 3D cellular systems as the basis for future applications. We integrated DNA‐HMPs of the 3‐arm, 4‐arm, 6‐arm flexible and 6‐arm designs into 3D fibroblast spheroids. For this, mouse liver fibroblasts expressing td‐Tomato were co‐cultured for 48 h with DNA‐HMPs in hanging drops [67] (Figure 4A, Videos S3–S6). DNA‐HMP uptake by the spheroids was facilitated by addition of the short fibronectin‐derived peptide sequence c[RGD] to the DNA linkers. c[RGD]‐tagged DNA linkers were created using Dibenzocyclooctyne (DBCO)‐azide click chemistry of DBCO‐tagged DNA single strands (see Table S1) and an azide‐modified c[RGD] moiety. Formation of the c[RGD]‐tagged DNA linkers was confirmed using polyacrylamide gel electrophoresis (PAGE, Figure S33). We further functionalized the DNA linkers with the fluorescence probe 5‐FAM to use it as a proxy to estimate c[RGD]‐distributions within the DNA networks (Figure S33). FRAP measurements show stable incorporation of the modified strands into the networks (Figures S34/S35). We estimate an average c[RGD] spacing of 8–28nm on the HMP surface (Figure S36, Note S2). Note that a spacing of 10nm has been reported to be optimal given the size of integrin at 8–12nm [68], and good cell attachment has been shown until an RGD‐spacing distance of 70nm [69, 70]. The ligand density on our DNA‐HMPs should thus be well within the biologically relevant range.

Spheroids developed normally in the presence of DNA‐HMPs, which were stable inside the spheroids during the 48 h growth period. The DNA‐HMPs were not expelled from the spheroids during the subsequent observation time, showing their stability in 3D cell culture.

Anaylsis of 2D z‐slices of the DNA‐HMPs during continuous observation for several hours revealed that DNA‐HMPs are deformed by the surrounding mouse fibroblasts. While 3‐arm HMPs are strongly deformed, 4‐arm HMPs remain more spherical, but are still compressed over time. The 6‐arm flexible and 6‐arm HMPs retained a more spherical shape, with the stiffest design showing minimal deformation (Figure 4B, Videos S7–S10). Analysis of the roundness (Figure 4C) and the compression (Figure 4D) of individual DNA‐HMPs inside the spheroids is consistent with their respective rheological properties. The softer DNA‐HMPs decrease their cross‐sectional area with time, indicating that an increasing compressive stress generated by fibroblasts is sufficient to compact the HMPs. By contrast, the stiffer 6‐arm DNA‐HMPs maintain the same cross‐sectional area over the 14 h observation period (Figure 4D). The strong decrease in size of the 4‐arm HMPs compared to the 3‐arm design may be explained by the difference in viscosity between the two designs. As the 3‐arm HMPs are more viscous in nature, they seem to show rather complex remodeling instead of the plastic compaction observed for the 4‐arm HMPs. Due to their soft nature, the 4‐arm HMPs get compacted rather than sheared like the 3‐arm design, which can also explain their relative roundness over time. Note that DNA‐HMPs that were not integrated into speroids but remained free in the cell culture medium were intact and remained undeformed during the whole imaging period (Figure S37).

To better understand DNA‐HMP deformation in the context of the compressive forces present in the proliferating spheroids [11, 71] and approximate traction forces on the particles, we additionally segmented the 3D volumes of DNA‐HMPs at 0 h (i.e., 48 h after spheroid formation) and 9 h into the experiment. DNA‐HMPs were chosen to be at least 10μm in size, while showing only small deformations (i.e. deformation ≪ particle diameter), so that we can assume linear mechanical behavior for the analyzed particles.

Comparing DNA‐HMP shapes to a perfect sphere of the same volume, we were able to infer both the normal (compressive) stress resulting in DNA‐HMP volume change [11], as well as the deviatory (shear) stress resulting in shape modification of the particles [72]. We then calculated the total traction on the DNA‐HMPs from these stresses taking particle stiffness into account. We find good agreement with our initial analysis in that softer DNA‐HMPs (4‐arm, 6‐arm flexible) show both higher elongation at 0 h and further increased elongation at 9 h than the 6‐arm DNA‐HMPs, consistent for higher strain in the softer designs (Figure S38A). Interestingly, we measure higher traction forces on the 6‐arm DNA‐HMPs (Figure S38B). This result is in line with observations of traction forces measured in 2D showing fibroblasts exert higher forces on stiffer substrates [73, 74], as well as with 3D studies showing increased traction and better cell‐attachment on stiffer particles [19, 22].

We also analyzed DNA‐HMP deformation as a function of their position inside the spheroids by normalizing the radial position of the periphery as 1 and the center as 0. Consistent with previous results [71], we find that DNA‐HMPs tend to be more deformed toward the periphery of the spheroid, suggesting a gradient in compressive stress or higher mechanical activity at the spheroid periphery compared to the core. As before, softer DNA‐HMPs deform more than stiffer ones (Figure S39).

## 3 Conclusion

In this work, we show the formation and characterization of DNA‐HMPs for 3D cell culture applications. We demonstrate that their size, stiffness, viscoelasticity and functionalization are controllable by sequence design alone; a property which is difficult to achieve to the same extent with conventional hydrogel systems. In particular, the Young's modulus of our DNA‐HMPs is finely programmable across three orders of magnitude from 30Pa to 6.5kPa.

Importantly, our results show a clear correlation between DNA nanostar design and resulting network properties. We identify DNA nanostar valency as the key driver governing network connectivity, size and material properties. As such, DNA networks show both an increase in size as well as in elastic moduli as nanostar valency increases. Nanostar valency is further linked to overall network viscosity as higher valency also results in lower viscous dampening. This behavior can further be fine‐tuned by adapting linker flexibility and linker length. DNA hydrogel mechanical properties are thus drastically altered by minor sequence changes of only two nucleotides, outlining a vast design space of DNA hydrogels which could lead to evolvable “Darwinian” materials. Finally, we show that c[RGD]‐functionalized DNA‐HMPs are uptaken into 3D spheroid structures. In such compressive 3D cell culture settings, DNA‐HMPs of different valency show differing behavior. An increase in nanostar valency results in an increase in applied tractions, while network elongation and volume compression decrease. Given their viscoelastic nature, DNA‐HMP mechanical properties are non‐linearly dependent on the applied load and time of pressure application. As such, accurate quantification of forces would require the formulation of a viscoelastic model tailored to the DNA‐HMPs specifically. The presented tractions consequently present approximations. Nonetheless, our analysis reveals trends consistent with previous literature [11, 19, 22, 71, 73, 74], showcasing the potential of DNA‐HMPs for mechanical readouts.

As DNA‐HMPs can be delivered into 3D cellular systems in a completely non‐invasive manner thanks to the functionalization with cell‐binding peptides, this opens up the possibility for their further usage in other 3D or 2D multicellular systems. Combined with the already established stimuli‐response system for the release of cargo [6], such DNA‐HMPs could offer a unique multi‐functionality whereby the particles allow for locally‐confined chemical perturbations in combination with a mechanical readout. Given the ease of design, the self‐assembly of DNA hydrogel structures and the myriad of possible stimuli‐responses such as temperature, light, pH or protein interaction inherent to DNA, DNA‐HMPs could become powerful tools for the further study and manipulation of engineered cellular systems, and in vivo models.

## 4 Experimental Section

### 4.1 Design and Handling of DNA Sequences

The design and sequence of the DNA strands used for 3‐arm short nanostars A and B as well as the linker strand were adapted from previous publications [36, 37]. The sequences for the 3‐arm nanostars C and D were designed based on these by elongating the linker and the respective sticky‐end overhangs by three additional nucleotides each.

The 4‐arm nanostars were based on the nanostar design from a previous publication [43] and adapted to contain the sticky‐end overhangs for the elongated linker. Likewise, the 6‐arm nanostars were based on another structure from a previous publication [41], adapted to contain sticky‐end overhangs and a corresponding linker of the same length as the elongated linker. Melting curves and binding behavior of all structures were analyzed and verified using NuPack [44]. All DNA was purchased either from Integrated DNA Technologies (unmodified DNA, purification: standard desalting) or Biomers (modified DNA, purification: HPLC). All unmodified DNA was diluted in 10 mM Tris (pH = 8) and 1 mM EDTA (Sigma Life Science) to yield 800 μM stock solutions. Modified strands were diluted in MilliQ water to yield 800 μM stock solutions. DNA concentrations were measured using a nanodrop device (Implen). All DNA sequences are listed in Table S1. The DNA stock solutions were stored at $-20^{\circ }C$, if not in use.

### 4.2 Preparation of 3‐arm Short and 3‐arm DNA Nanostars

Annealing of the DNA nanostars (A/B and C/D) was achieved by mixing the three respective DNA single‐strands A‐1, A‐2, A‐3 or B‐1, B‐2, B‐3; C‐1, C‐2, C‐3 or D‐1, D‐2, D‐3 at equimolar ratios resulting in 150 μM solutions of the DNA nanostars A and B or C and D. For confocal fluorescence microscopy, 4 mol% of cyanine‐3 (Cy3)‐labeled strands (B‐1‐Cy3 and D‐1‐Cy3) or Atto‐390‐labeled strands (D‐1‐390) were added (for FRAP experiments, concentration of the fluorescent strands was modified as outlined in the respective figure captions). Phosphate‐buffered saline (PBS, no CaCl_2_, no MgCl_2_, pH = 7.4; Gibco) solution at a final concentration of 1x, as well as a final concentration of 10 mM of MgCl_2_ were added for annealing. The nanostars were annealed in a thermal cycler (BioRad) by heating the samples to $95^{\circ }C$ for 3min and subsequently cooling to $20^{\circ }C$ at an increment rate of $0.1^{\circ }C$/s. If not stated otherwise, 1x PBS and 10 mM MgCl_2_ were used as buffer conditions for all experiments.

### 4.3 Preparation of 4‐arm DNA Nanostars

Annealing of the 4‐arm DNA nanostars (E/F) was achieved by mixing the four respective DNA single‐strands E‐1, E‐2, E‐3 and E‐4 or F‐1, F‐2, F‐3 and F‐4 at equimolar ratios resulting in 150 μM solutions of the 4‐arm design. For confocal fluorescence microscopy, 4 mol% of an Atto‐488‐labeled DNA strand (E‐1‐488) were added to the E monomer solution (for FRAP experiments, concentration of the fluorescent strands was modified as outlined in the respective figure captions). Phosphate‐buffered saline (PBS, no CaCl_2_, no MgCl_2_, pH = 7.4; Gibco) solution at a final concentration of 1×, as well as a final concentration of 10 mM of MgCl_2_ were added for annealing. The 4‐arm design was annealed in a thermal cycler (BioRad) by heating the samples to $95^{\circ }C$ for 10min and subsequently cooling to $20^{\circ }C$ at an increment rate of $0.6^{\circ }C$/min.

### 4.4 Preparation of 6‐arm DNA Nanostars

Annealing of the 6‐arm DNA nanostars (G/H) was achieved by mixing the six respective DNA single‐strands G‐1, G‐2, G‐3, G‐4, G‐5, and G‐6 or H‐1, H‐2, H‐3, H‐4, H‐5 and H‐6 at equimolar ratios resulting in 117 μM solutions of the 6‐arm design. For confocal fluorescence microscopy, 4 mol% of an Atto‐647N‐labeled DNA strand (G‐1‐647) were added to the G monomer solution (for FRAP experiments, concentration of the fluorescent strands was modified as outlined in the respective figure captions). Phosphate‐buffered saline (PBS, no CaCl_2_, no MgCl_2_, pH = 7.4; Gibco) solution at a final concentration of 1x, as well as a final concentration of 10 mM of MgCl_2_ were added for annealing. The 6‐arm design was annealed in a thermal cycler (BioRad) by heating the samples to $95^{\circ }C$ for 10min and subsequently cooling to $20^{\circ }C$ at an increment rate of $0.6^{\circ }C$/min.

### 4.5 Preparation of Microfluidic Chips

All microfluidic chips were designed using the CAD software QCAD‐pro (Ribbonsoft, Switzerland). The channels are 30μm (standard) or 60μm (only for the largest HMP in Figure 1) in height and width respectively. SU8‐3025 negative photoresist (MicroChem, USA) was spin‐coated (Laurell Technologies Corp., USA) at 2600 rpm for 30s to achieve a 30μm uniform layer in height on 2‐inch silicon wafers (MicroChemicals, Germany), twice that for the 60μm channel. The designed structures were then exposed to the photoresist‐coated wafer by using the Tabletop Micro Pattern Generator μMLA (Heidelberg Instruments, Germany) with $375\;mJ/cm^{2}$. The wafer was baked at $65^{\circ }C$ for 1min and for another 5min at $95^{\circ }C$ to harden the exposed regions and developed afterward to remove non‐exposed photoresist with a developer (mr‐DEV 600, MicroChemicals, Germany). A hard bake was performed at $150^{\circ }C$ for 30min. Microfluidic chips were produced using soft lithography. Briefly, Polydimethylsiloxane (PDMS, Sylgard 184, Dow Corning, USA) was prepared in a 9:1 (w/w) ratio (oligomer:polymerization catalyst). The solution was used to cover the wafer and hardened at $65^{\circ }C$ for at least 2 h following degassing of the PDMS‐solution under vacuum. The PDMS block was then cut out, and connection holes for the inlets and outlets of 0.5mm size (Biopsy Punch, World Precision Instruments, USA) punched. As a final step the PDMS block was cleaned with 70% EtOH and activated in an oxygen plasma (300 Semi‐auto plasma processor (PVA TePla AG 0.5 mbar, 200W, 30s)) together with a coverslip (Carl Roth, Germany, 24× 60 mm) and bonded. The bonded chips were incubated at $65^{\circ }C$ overnight and stored at room temperature until further usage. Prior to DNA‐HMP production, the microfluidic channels were coated using a perfluorinated oil (Aquapel).

### 4.6 Preparation of Droplet‐Templated DNA‐HMPs Using the 3‐arm Short Design

DNA‐HMPs were produced in a water‐in‐oil droplet‐templated manner following encapsulation of the DNA‐containing solution into water‐in‐oil droplets. Water‐in‐oil droplets were either produced by microfluidics (for the data shown in Figure 1D) to ensure uniform droplet sizes, or by manual shaking (for all other presented data).

For water‐in‐oil droplet production in a microfluidic setup, channels containing a flow‐focusing T‐junction were used. For DNA‐HMPs of up to 30μm in size, a double‐inlet microfluidic chip was used (channel design enclosed as Figure S1A). Here, the nanostars A and B were supplied at equimolar concentrations (20 μM each) in one channel, while the linker strand was supplied through the second inlet at a 3x higher concentration to ensure gel‐formation (60 μM linker strand for 20 μM DNA nanostars). The DNA solutions were supplied using a syringe pump system (NemeSys, Cetoni). Mixing of the two phases and thus gel‐formation was facilitated upon encapsulation into water‐in‐oil droplets at the water‐oil junction. To ensure equal mixing of both nanostars and linker strands, both aqueous streams were injected using the same flow rates. As the oil‐phase, a solution of 2 wt% perfluoropolyether–polyethylene glycol (PFPE–PEG, RAN Biotechnologies) dissolved in HFE‐7500 (Iolitex Ionic Liquids Technologies) was supplied using a separate syringe pump (World Precision Instruments) at a ratio of 4:1 oil to aqueous flow rates (e.g. 0.4μL/s oil:0.1μL/s aqueous phase). For DNA‐HMPs smaller than 30μm, up to three times higher oil flow rates were applied. DNA‐HMPs larger than 30μm were produced in a single‐inlet microfluidics device (channel design enclosed as Figure S1B) using an air‐pressure‐based elveflow control microfluidics system (Elveflow). Here, the linker strands were added to the nanostar‐containing solution directly before applying it to the device to keep the gelation time outside of the droplets minimal. The aqueous and oil solutions were supplied in a ratio of 300 to 200 mbar of the PFPE‐PEG containing oil phase and the sample‐containing aqueous phase, respectively.

Alternatively, water‐in‐oil‐droplets containing the same oil and aqueous phase were created by adding the aqueous solution on top of the oil phase in a volumetric ratio of 1:3 in a microtube (Eppendorf, typically 50μL aqueous to 150μL oil phase) and flicking the tube with a finger 8 times [38].

The droplets were then stored at $22^{\circ }C$ room temperature for 72 h to allow the DNA‐HMPs inside the droplets to fully assemble. After that, DNA‐HMPs were released from the water‐in‐oil emulsion by first adding the buffer in which the DNA‐HMPs were formed (always: 1× PBS and 10 mM MgCl_2_) at 1.5× the initial volume on top of the droplet emulsion and subsequently breaking the emulsion. To break the droplet emulsion, 1H,1H,2H,2H‐Perfluoro‐1‐octanol (Merck) was added on top of the buffer and droplet emulsion, and incubated for 30min. Released DNA‐HMPs in solution were then transferred to a new microtube for use. The resulting DNA‐HMPs were stored in 1× PBS and 10 mM MgCl_2_ buffer at $4^{\circ }C$. DNA‐HMPs were prepared fresh for every experiment.

### 4.7 Preparation of 3‐arm, 4‐arm and 6‐arm Flexible DNA‐HMPs

3‐arm, 4‐arm and 6‐arm flexible DNA‐HMPs were created similarly to the 3‐arm short DNA‐HMPs following the same process of templated formation of DNA‐HMPs in water‐in‐oil‐droplets. Here, only manual shaking was used to form the DNA‐HMPs presented in this study.

For the formation of 3‐arm DNA‐HMPs, the 3‐arm nanostars C and D were mixed at equimolar ratios (e.g. 20 μM each) and the addition of 3x this concentration of the elongated linker (e.g. 60 for 20 μM DNA‐HMPs).

For the formation of 4‐arm DNA‐HMPs, the 4‐arm nanostars E and F were mixed at equimolar ratios (e.g. 20 μM each) and the addition of 4× this concentration of the elongated linker (e.g. 80 for 20 μM DNA‐HMPs).

Likewise, 6‐arm flexible DNA‐HMPs were prepared following the same experimental setup, but using equimolar concentrations of the nanostars G and H and 6x the corresponding concentration of the flexible 6‐arm‐linker (e.g. 120 for 20 μM DNA‐HMPs). To achieve even gelation, monomer H and the flexible 6‐arm‐linker were mixed into the solution first and allowed to bind for 10min. As a last step before encapsulation, the G monomer was added.

All resulting DNA‐HMPs were stored in 1× PBS and 10 mM MgCl_2_ buffer at $4^{\circ }C$. DNA‐HMPs were prepared fresh for every experiment.

### 4.8 Preparation of 6‐arm DNA‐HMPs

Formation of the 6‐arm DNA‐HMPs was likewise achieved using droplet‐templated formation in water‐in‐oil droplets using the monomers G and H and the 6‐arm‐linker in 6x excess. The droplets containing the DNA‐solution were then placed into a thermal cycler (BioRad) and the DNA inside annealed by heating the samples to $80^{\circ }C$ for 15min and subsequently cooling to $20^{\circ }C$ at an increment rate of $0.1^{\circ }C$/min. Note that in order to prevent droplet fusion during heating in the thermal cycler, the surfactant perfluoropolyether–polyethylene glycol (PFPE–PEG, RAN Biotechnologies) was applied at 5 wt% instead of 2 wt% concentration.

The resulting DNA‐HMPs were stored in 1× PBS and 10 mM MgCl_2_ buffer at $4^{\circ }C$. DNA‐HMPs were prepared fresh for every experiment.

### 4.9 Confocal Fluorescence Microscopy

For imaging, an LSM 900 Zeiss confocal fluorescence microscope (Carl Zeiss AG) was used. For each experiment, the pinhole size was set to one Airy unit and either a Plan‐Apochromat 20×/0.8 Air M27 objective or a 63×/1.2 W korr objective with oil immersion were utilized. If not stated otherwise, experiments were conducted at $22^{\circ }C$ room temperature. For imaging, the DNA‐HMPs were deposited into custom‐built observation chambers made from glass slides (Carl Roth) attached via double‐sided sticky tape (Tesa) and sealed using two‐component glue (twin‐sil, Picodent). Prior to assembly of the observation chamber, the glass slides were coated for 5min with poly(vinyl‐alcohol) (50mg/mL, Sigma Aldrich). For image analysis, Fiji‐ImageJ 1.54f (NIH, [75, 76]) was employed.

### 4.10 Analysis of DNA‐HMP Size Distributions and Long‐Term HMP Stability

Analysis of DNA‐HMP size distributions was conducted using the open‐source software Fiji‐ImageJ 1.54f [75, 76]. Confocal fluorescence microscopy images were processed by first applying a Gaussian‐filter to smooth particle outlines (σ = 2.0) followed by thresholding using Otsu's method. The images were then binarized and a watershed algorithm applied to separate DNA‐HMPs too close to one another. Particle size was then measured using “Analyze Particles” setting the size range to $50\;\mu m^{2}$ to infinity and the circularity to 0–1.0 saving Feret's diameter and particle aspect ratio as size readouts. DNA‐HMPs at the image‐edge, which were only partially detected by this measurement, were discarded from the dataset.

The same analysis was conducted to analyze DNA‐HMP stability across time. Here, particle aspect ratio and mean intensity were collected as readouts. Further, background fluorescence was measured by selecting a square area of roughly 1300 px^2^, measuring in two position per imaged frame. The mean intensity of these was then averaged and used as background fluorescence. Dividing this by the mean fluorescence across all analyzed DNA‐HMP then gave the intensity ratio.

### 4.11 Fluorescence Recovery After Photobleaching

Fluorescence recovery after photobleaching (FRAP) experiments were conducted and analyzed as previously outlined [37]. In short, the region‐of‐interest size was set to 10 or 15μm and the spot irradiated for 200 iterations at a frame rate of 300ms with the corresponding laser for each utilized dye to bleach the fluorophores. The bleached spot was subsequently imaged for at least 200 frames and the fluorescence intensity in the bleached region recorded. Laser power was set to 5 or 1mW depending on the experiment, while for FRAP experiments at higher temperature, the confocal was heated to $37^{\circ }C$. For FRAP experiments showing the diffusion‐based recovery of Hoechst and ATTO‐647N‐labeled G nanostars inside the DNA‐HMP network, 30 μM 3‐arm short DNA‐HMPs were incubated for 30min either with Hoechst34580 (Sigma Aldrich) at a final concentration of 20 mg/L or labeled G nanostar at a final concentration of 1 μM. A region‐of‐interest of 15μm was then set and bleaching conducted at room temperature as discussed above.

### 4.12 Analysis of DNA‐HMP Formation Over Time

The formation of DNA‐HMPs (20 μM concentration, 3‐arm short design) was tracked via confocal fluorescence microscopy over the course of 2 h using 5× digital zoom and 4x line averaging at an interval of 30s. Per experiment, the formation of one DNA‐HMP was tracked this way and the experiment conducted three times. The area fraction of the fluorescent signal inside the water‐in‐oil droplets was then analyzed using Fiji‐ImageJ 1.54f (NIH, [75, 76]). The images of the individual droplets were then binarized using Otsu's method. Utilizing the circle tool to cover the whole imaged droplet, the area fraction of the fluorescent pixels over all pixels within the selected area was measured for each frame. The measured area fractions were normalized to the first frame of each video and the average of these values (n = 3 DNA‐HMPs ± standard deviation) plotted over time.

Next, the formation of the DNA‐HMPs following the condensation of DNA aggregates formed across the whole water‐in‐oil droplet was analyzed. For this, all frames were filtered using a Gaussian blur filter (σ = 2.0) to improve the signal to noise ratio. Using the plot profile function in Fiji‐ImageJ 1.54f (NIH, [75, 76]), whole droplet profiles were taken at nine different time points across the recorded experiment using the square tool to encase the whole droplet. Profiles were plotted in OriginPro 2021 ‐ Update 6 (Origin Lab Corporation) and their peaks counted and analyzed using its Peak Analyzer tool to get the number of peaks and the gray value at each peak's center for every recorded profile, which were then plotted over the recorded time.

### 4.13 Cryogenic Scanning Electron Microscopy (CryoSEM)

CryoSEM sample preparation was performed as previously described [77]. Briefly, 3μL of a DNA‐HMP solution was deposited onto a 0.8mm‐diameter gold specimen carrier mounted on a freeze‐fracture holder (Leica Microsystems) and immediately immersed in liquid nitrogen. The cryo‐embedded HMPs were then transferred using an evacuated, liquid nitrogen‐cooled shuttle (Leica EM VCT100, Leica Microsystems) into a freeze‐fracture and etching system (Leica EM BAF060, Leica Microsystems). Fracturing was carried out in a vacuum chamber (10^−6^ to 10^−7^ mbar) at $-160^{\circ }C$ using a cooled knife. To enable sublimation of water from the fractured HMPs, the sample stage was subsequently heated to $-90^{\circ }C$ for 45 min. After sublimation, the freeze‐fractured HMPs were coated with a 4nm layer of platinum/carbon (Pt–C) by electron beam evaporation. For image acquisition, the samples were transferred via the same liquid nitrogen‐cooled shuttle into the imaging chamber of a field‐emission scanning electron microscope (FE‐SEM; Zeiss Ultra 55, Carl Zeiss Microscopy), equipped with in‐lens, secondary electron (SE), and angle‐selective back‐scattered electron (ASB) detectors (Carl Zeiss SMT). Top‐view imaging was performed under cryogenic conditions (stage temperature $-115^{\circ }C$ ± $5^{\circ }C$) with a working distance of 3–5mm. Due to the low conductivity of the HMPs, low acceleration voltages of 1.5–4.0kV were used. Signals were detected using the in‐lens detector.

### 4.14 DNA‐HMP Stability During Heating

To study DNA‐HMP behavior at increased temperatures, samples were prepared in observation chambers as outlined above. Using a thermal cycler, the observation chambers were heated for 10 min to the desired temperature prior to confocal microscopy by placing the observation chambers directly on the heated wells. Images were acquired immediately after taking the samples out of the thermal cycler to keep cooling to a minimum.

### 4.15 Analysis of DNA‐HMP Size Compared to Water‐in‐Oil Droplets

To analyze DNA‐HMP size as a function of water‐in‐oil droplet size, confocal fluorescence microscopy images of the DNA‐HMPs inside of the water‐in‐oil droplets after 72 h of incubation were acquired. A custom‐written Fiji macro was used for the analysis. To detect the water‐in‐oil droplets in the brightfield, the images were filtered using a variance filter of radius 2, inverting the image and thresholding to create binary images using Huang's method as implemented in Fiji‐ImageJ 1.54f (NIH, [75, 76]). Using Analyze particles from $50\;\mu m^{2}$ ‐ Infinity in size and 0.20–1.00 in circularity, water‐in‐oil droplets were detected and their size as area, and position stored. The fluorescence channel containing the DNA‐HMP signal was then likewise filtered and thresholded using Gaussian blur filtering (σ = 2.0), Otsu's method for thresholding and particles analyzed in the same way as for the water‐in‐oil droplets. To match positions of DNA‐HMPs inside water‐in‐oil droplets, both size and position of the DNA‐HMP were stored. The fraction of DNA‐HMP size vs water‐in‐oil droplet size was then calculated for each DNA‐HMP. Experiments were conducted in triplicates per condition using DNA‐HMPs at 30 μM DNA nanostar concentration.

### 4.16 Sample Preparation and Workflow for Microindentation

24 Well Glass bottom Plates (Cellvis) were treated with oxygen plasma under vacuum (0.5 mbar final pressure, 200W) for 3min using a 300 Semi‐auto plasma processor (PVA TePla AG). The wells were next filled with 1mL of a 0.5 mg/mL poly‐l‐lysine (MW = 150–300 kDa, Sigma Aldrich) solution in MilliQ water, and incubated for 60min. The wells were then washed three times using 1× PBS and 10 mM MgCl_2_ solution. 100μL of DNA‐HMPs in solution were added to a total volume of 1 mL 1× PBS and 10 mM MgCl_2_ in the wells and allowed to settle and adhere to the poly‐l‐lysine coated wells for 30min before washing the wells three times using 1x PBS and 10 mM MgCl_2_ solution. Indentation experiments were conducted on a Pavone microindenter (Optics11Life) using dynamic mechanical analysis (DMA) in displacement mode. For the single particle measurements, a cantilever with a tip size of 3.5μm and a spring constant of 0.019N/m was used. DMA was conducted at 0.1, 0.5, 1, 2, 5, 10 and 20 Hz frequency applying an indentation amplitude of 100nm, 2s relaxation time between frequencies and an initial relaxation time of 10s. For each sample, measurements on five separate DNA‐HMPs were undertaken. Each condition was measured as triplicates. Calculation of the Young's moduli was based on Hertz‐model fits of the indentation curve fitted to an indentation depth equal to 16% of the cantilever‐tip diameter (560nm). Further, each sample was indented no further than 5% of the sample diameter, to exclude any measurement artifacts based on the underlying substrate. Prior to conducting a measurement, the cantilever was lowered in small increments of 500nm toward the DNA‐HMPs until it showed a deflection smaller than 500nm, detecting the particle surface. DMA measurements were then conducted by lifting the cantilever up above the particle and moving the indentation spot by at least 1μm before running the indentation experiment as outlined above. Particles which were indented further than 500nm during this approaching step were not included in the final dataset, while particles which moved before, during or after indentation were likewise excluded. Measured particles were thus only indented once to the measuring depth and at the measuring position, as well as never more than twice total. This way alterations to the material integrity were kept at the necessary minimum. The data is presented as the mean ± the standard deviation of the resulting values for the Young's modulus. Storage, loss moduli and tan(δ) of the different types of DNA‐HMPs are presented as single data points. Data analysis was conducted using the analysis software DataViewer (V2.5.0, Optics11Life), while plotting of the data as well as the statistical analysis were managed using OriginPro 2021 ‐ Update 6 (Origin Lab Corporation).

### 4.17 Measurement of Hydrodynamic Drag Forces During Microindentation

Oscillating cantilevers in viscous medium experience a force even in the absence of physical contact with a surface due to hydrodynamic drag [78, 79]. This drag depends on the viscosity and density of the medium, the size of the cantilever, and the frequency of oscillation. Thus, it needs to be determined for any given set of experimental parameters, as it can otherwise be mistakenly attributed to the properties of the material being tested during DMA. We thus measured the hydrodynamic drag force experienced by the utilized cantilever to estimate its influence on DMA measurements and corrected the data accordingly. For this, the cantilever tip was placed 500nm above a DNA‐HMP and the DMA sweep from 0.1–20 Hz performed there to estimate the viscous drag. This measurement was repeated five times to yield an average drag force measurement. For a detailed description on the correction of the drag force, see Note S1 and Figures S40/S41.

### 4.18 Sample Preparation and Workflow for Real‐Time Deformability Cytometry

Real‐time deformability cytometry (RT‐DC) was performed using an AcCellerator (Zellmechanik Dresden) on an inverted AxioObserver microscope (Carl Zeiss AG) equipped with a 20×/0.4 Ph2 Plan‐NeoFluar objective (Carl Zeiss AG). Images were acquired using a high‐speed CMOS‐camera (MC1362, Microtron).

Right before each measurement, DNA‐HMPs in suspension (100μL per run) were pelleted for 1min using a C1008‐GE myFUGE mini centrifuge (Benchmark Scientific). 80μL of supernatant were discarded and the pellet resuspended in 150μL CellCarrierB (Zellmechanik Dresden). The resuspended DNA‐HMPs were then aspirated into a 1mL glass syringe with PEEK tubing connector and PTFE plunger (SETonic) mounted on a syringe pump system (NemeSys, Cetoni). The DNA‐HMP‐CellCarrierB solution was then applied to a Flic20 microfluidic chip (Zellmechanik Dresden) using PTFE‐tubing (S1810‐12, Bola). Through a second 1mL glass syringe, CellCarrierB was applied to the Flic20 microfluidic chip as sheath flow for the RT‐DC experiment. For all samples, two sequential measurements using 0.04μL/s and 0.08μL/s (0.4μL/s additionally for 6‐arm DNA‐HMPs) total flow rates (ratio of sheath to sample flow: 3:1) were performed for a duration of at least 900s each. The measurement software ShapeIn (version 2.2.2.4, Zellmechanik Dresden) was used to detect DNA‐HMPs in real time. The pixel‐size was adjusted to 0.68 μm/px, fitting the utilized 20×/0.4 Ph2 objective and all DNA‐HMPs imaged at the rear part of the flow channel ensuring regular deformation of each particle. For each condition triplicates were measured. The analysis software Shape‐Out (version 2.10.0, Zellmechanik Dresden) was then used to analyze the behavior of the DNA‐HMPs in the flow channel. All samples were equally gated for porosity (1.0–1.15) and DNA‐HMP size (40–$180\;\mu m^{2}$). Statistical significance was analyzed using a linear mixed model (R‐lme4) as integrated in Shape‐Out (version 2.10.0 [80]) yielding ANOVA p‐values. Calculation of the Young's modulus, deformation and volume as well as preparation of the data for contour plots were all carried out using Shape‐Out (version 2.10.0). Population mean values of the measured samples are presented with their standard error of the mean. Plots for the volume, deformation and Young's modulus were created using OriginPro 2021 ‐ Update 6 (Origin Lab Corporation).

### 4.19 Dynamic Real‐Time Deformability Cytometry

For dynamic real‐time deformability cytometry (dRT‐DC), the width of the region in which images are taken during measurement was set to the maximum of 1200 px. Likewise, the frame rate of the CMOS‐camera (MC1362, Microtron) was set to the maximum of 7000 f/s to allow for the imaging of individual particles as they undergo deformation. Sample preparation and data analysis was conducted in the same way as for the other RT‐DC experiments. Plots and data fits were created using OriginPro 2021 ‐ Update 6 (Origin Lab Corporation). To fit the deformation data of the DNA‐HMPs during dRT‐DC measurements, the speed of the particles inside of the flow channel was calculated. For this, the volume of the whole channel as $20\;\mu m\times 20\;\mu m\times 300\;\mu m=120000\;\mu m^{3}$ was taken into account. The volume of the channel divided by the flow rate of 0.04μL/s as $40000000\;\mu m^{3}/s$ resulted in a channel traversing time of 3ms for the DNA‐HMPs. Given the channel length of 300μm, the speed of the DNA‐HMPs was calculated as 100μm/ms and the data plotted accordingly. The relaxation (characteristic response) time τ as well as its standard error of the different DNA‐HMP populations were then extracted from exponential fits of the resulting deformation curves using OriginPro 2021 ‐ Update 6 (Origin Lab Corporation). For 6‐arm DNA‐HMP dRT‐DC we further ran experiments at 0.4μL/s, adjusting the relaxation time calculations accordingly.

### 4.20 Formation of c[RGD]/5‐FAM‐Tagged DNA Linker Strands

The small peptide cyclo[Arg‐Gly‐Asp‐D‐Phe‐Lys(Azide)] (c[RGD], Hölzel Diagnostika Handels GmbH) was diluted in a 1x PBS (no CaCl_2_, no MgCl_2_, pH = 7.4; Gibco) solution to yield a concentration of 2.4 mM. DBCO‐tagged DNA strands (elongated linker DBCO, 6‐arm‐linker DBCO and flexible 6‐arm‐linker DBCO) were then dissolved at a final DNA concentration of 800 μM using the c[RGD]‐solution creating a 3:1 ratio of azide to DBCO, increasing the reaction yield. The solution was then incubated at $4^{\circ }C$ for 72 h to allow the DBCO‐azide click reaction to occur. The resulting solution was then stored at $-20^{\circ }C$ until further use. The same reaction was also used to click 5‐FAM‐azide (Lumiprobe, 10 mM stock in DMSO) to the DBCO‐modified linker by diluting 5‐FAM‐azide to 2.4 mM in 1x PBS (no CaCl_2_, no MgCl_2_, pH = 7.4; Gibco) and using it in the same way as the c[RGD]‐solution.

### 4.21 Polyacrylamide Gel Electrophoresis

Polyacrylamide gel electrophoresis (PAGE) was performed as previously described [37]. Here, however, a 15% polyacrylamide gel and a final concentration of 1x PBS (PBS, no CaCl_2_, no MgCl_2_, pH = 7.4; Gibco) was used as buffer in each sample. 10μL Tridye Ultra low range DNA ladder (NEB) were used as reference. Gels were stained in 1× TBE (Tris Borate EDTA, Sigma, Thermo Scientific) buffer supplied with GelRed dye (Millipore) in a 1:10 000 dilution for 10 min. DNA samples were run at a final concentration of 10 μM each.

### 4.22 Formation of c[RGD]/5‐FAM‐Tagged DNA‐HMPs

To create c[RGD]‐tagged DNA‐HMPs, 50μL of the desired HMP suspension was pelleted for 1min using a C1008‐GE myFUGE mini centrifuge (Benchmark Scientific). Subsequently, 40μL of the supernatant were removed and the DNA‐HMP pellet resuspended using 10μL of a 20 μM solution of the respective c[RGD]‐tagged linker to yield a final concentration of 10 μM c[RGD]‐tagged linker in the DNA‐HMPs. HMPs were incubated with the linker overnight and subsequently washed 3× using a solution of 1× PBS and 10 mM MgCl_2_ to remove any non‐incorporated peptides and DNA linkers. Modification of the DNA‐HMPs with 5‐FAM‐modified linkers was conducted in the same way.

### 4.23 Analysis of 5‐FAM Fluorescence Intensity After Uptake Into DNA‐HMPs

Analysis of 5‐FAM‐signal in DNA‐HMPs was conducted using the open‐source software Fiji‐ImageJ 1.54f (NIH, [75, 76]). Confocal fluorescence microscopy images were processed by first applying a Gaussian‐filter to smooth particle outlines (σ = 2.0) followed by thresholding using Otsu's method. The images were then binarized and a watershed algorithm applied to separate DNA‐HMPs too close to one another. Particle size was then measured using “Analyze Particles” setting the size range to $50\;\mu m^{2}$ ‐ infinity and the circularity to 0.0–1.0 saving particle intensity. DNA‐HMPs at the image‐edge, which were only partially detected by this measurement, were discarded from the dataset.

Further, background fluorescence was measured by selecting a square area of roughly 1300 px^2^, measuring in two position per imaged frame. The mean intensity of these was then averaged and used as background fluorescence. Dividing this by the mean fluorescence across all analyzed DNA‐HMP in the given sample resulted in the reported ratio. Per design (3‐arm, 6‐arm flexible and 6‐arm), two field of views per replicate and a total of three replicates were analyzed. The data is represented as the mean ± error propagated standard deviation across all replicates.

### 4.24 Incorporation of DNA‐HMPs Into 3D Fibroblast Spheroids

DNA‐HMPs were incorporated into 3D fibroblast spheroids, using the hanging drop technique, as described previously [67]. Briefly, primary mouse liver fibroblasts expressing td‐Tomato (Mouse parental genotypes: TglnCreERT2/mTmG/TgfbrII lox/lox crossed with mTmG (Gt(ROSA)26Sortm4(ACTB‐tdTomato,‐EGFP)Luo/J), passage 20, mycoplasma negative) were used to form 3D cell spheroids. Every hanging drop comprised around 8500 fibroblasts and between 500 and 1000 DNA‐HMPs. Cells and DNA‐HMPs were co‐cultured in cell media (DMEM, Gibco) supplemented with 1% FBS (Gibco), 1% penicillin‐streptomycin (Gibco) and 10% methylcellulose (4000 cPs, Thermo Scientific) of 12mg/mL concentration. Hanging drops of 15μL volume were then formed at the inner part of the lid of a petri‐dish, which was incubate at $37^{\circ }C$ under 5% CO_2_ atmosphere for 2 days or overnight. After that, the spheroids were collected and embedded in an alginate matrix of 2.5mg/mL concentration, cross‐linked with 0.12 M CaSO_4_ to keep their 3D and spherical shape during imaging. Primary mouse liver fibroblasts were acquired by the Trepat laboratory as a gift from the Batlle laboratory (IRB Barcelona). Originally, the cells were provided in passage 12.

### 4.25 Imaging DNA‐HMP Deformation in 3D Fibroblast Spheroids

DNA‐HMP deformation inside 3D spheroids was tracked using a spinning disk confocal Dragonfly 200 (Andor) mounted on a Nikon Ti Eclipse microscope or a scanning confocal Zeiss LSM 900 microscope. Spheroids were captured using time‐resolved z‐stack imaging of several spheroids overnight, with frame rates of either 30, 45, or 60min with a 100μm total volume imaged per spheroid in steps of 1μm, using a 20× Nikon objective. Image processing and video generation of the time‐lapses were done using the open‐source software ImageJ 1.54f (NIH, [75, 76]).

### 4.26 Analysis of Roundness and Cross‐Section Area of DNA‐HMPs Embedded in 3D Fibroblast Spheroids

Analysis of particle roundness and cross‐section area of 4‐arm, 6‐arm flexible and 6‐arm DNA‐HMPs embedded in 3D fibroblast spheroids was conducted using Fiji‐ImageJ 1.54f (NIH, [75, 76]). Time‐resolved z‐stacks were loaded and substacks of individual DNA‐HMPs extracted. Per substack, the equatorial plane of a DNA‐HMP was extracted and the images compiled into a new stack containing the equatorial plane of the particle across time. To yield particle roundness and area, the stacks were filtered using a Gaussian‐filter to smooth DNA‐HMP outlines (σ = 1.0) and thresholded (Otsu's method), yielding binarized images. Particles were then measured using “Analyze Particles,” setting a size range of $50\;\mu m^{2}$ ‐ infinity and a circularity range of 0.0–1.0, extracting the mean gray value, area and shape descriptors of the analyzed objects. To account for the low intensity of the 3‐arm DNA‐HMPs, corresponding data of this design was processed by removing noise via despeckling, smooth‐filtering and thresholding (Otsu's method) prior to particle analysis. Per condition we analyzed five separate DNA‐HMPs across three separate spheroid experiments. The same analysis was also conducted on DNA‐HMPs outside of the fibroblast spheroids as a stability control. For this, nine DNA‐HMPs from a total of eight different experimental field of views were analyzed.

### 4.27 Segmentation and Image Processing of DNA‐HMPs to Determine Traction Forces in 3D

Prior to analysis, the z‐step of confocal fluorescence image stacks was corrected for the mismatching refractive index between the microscope's objective immersion medium and the sample, using a modified Visser's correction [81, 82]. The xy‐coordinates of the center of each spheroid was measured by fitting a circle to its contour in a horizontal plane using Fiji‐ImageJ 1.54f (NIH, [75, 76]), and its z‐coordinate by fitting another circle to its contour in a vertical plane passing through the previously calculated xy‐coordinates.

A region of 124 × 124 px was cropped around each DNA‐HMP, 3D‐deconvolved using the microscope and objective 3D Point Spread Function [83] and nearby HMPs removed. Each DNA‐HMP was then segmented, a 3D mask obtained using BeadBuddy [84] and saved as a Tiff stack. The center of the 3D mask, its volume and elongation was then quantified using “3D ImageJ Suite” [85] in Fiji. A vertical reslice of the spheroid image in a plane going through the center of the spheroid and the center of the DNA‐HMP was used to locate the coordinates of the local edge of the spheroid. The normalized radial position of each HMP was then calculated as the distance between the HMP center and the spheroid, divided by the local distance between the spheroid edge and its center.

The normal stress distribution on the surface of each DNA‐HMP can be decomposed into a hydrostatic component leading to a volume change [11], and a deviatoric component leading to a departure from the spherical shape [72]. The hydrostatic component is calculated from a reference configuration: When the initial size of the HMP is known, it is used as a reference configuration and the absolute hydrostatic stress is computed; when the initial size of the HMP is unknown, its size at the initial imaging time is used and thus a relative hydrostatic stress is computed. The deviatory component of the stress distribution and a total force on each DNA‐HMP, as an integral of that deviatory stress distribution, were computed using BeadBuddy.

### 4.28 Statistical Analysis

All data apart from RT‐DC data is presented showing the mean ± standard deviation of the given populations. When showing data based on triplicate measurements, the mean ± standard deviation correspond to their error propagated values. RT‐DC data is presented as the mean ± the standard error of the mean to account for the large sample sizes. In addition, the sample size is given for all depicted data, showing individual data points were possible. If not stated otherwise, statistical analysis was conducted using unpaired, two‐tailed Student's t‐tests and a minimum p‐value of 0.05 used to determine the statistical significance of the data. OriginPro 2021 ‐ Update 6 (Origin Lab Corporation) was used to conduct the statistical tests and to prepare the presented graphs for all data other than RT‐DC data. For long‐term stability analysis of DNA‐HMPs a paired Student's t‐test was used to determine the statistical significance as the data corresponds to the same sample at different time points. For RT‐DC data, statistical significance was analyzed using a linear mixed model (R‐lme4) as integrated in Shape‐Out (version 2.10.0 [80]) yielding ANOVA p‐values.

## Conflicts of Interest

The authors declare no conflicts of interest.

## Supporting information


**Supporting File 1**: adma73405‐sup‐0001‐SuppMat.pdf.

**Supporting File 2**: adma73405‐sup‐0001‐VideoS1‐S10.zip.

## Data Availability

The data that support the findings of this study are openly available via PUBLISSO ‐ Repository for Life Sciences (FRL) at https://doi.org/10.4126/FRL01‐006528199.
