# Supplementary material for: Mechanically Programmable DNA Hydrogel Microparticles for 3D Cellular Systems
Source: Adv Mater. 2026 May 31;38(37):e14218. doi: 10.1002/adma.202514218 (PMC13327248; doi:10.1002/adma.202514218)
Supplement: Supplementary file 1 — Supporting File 1: adma73405‐sup‐0001‐SuppMat.pdf. [file ADMA-38-e14218-s002.pdf]

Supporting Information: Mechanically Programmable DNA Hydrogel Microparticles for 3D Cellular Systems

Tobias Walther, Eleni Dalaka, Gotthold Fläschner, Manuel Gómez-González, Ilia Platzman, Sadaf Pashapour, Michelle Emmert, Pere Roca-Cusachs, Xavier Trepat, Kerstin Göpfrich**

Tobias Walther, Michelle Emmert, Prof. Dr. Kerstin Göpfrich
Address: Heidelberg University, Center for Molecular Biology of Heidelberg University (ZMBH), Biophysical Engineering Group, Berliner Straße 53, 69120 Heidelberg, Germany
E-mail Address: k.goepfrich@zmbh.uni-heidelberg.de

Dr. Eleni Dalaka, Dr. Manuel Gómez-González, Prof. Dr. Xavier Trepat
Address: Institute for Bioengineering of Catalonia (IBEC), Integrative cell and tissue dynamics, Baldiri Reixac 15-21, 08028 Barcelona
E-mail Address: xtrepat@ibecbarcelona.eu

Dr. Gotthold Fläschner, Prof. Dr. Pere Roca-Cusachs
Address: Institute for Bioengineering of Catalonia (IBEC), Cellular and molecular mechanobiology, Baldiri Reixac 15-21, 08028 Barcelona

Dr. Sadaf Pashapour
Address: Heidelberg University, Institute for Molecular Systems Engineering and Advanced Materials (IMSEAM), Heidelberg University
Microfabrication and Microfluidics Core Facility (µFluCF), Institute for Molecular Systems Engineering and Advanced Materials (IMSEAM), Heidelberg University, INF 225, 69120 Heidelberg

Dr. Ilia Platzman
Address: Max Planck Institute for Medical Research, Department of Cellular Biophysics, Jahnstraße 29, 69120 Heidelberg, Germany

Contents

1 Supporting Tables **4**

1.1 Table S1: List of DNA sequences 4

2 Supporting Figures **5**

2.1 Figure S1: Designs of microfluidic devices used to create size-controlled DNA-HMPs 5

2.2 Figure S2: Time-dependent formation of DNA-HMPs 6

2.3 Figure S3: Linker-dependent formation of DNA-HMPs 7

2.4 Figure S4: Droplet-templated formation of 3-arm short DNA-HMPs over time 8

2.5 Figure S5: Fluorescence recovery after photobleaching (FRAP) on 3-arm short DNA-HMPs 9

2.6 Figure S6: Fluorescence recovery after photobleaching (FRAP) on 3-arm short DNA-HMPs with intercalating Hoechst and free nanostars 10

2.7 Figure S7: Aspect ratio of 3-arm short DNA-HMPs 11

2.8 Figure S8: Analysis of long-term stability of 3-arm short DNA-HMPs 12

2.9 Figure S9: Melting curve and ΔG analysis of DNA linkers used in this study 13

2.10 Figure S10: ΔG analysis of DNA nanostars used in this study 14

2.11 Figure S11: Confocal fluorescence microscopy of DNA-HMPs created using 3-arm, 4-arm, 6-arm and 6-arm flexible DNA nanostars 15

2.12 Figure S12: Control of 6-arm DNA-HMP formation by DNA linker design 16

2.13 Figure S13: DNA-HMP stability during heating 17

2.14 Figure S14: Fluorescence recovery after photobleaching (FRAP) on 3-arm DNA-HMPs . . 18

2.15 Figure S15: Fluorescence recovery after photobleaching (FRAP) on 4-arm DNA-HMPs . . 19

2.16 Figure S16: Fluorescence recovery after photobleaching (FRAP) on 6-arm flexible DNA-HMPs 20

2.17 Figure S17: Fluorescence recovery after photobleaching (FRAP) on 6-arm DNA-HMPs . . 21

2.18 Figure S18: Controlling DNA-HMP size by water-in-oil droplet size 22

2.19 Figure S19: Binding of DNA-HMP to a glass substrate after poly-l-lysine functionalization following electrostatic interaction 23

2.20 Figure S20: Dynamic mechanical analysis of 3-arm short DNA-HMPs 24

2.21 Figure S21: Dynamic mechanical analysis of 3-arm DNA-HMPs 25

2.22 Figure S22: Dynamic mechanical analysis of 4-arm DNA-HMPs 26

2.23 Figure S23: Dynamic mechanical analysis of 6-arm flexible DNA-HMPs 27

2.24 Figure S24: Dynamic mechanical analysis of 6-arm DNA-HMPs 28

2.25 Figure S25: DNA-HMPs display different relaxation behaviors during microindentation . . 29

2.26 Figure S26: Deformation of DNA-HMPs during RT-DC 30

2.27 Figure S27: Real-time deformability cytometry of 3-arm short DNA-HMPs 31

2.28 Figure S28: Real-time deformability cytometry of 6-arm flexible DNA-HMPs 32

2.29 Figure S29: Dynamic real-time deformability cytometry of 3-arm short DNA-HMPs 33

2.30 Figure S30: Dynamic real-time deformability cytometry of 6-arm flexible DNA-HMPs . . . 34

2.31 Figure S31: Real-time deformability cytometry of 6-arm DNA-HMPs 35

2.32 Figure S32: Real-time deformability cytometry of 6-arm DNA-HMPs at 0.4 µL/s flow rate . 36

2.33 Figure S33: Polyacrylamide gel electrophoresis of modified and unmodified elongated linker, 6-arm linker and flexible 6-arm linker 37
2.34 Figure S34: Incorporation of 5-FAM-modified DNA linkers into 3-arm DNA-HMPs 38
2.35 Figure S35: Incorporation of 5-FAM-modified DNA linkers into 6-arm flexible and 6-arm DNA-HMPs 39
2.36 Figure S36: Analysis of 5-FAM-modified DNA linker uptake into DNA-HMPs 40
2.37 Figure S37: Analysis of DNA-HMP stability under cell culture conditions 41
2.38 Figure S38: DNA-HMP particle elongation and measured traction forces 42
2.39 Figure S39: DNA-HMP deformation as a function of radial position in fibroblast spheroids 43
2.40 Figure S40: Extraction of drag force amplitude via force time series 44
2.41 Figure S41: Correction of the loss modulus by the viscous drag contribution 45

3 Supporting Videos 46

3.1 Video S1: Droplet-templated formation of 3-arm short DNA-HMP over time 46
3.2 Video S2: Fluorescence recovery after photobleaching of released DNA-HMP 46
3.3 Video S3: Integration of 3-arm DNA-HMPs into 3D fibroblast spheroids 46
3.4 Video S4: Integration of 4-arm DNA-HMPs into 3D fibroblast spheroids 46
3.5 Video S5: Integration of 6-arm flexible DNA-HMPs into 3D fibroblast spheroids 46
3.6 Video S6: Integration of 6-arm DNA-HMPs into 3D fibroblast spheroids 46
3.7 Video S7: Deformation of a 3-arm DNA-HMP in a 3D fibroblast spheroid over time 46
3.8 Video S8: Deformation of a 4-arm DNA-HMP in a 3D fibroblast spheroid over time 47
3.9 Video S9: Deformation of a 6-arm flexible DNA-HMP in a 3D fibroblast spheroid over time 47
3.10 Video S10: Deformation of a 6-arm DNA-HMP in a 3D fibroblast spheroid over time 47

4 Supplementary Note 1: Correction for drag force 48

5 Supplementary Note 2: Estimation of c[RGD] ligand density on the surface of DNA-HMPs 49

1 Supporting Tables

1.1 Table S1: List of DNA sequences

Table S1: DNA sequences used in this study. The fluorescent label cyanine 3 is abbreviated as Cy3. Dibenzocyclooctyne is abbreviated as DBCO. Modifications are highlighted in italic.

Name	DNA sequence 5' - 3'
**3-arm short**	
A-1	GACCAACACCAGTGAGGACGGAAGTTTGTCGTAGCATCGCACC
A-2	GACCAACACCAACCACGCCTGTCCATTACTTCCGTCCTCACTG
A-3	GACCAACACGGTGCGATGCTACGACTTTGGACAGGCGTGGTTG
B-1	CAGTGAGGACGGAAGTTTGTCGTAGCATCGCACCCGACAGGAA
B-1-Cy3	*Cy3*-CAGTGAGGACGGAAGTTTGTCGTAGCATCGCACCCGACAGGAA
B-2	CAACCACGCCTGTCCATTACTTCCGTCCTCACTGCGACAGGAA
B-3	GGTGCGATGCTACGACTTTGGACAGGCGTGGTTGCGACAGGAA
Linker	GTGTTGGTCTTCCTGTCG
**3-arm**	
C-1	TGCGACCAACACCAGTGAGGACGGAAGTTTGTCGTAGCATCGCACC
C-2	TGCGACCAACACCAACCACGCCTGTCCATTACTTCCGTCCTCACTG
C-3	TGCGACCAACACGGTGCGATGCTACGACTTTGGACAGGCGTGGTTG
D-1	CAGTGAGGACGGAAGTTTGTCGTAGCATCGCACCACGCGACAGGAA
D-1-Cy3	*Cy3*-CAGTGAGGACGGAAGTTTGTCGTAGCATCGCACCACGCGACAGGAA
D-1-390	*Atto390*-CAGTGAGGACGGAAGTTTGTCGTAGCATCGCACCACGCGACAGGAA
D-2	CAACCACGCCTGTCCATTACTTCCGTCCTCACTGACGCGACAGGAA
D-3	GGTGCGATGCTACGACTTTGGACAGGCGTGGTTGACGCGACAGGAA
Elongated Linker	GTGTTGGTCGCATTCCTGTCGCGT
Elongated Linker DBCO	*DBCO*-TGTGTTGGTCGCATTCCTGTCGCGT
**4-arm**	
E-1	CTACTATGGCGGGTGATAAATTCGGGAAGAGCATGCCCATCCACGCGACAGGAA
E-1-488	*Atto-488*-CTACTATGGCGGGTGATAAATTCGGGAAGAGCATGCCCATCCACGCGACAGGAA
E-2	GGATGGGCATGCTCTTCCCGTTCTCAACTGCCTGGTGATACGACGCGACAGGAA
E-3	CGTATCACCAGGCAGTTGAGTTCATGCGAGGGTCCAATACCGACGCGACAGGAA
E-4	CGGTATTGGACCCTCGCATGTTTTTATCACCCGCCATAGTAGACGCGACAGGAA
F-1	TGCGACCAACACCTACTATGGCGGGTGATAAATTCGGGAAGAGCATGCCCATCC
F-2	TGCGACCAACACGGATGGGCATGCTCTTCCCGTTCTCAACTGCCTGGTGATACG
F-3	TGCGACCAACACCGTATCACCAGGCAGTTGAGTTCATGCGAGGGTCCAATACCG
F-4	TGCGACCAACACCGGTATTGGACCCTCGCATGTTTTTATCACCCGCCATAGTAG
**6-arm/6-arm flexible**	
G-1	GCTGGACTAACGGAACGGTTAGTCAGGTATGCCAGCACATAGCTTCCTCG
G-1-647	*Atto-647N*-GCTGGACTAACGGAACGGTTAGTCAGGTATGCCAGCACATAGCTTCCTCG
G-2	CTCAGAGAGGTGACAGCATTCCGTTCCGTTAGTCCAGCATAGCTTCCTCG
G-3	CCATGGTCCCAAGTGATGTTTGCTGTCACCTCTCTGAGATAGCTTCCTCG
G-4	CGGCGCTGTAAATTTGCGTTCATCACTTGGGACCATGGATAGCTTCCTCG
G-5	CAGACGTCACTCTCCAACTTCGCAAATTTACAGCGCCGATAGCTTCCTCG
G-6	GTGCTGGCATACCTGACTTTGTTGGAGAGTGACGTCTGATAGCTTCCTCG
H-1	TGCGACCAACACGCTGGACTAACGGAACGGTTAGTCAGGTATGCCAGCAC
H-2	TGCGACCAACACCTCAGAGAGGTGACAGCATTCCGTTCCGTTAGTCCAGC
H-3	TGCGACCAACACCCATGGTCCCAAGTGATGTTTGCTGTCACCTCTCTGAG
H-4	TGCGACCAACACCGGCGCTGTAAATTTGCGTTCATCACTTGGGACCATGG
H-5	TGCGACCAACACCAGACGTCACTCTCCAACTTCGCAAATTTACAGCGCCG
H-6	TGCGACCAACACGTGCTGGCATACCTGACTTTGTTGGAGAGTGACGTCTG
6-arm-linker	GTGTTGGTCGCACGAGGAAGCTAT
Flexible 6-arm-linker	GTGTTGGTCGCATTCGAGGAAGCTAT
Flexible 6-arm-linker 1 T	GTGTTGGTCGCATCGAGGAAGCTAT
6-arm-linker DBCO	*DBCO*-TGTGTTGGTCGCACGAGGAAGCTAT
Flexible 6-arm-linker DBCO	*DBCO*-TGTGTTGGTCGCATTCGAGGAAGCTAT

2 Supporting Figures

2.1 Figure S1: Designs of microfluidic devices used to create size-controlled DNA-HMPs

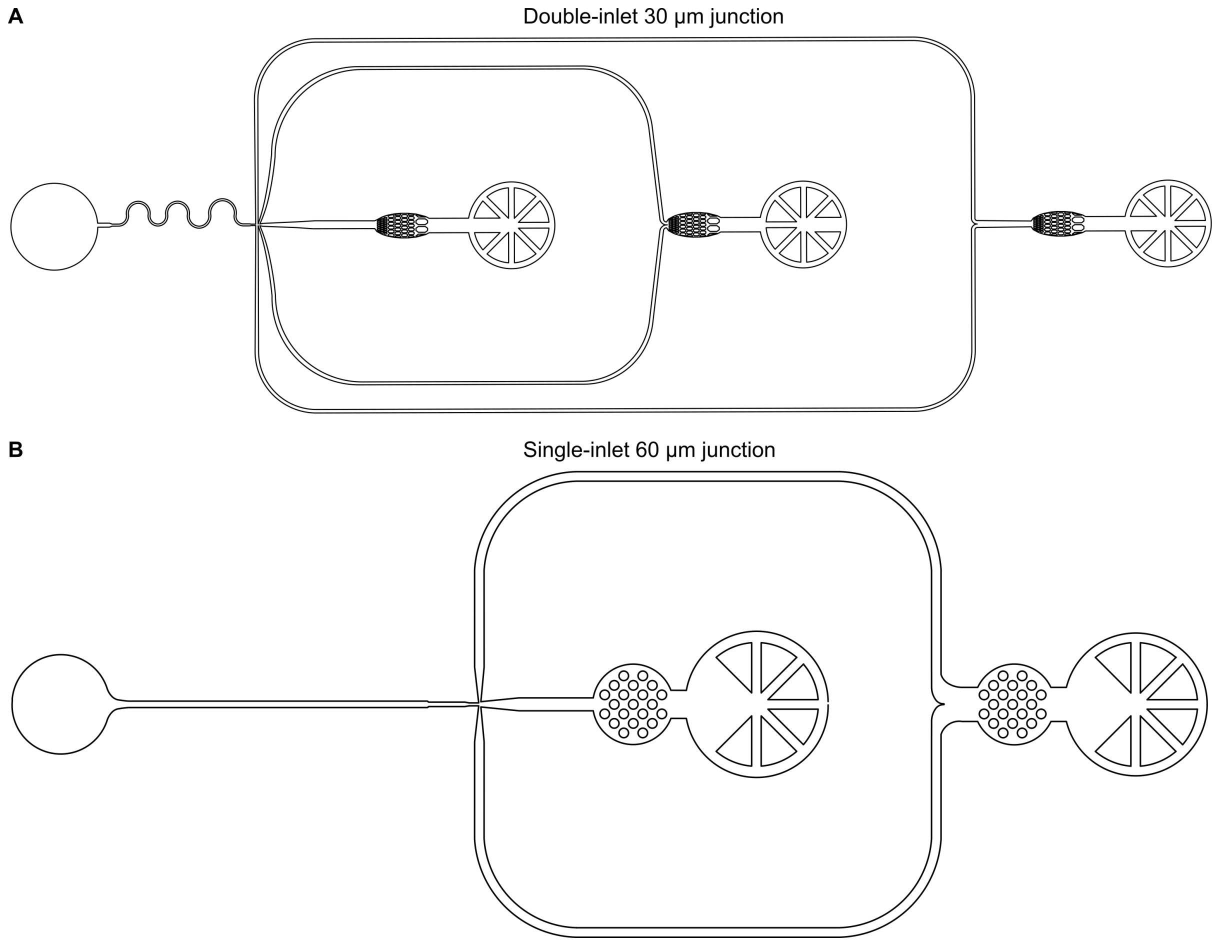

Figure S1: Microfluidics chips used for the preparation of size-controlled DNA-HMPs. A) 30 µm junction double-inlet device used to create DNA-HMPs of sizes up to 30 µm. B) 60 µm junction single-inlet device used to create DNA-HMPs of sizes up to 60 µm.

2.2 Figure S2: Time-dependent formation of DNA-HMPs

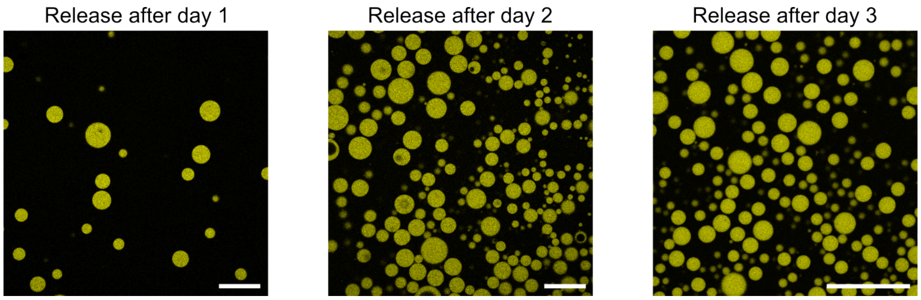

Figure S2: Control experiments of DNA-HMP formation. Confocal fluorescence microscopy ($\lambda_{ex} = 561\,\text{nm}$, Cy3-labeled DNA, yellow) images of 3-arm short DNA-HMPs released after 1, 2 and 3 days of incubation in water-in-oil droplets. The highest yield of intact DNA-HMPs was achieved after 3 day incubation.

2.3 Figure S3: Linker-dependent formation of DNA-HMPs

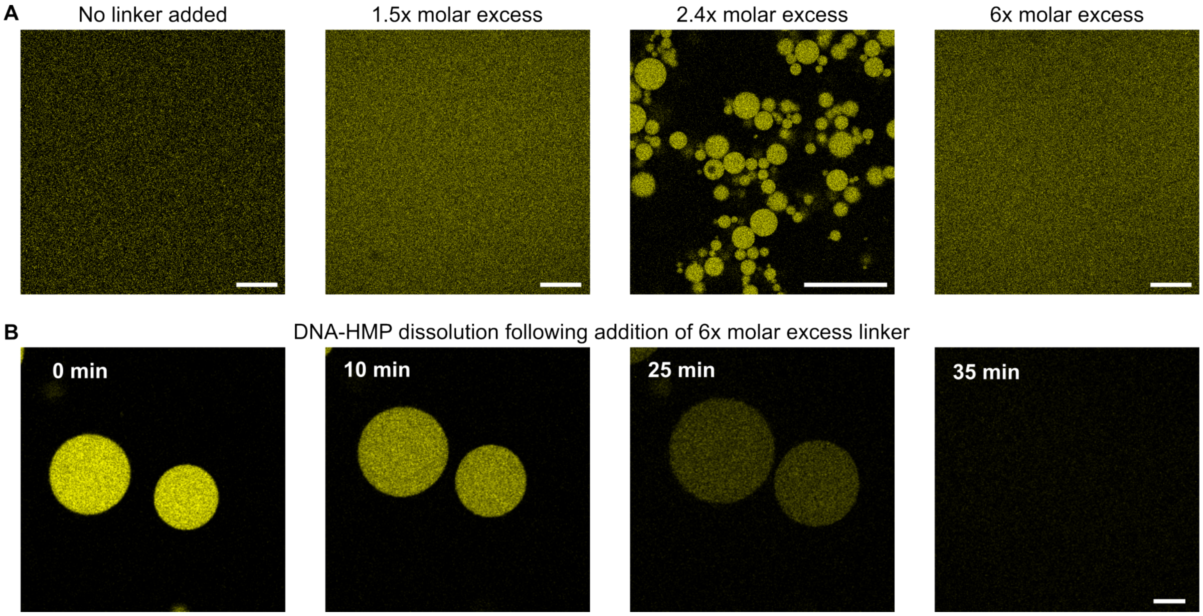

Figure S3: Effect of linker-to-nanostar ratio on DNA-HMP formation. A) Confocal fluorescence microscopy ($\lambda_{ex} = 561$ nm, Cy3-labeled DNA, yellow) showing no intact DNA-HMP release from water-in-oil droplets following incubation of the 3-arm nanostars with less than 2.4x molar excess of linker to the nanostars, as the available linkers result in insufficient connections between the nanostars. 3x molar excess is the optimal ratio where all three arms are bound. In turn, adding 6x molar excess of linker also results in no DNA-HMP formation as all monomers are saturated with linker molecules and no connections can be formed. Scale bars: 50 µm. B) Addition of 6x molar excess of linker to intact DNA-HMPs results in DNA-HMP dissolution over time due to strand displacement. Scale bar: 20 µm.

2.4 Figure S4: Droplet-templated formation of 3-arm short DNA-HMPs over time

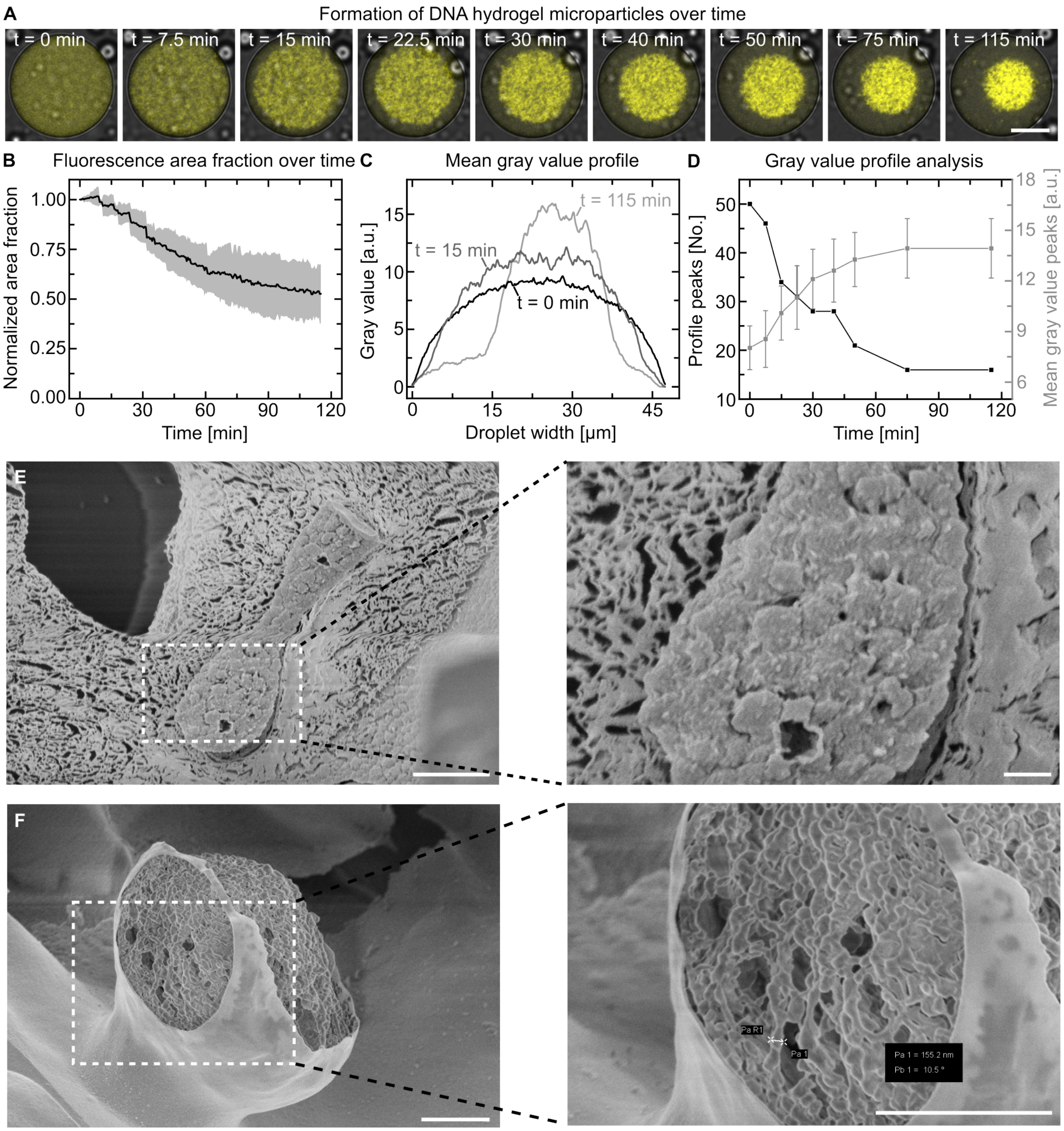

Figure S4: Formation of DNA-HMPs inside of water-in-oil droplets over time. A) Overlay of confocal fluorescence microscopy ($\lambda_{ex} = 561$ nm, Cy3-labeled DNA, yellow) and brightfield images of a 3-arm short DNA-HMP forming inside a water-in-oil droplet over the course of 115 min, showing how the DNA condensates simultaneously across the whole volume of the water-in-oil droplet to form a singular DNA-HMP. Scale bar: 20 µm. B) Measured fluorescence area fraction of the DNA signal inside of water-in-oil droplets over time. The mean measured area fraction of fluorescent pixels over all pixels within the acquired droplet images is shown, depicting a decrease in area until the resulting DNA-HMP is formed (n = 3, mean ± standard deviation). C) Exemplary gray value profiles from three time points of the images shown in A, showing an increase in fluorescence intensity over time as well as a narrowing of the distribution indicating concentration of signal. D) Analysis of all gray value profiles of the images shown in A. Over time, the number of fluorescence peaks measured across the droplet decreases (black curve, total number of detected curve peaks at each time point), while the mean gray value of the fluorescence peaks increases (light gray curve, mean ± standard deviation of each measured time point). Both plateau towards the end of the particle formation, depicting the condensation of the DNA across the droplet towards its center over time. E) Cross-section of a 3-arm short DNA-HMP at 20 µM DNA concentration as seen via cryoSEM imaging. In the center and on the right-hand side of the image, uncut regions of the network are visible showing phase-separated droplets of DNA of several nanometers in size. Scale bar: 1 µm. The zoom shows the phase-separated region in the center of the droplet in more detail. Scale bar: 200 nm. F) Further cryoSEM images of intact DNA-HMPs. Overview shows full DNA-HMP, while zoom shows close-up of the highlighted region with size measurement of the nano-doplets. Scale bars: 2 µm.

2.5 Figure S5: Fluorescence recovery after photobleaching (FRAP) on 3-arm short DNA-HMPs

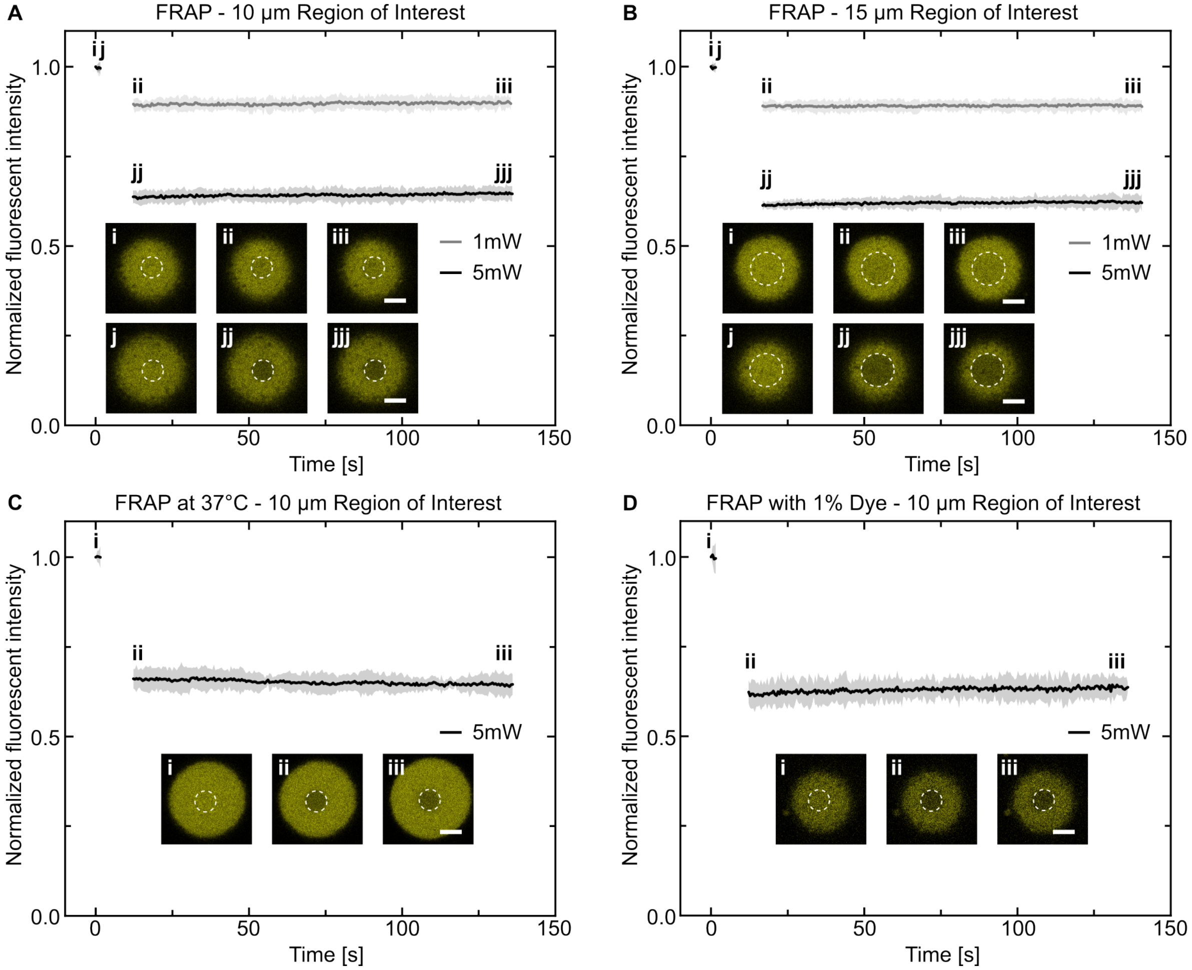

Figure S5: Fluorescence recovery after photobleaching (FRAP) on 3-arm short DNA-HMPs. A) FRAP using 1 mW and 5 mW of laser power at 10 µm ROI size and 10% dye concentration. Inlays show confocal fluorescence microscopy images ($\lambda_{ex} = 561$ nm, Cy3-labeled DNA, yellow) of DNA-HMPs before (*i* (1 mW), *j* (5 mW)) and after (*ii*, *iii* (1 mW), *jj*, *jjj* (5 mW)) bleaching. Scale bars: 10 µm. B) FRAP using 1 mW and 5 mW of laser power at 15 µm ROI size and 10% dye concentration. Inlays show confocal fluorescence microscopy images of DNA-HMPs before (*i* (1 mW), *j* (5 mW)) and after (*ii*, *iii* (1 mW), *jj*, *jjj* (5 mW)) bleaching. Scale bars: 10 µm. C) FRAP using 5 mW of laser power at 10 µm ROI size, 37°C and 10% dye concentration. Inlays show confocal fluorescence microscopy images of DNA-HMPs before (*i*) and after (*ii*, *iii*) bleaching. Scale bar: 10 µm. D) FRAP experiments using 5 mW of laser power at 10 µm ROI size and 1% dye concentration. Inlays show confocal fluorescence microscopy images of DNA-HMPs before (*i*) and after (*ii*, *iii*) bleaching. Scale bar: 10 µm. Data is shown as combined mean ± error propagated standard deviation of triplicate measurements measuring three individual DNA-HMPs per replicate.

2.6 Figure S6: Fluorescence recovery after photobleaching (FRAP) on 3-arm short DNA-HMPs with intercalating Hoechst and free nanostars

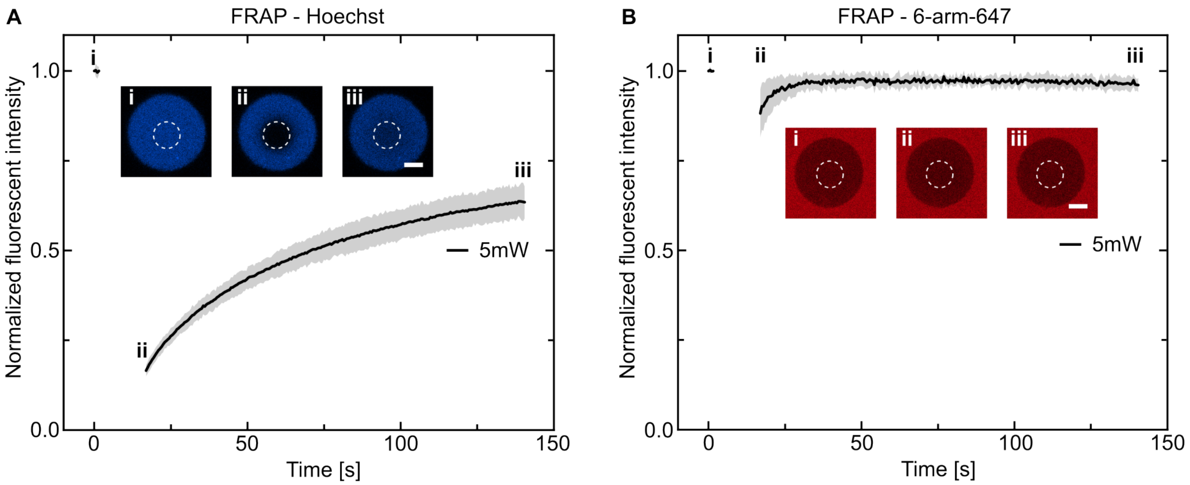

Figure S6: Fluorescence recovery after photobleaching (FRAP) on 3-arm short DNA-HMPs using either intercalating Hoechst dye or free ATTO-647N-labeled non-interacting 6-arm nanostars. A) FRAP using 5 mW of laser power at 15 µm ROI size and 20 mg/L Hoechst concentration. Inserts show confocal fluorescence microscopy images (λ_{ex} = 405 nm, intercalating Hoechst dye, blue) of DNA-HMPs before (*i*) and after (*ii*, *iii*) bleaching. Scale bar: 10 µm. The fluorescent signal of the intercalating Hoechst dye recovers up to 60% of the initial intensity within the observed time-frame, due to dynamic exchange of the bound and bleached Hoechst dyes intercalated in the DNA double helices with free Hoechst molecules from the surrounding solution. B) FRAP of free ATTO-647N-labeled 6-arm nanostar at 1 µM concentration using 5 mW of laser power at 15 µm ROI size. Inserts show confocal fluorescence microscopy images (λ_{ex} = 640 nm, ATTO-647N-labeled 6-arm nanostar, red) of DNA-HMPs before (*i*) and after (*ii*, *iii*) bleaching. Scale bar: 10 µm. The fluorescent signal of the free 6-arm nanostars recovers swiftly and almost entirely following bleaching, due to the free diffusion of the 6-arm nanostars in solution through the DNA-HMP network. As the sticky-end overhangs of the 3-arm short design and the 6-arm G nanostar used here are orthogonal and of different lengths, the nanostars only diffuse through the network without showing binding. The low degree of bleaching likewise stems from the fast diffusion of the nanostars. Data is shown as combined mean ± error propagated standard deviation of triplicate measurements measuring three individual DNA-HMPs per replicate.

2.7 Figure S7: Aspect ratio of 3-arm short DNA-HMPs

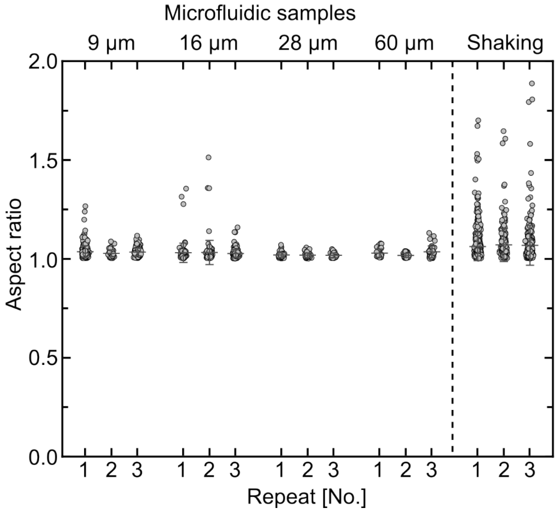

Figure S7: Aspect ratio distributions of 3-arm short DNA-HMPs presented in Figure 1C. The aspect ratios of the DNA-HMPs prepared via microfluidics and shaking method respectively are depicted in triplicate measurements. The data are depicted as individual data points as well as mean ± standard deviation for each sample.

2.8 Figure S8: Analysis of long-term stability of 3-arm short DNA-HMPs

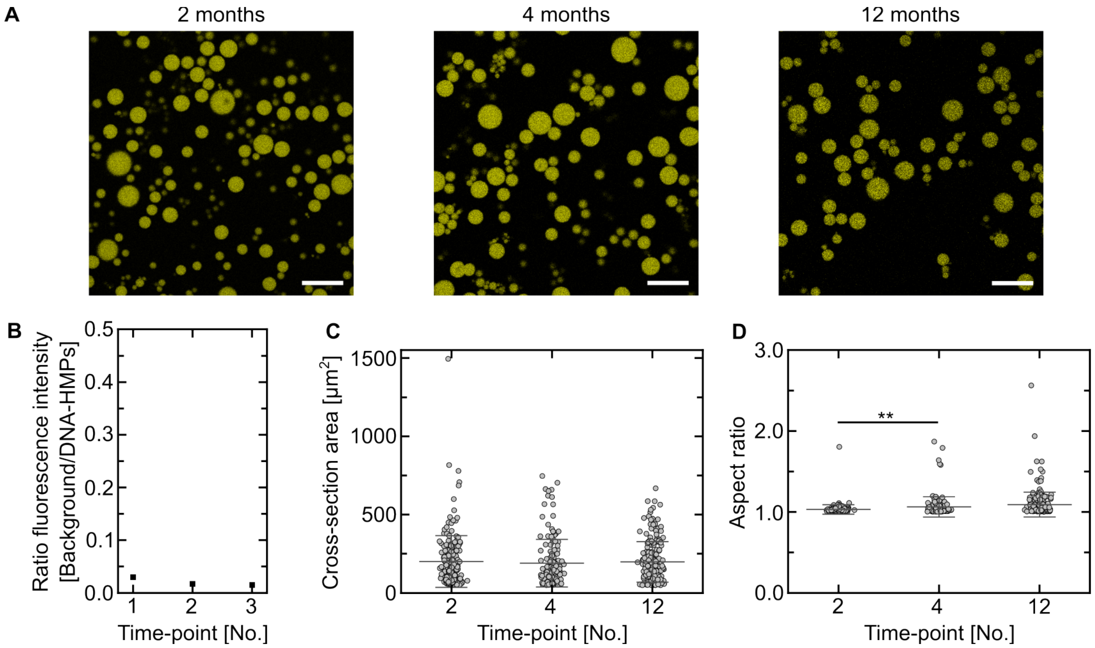

Figure S8: Long-term DNA-HMP stability. A) Confocal fluorescence microscopy ($\lambda_{ex} = 561\,\text{nm}$, Cy3-labeled DNA, yellow) showing intact DNA-HMPs after 2, 4 and 12 months of storage at 4°C. Scale bars: 50 µm. B) Ratio of background fluorescence over DNA-HMP fluorescence at three time points (1 = 2 months, 2 = 4 months, 3 = 12 months). The background fluorescence does not increase even after long storage indicating that the droplets do not break down and strands do not dissociate from them. C) Cross-section area distributions, as well as mean ± standard deviations of the DNA-HMPs observed at different time points over a 12 months time period. D) DNA-HMP aspect ratio distributions, as well as mean ± standard deviations of the DNA-HMPs observed over a 12 months time-period. **p-value: 0.009.

2.9 Figure S9: Melting curve and ΔG analysis of DNA linkers used in this study

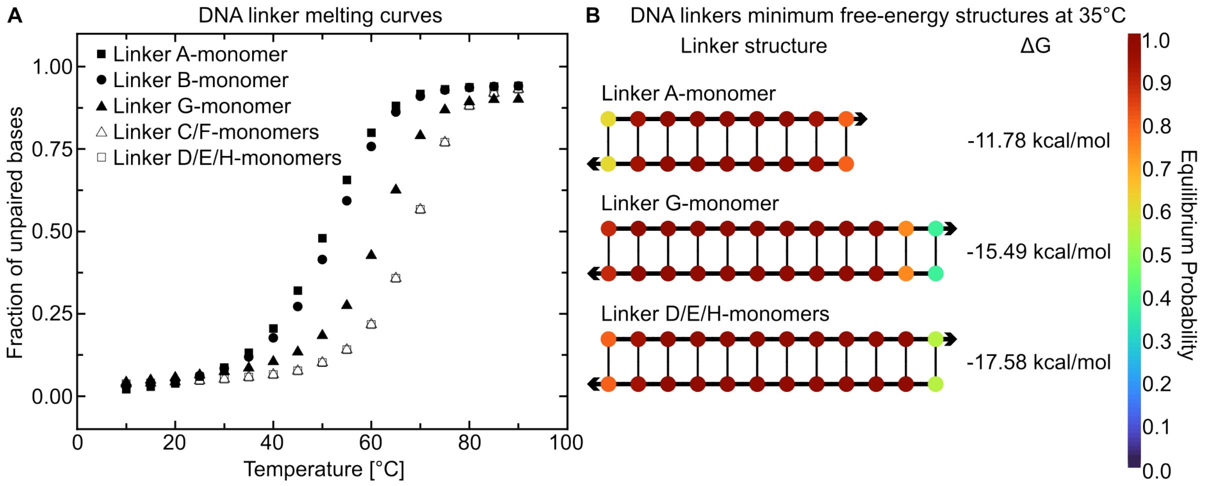

Figure S9: Melting curve and ΔG analysis of DNA linkers at 35°C as extracted from NuPack. A) Melting curves of the different linkers used in this study. B) Minimum free-energy structures and ΔG values of the different linker pairs. Note that for the elongated linker and the DNA linker only one half is shown as a representative structure.

2.10 Figure S10: ΔG analysis of DNA nanostars used in this study

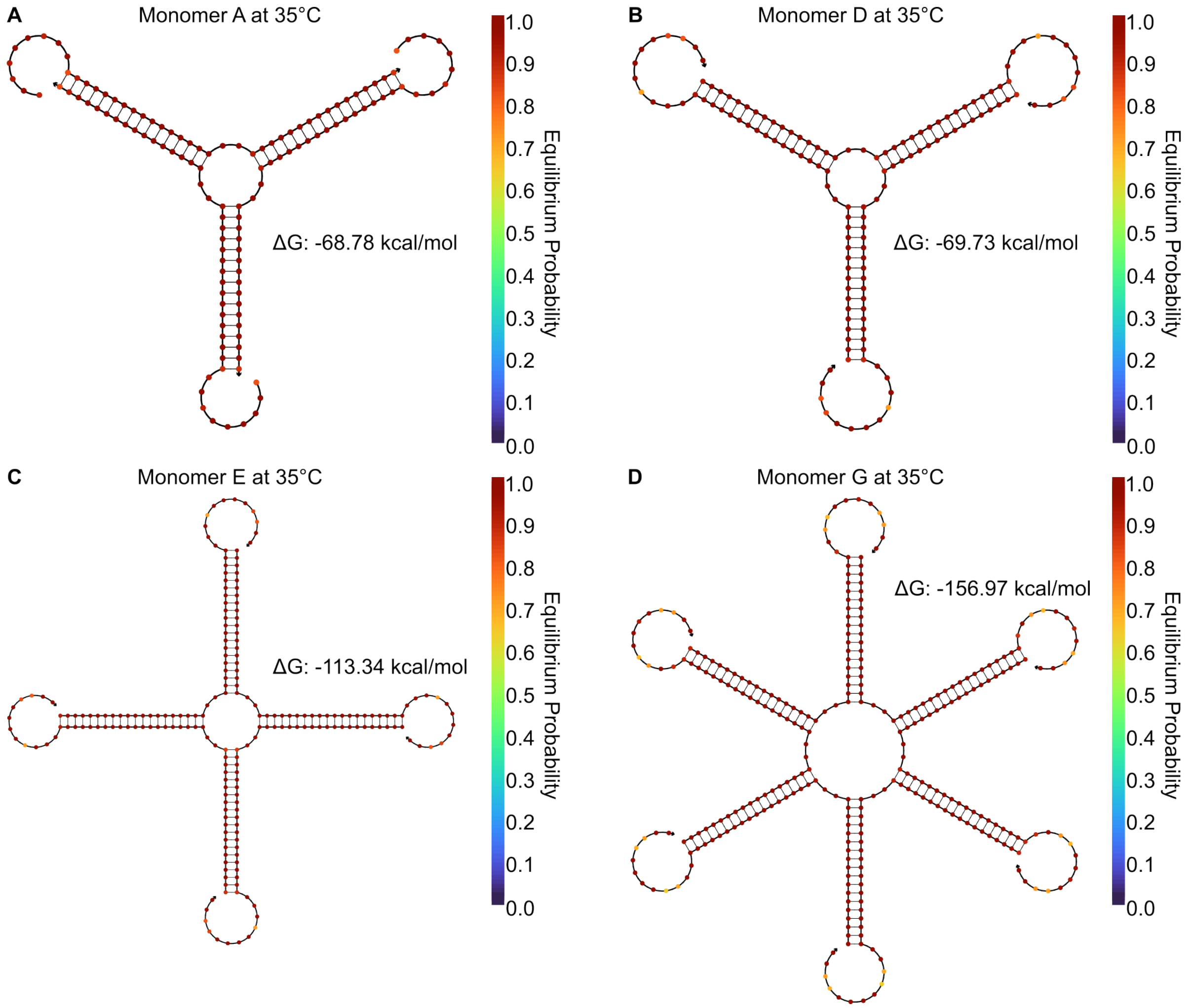

Figure S10: ΔG analysis of DNA nanostars at 35°C as extracted from NuPack. A-D) Minimum free-energy structures of the DNA nanostars used in this study at 35°C. Per design, one of the two nanostars is shown as a representative structures.

2.11 Figure S11: Confocal fluorescence microscopy of DNA-HMPs created using 3-arm, 4-arm, 6-arm and 6-arm flexible DNA nanostars

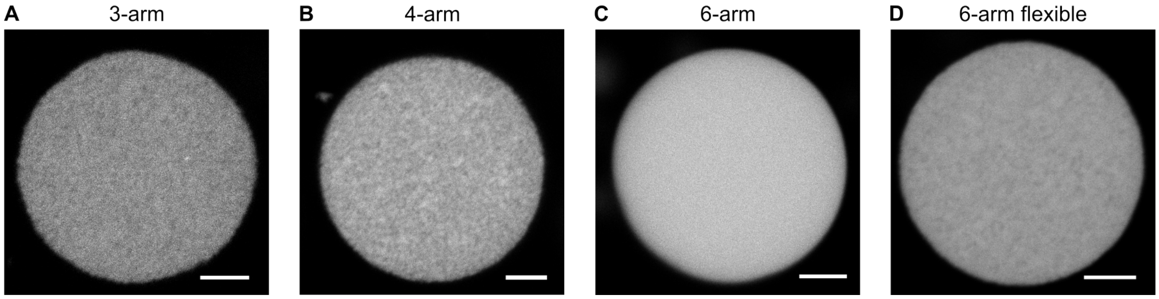

Figure S11: Confocal fluorescence microscopy data showing DNA-HMPs created using 3-arm, 4-arm, 6-arm and 6-arm flexible nanostars. A) Confocal fluorescence microscopy ($\lambda_{ex} = 561$ nm, Cy3-labeled DNA) of a 3-arm DNA-HMP. B) Confocal fluorescence microscopy ($\lambda_{ex} = 488$ nm, ATTO-488-labeled DNA) of a 4-arm DNA-HMP. C) Confocal fluorescence microscopy ($\lambda_{ex} = 640$ nm, ATTO-647N-labeled DNA) of a 6-arm DNA-HMP. D) Confocal fluorescence microscopy ($\lambda_{ex} = 640$ nm, ATTO-647N-labeled DNA) of a 6-arm flexible DNA-HMP. Scale bars: 10 µm.

2.12 Figure S12: Control of 6-arm DNA-HMP formation by DNA linker design

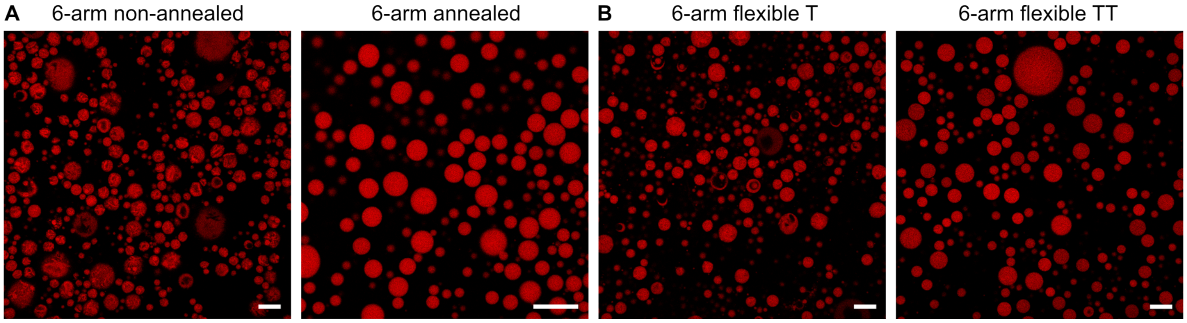

Figure S12: Stability of the 6-arm DNA-HMPs depending on linker design and annealing. A) Confocal fluorescence microscopy ($\lambda_{ex} = 640$ nm, ATTO-647N-labeled DNA, red) of 6-arm DNA-HMPs with the 6-arm linker and no annealing; and 6-arm linker following annealing. Non-annealed 6-arm DNA-HMPs are inhomogeneous in shape and brightness showing higher densities. Homogeneous 6-arm DNA-HMPs can be formed upon annealing. Scale bars: 50 µm. B) Confocal fluorescence microscopy ($\lambda_{ex} = 640$ nm, ATTO-647N-labeled DNA, red) of 6-arm DNA-HMPs using a flexible linker with one additional thymine and two additional thymines. Introducing flexibility into the center of the 6-arm linker improves homogeneity of the particles with two additional thymines giving the best results. Scale bars: 50 µm.

2.13 Figure S13: DNA-HMP stability during heating

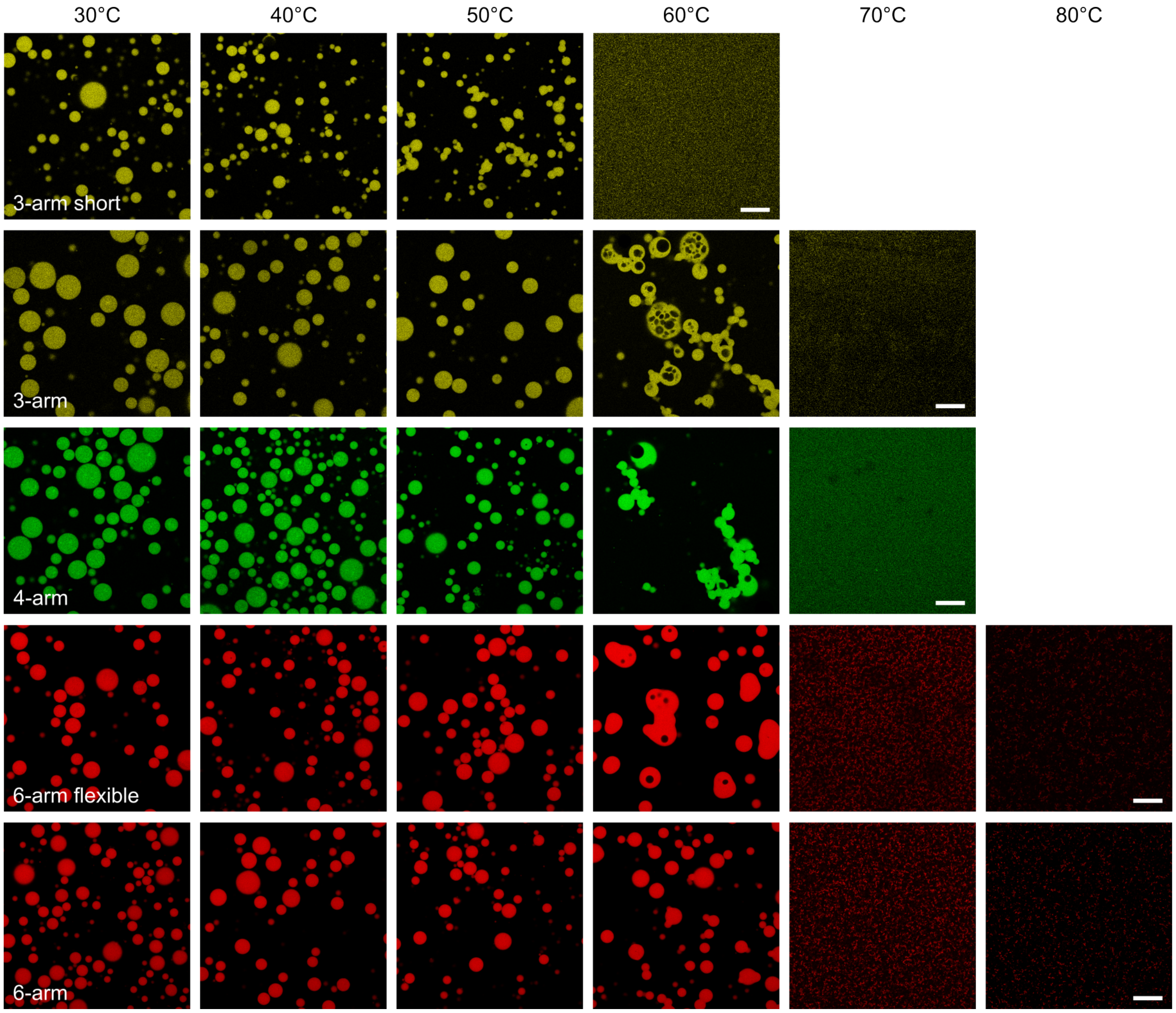

Figure S13: Thermal stability of DNA-HMPs. Confocal fluorescence microscopy (3-arm short/3-arm: $\lambda_{ex} = 561\,\text{nm}$, Cy3-labeled DNA, yellow; 4-arm: $\lambda_{ex} = 488\,\text{nm}$, ATTO-488-labeled DNA, green; 6-arm flexible/6-arm: $\lambda_{ex} = 640\,\text{nm}$, ATTO-647N-labeled DNA, red) of DNA-HMPs at increasing temperatures. All DNA-HMPs, apart from the 3-arm short design are stable up to 50°C showing fusion and eventual break-down at higher temperatures.

2.14 Figure S14: Fluorescence recovery after photobleaching (FRAP) on 3-arm DNA-HMPs

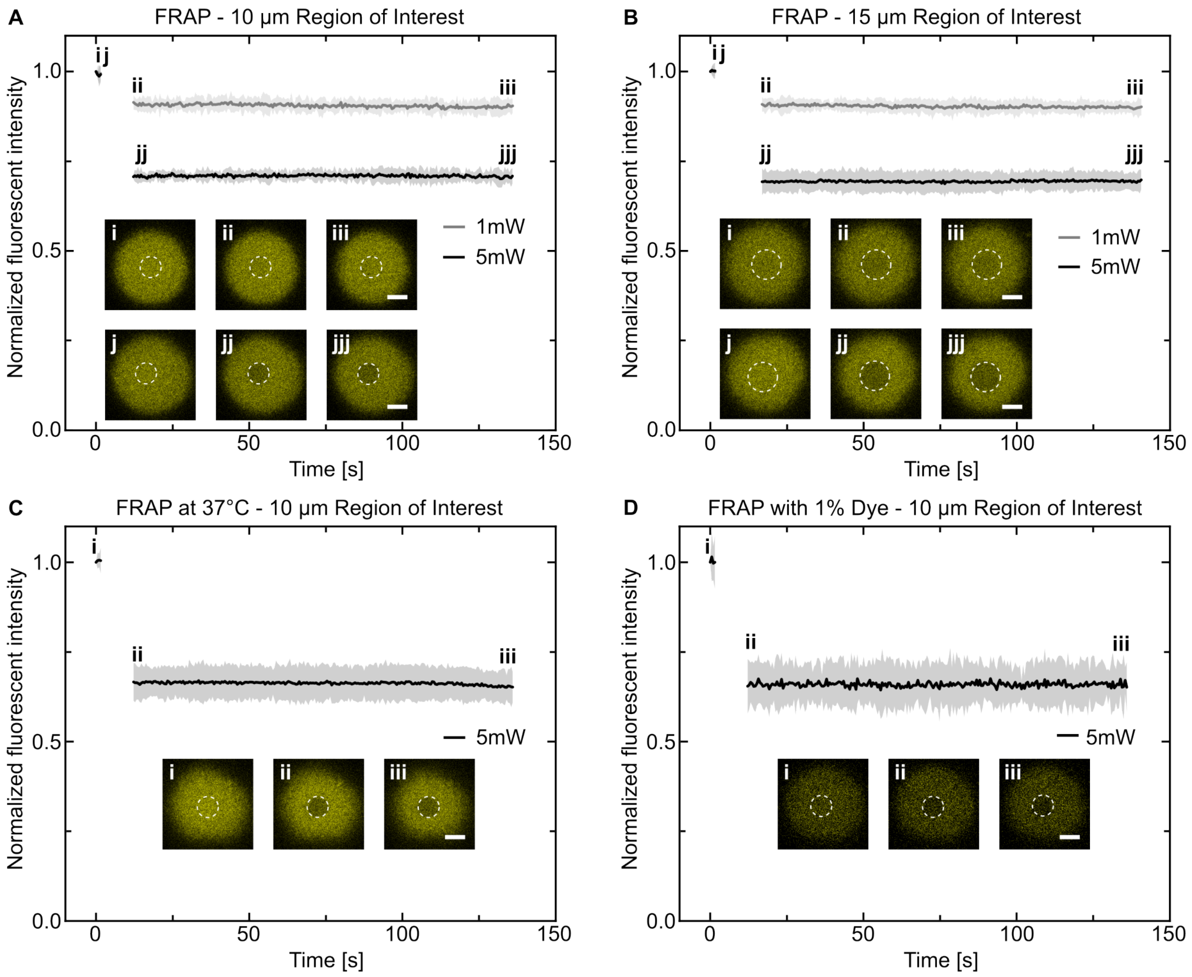

Figure S14: Fluorescence recovery after photobleaching (FRAP) on 3-arm DNA-HMPs. A) FRAP using 1 mW and 5 mW of laser power at 10 µm ROI size and 10% dye concentration. Inlays show confocal fluorescence microscopy images ($\lambda_{ex} = 561$ nm, Cy3-labeled DNA, yellow) of DNA-HMPs before (*i* (1 mW), *j* (5 mW)) and after (*ii*, *iii* (1 mW), *jj*, *jjj* (5 mW)) bleaching. Scale bars: 10 µm. B) FRAP using 1 mW and 5 mW of laser power at 15 µm ROI size and 10% dye concentration. Inlays show confocal fluorescence microscopy images of DNA-HMPs before (*i* (1 mW), *j* (5 mW)) and after (*ii*, *iii* (1 mW), *jj*, *jjj* (5 mW)) bleaching. Scale bars: 10 µm. C) FRAP using 5 mW of laser power at 10 µm ROI size, 37°C and 10% dye concentration. Inlays show confocal fluorescence microscopy images of DNA-HMPs before (*i*) and after (*ii*, *iii*) bleaching. Scale bar: 10 µm. D) FRAP using 5 mW of laser power at 10 µm ROI size and 1% dye concentration. Inlays show confocal fluorescence microscopy images of DNA-HMPs before (*i*) and after (*ii*, *iii*) bleaching. Scale bar: 10 µm. Data is shown as combined mean ± error propagated standard deviation of triplicate measurements measuring three individual DNA-HMPs per replicate.

2.15 Figure S15: Fluorescence recovery after photobleaching (FRAP) on 4-arm DNA-HMPs

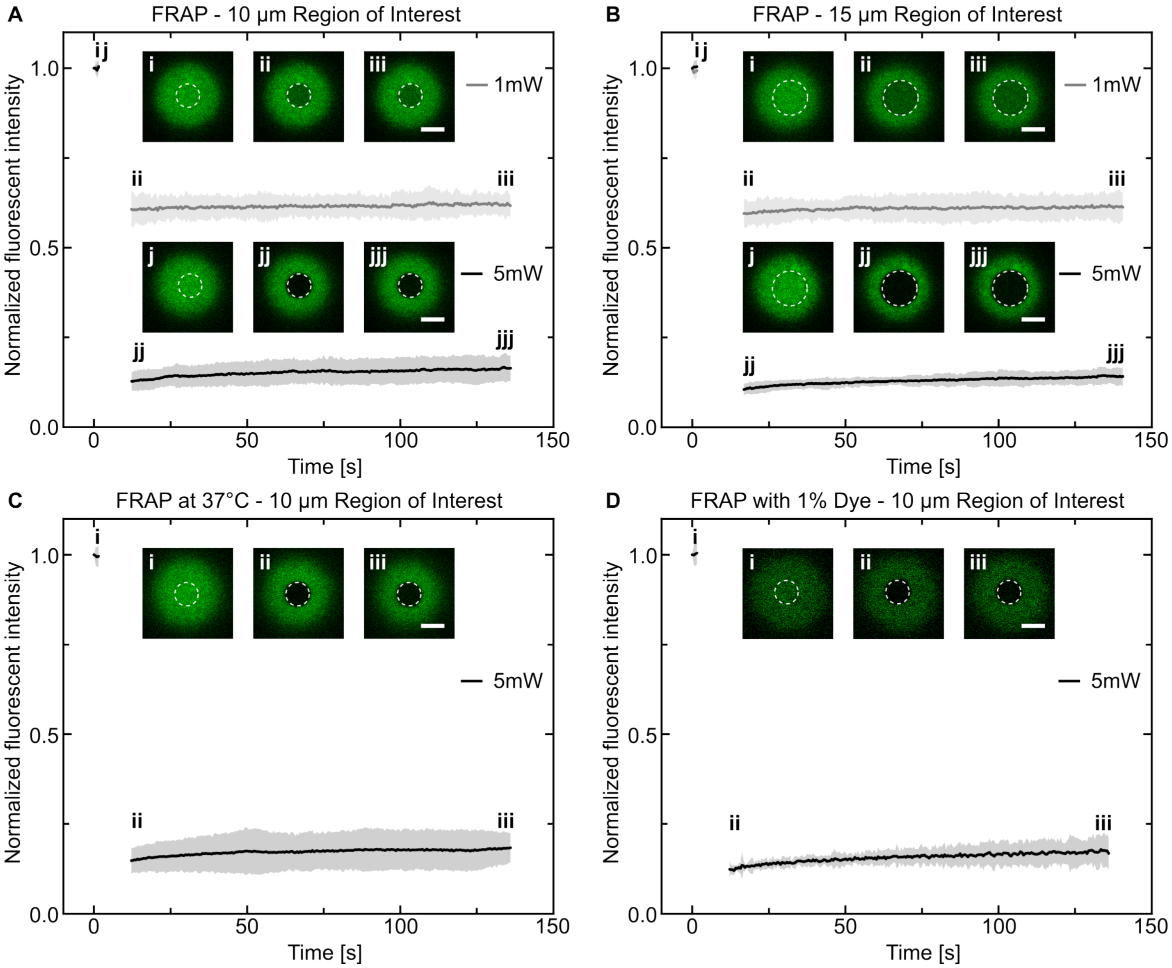

Figure S15: Fluorescence recovery after photobleaching (FRAP) on 4-arm DNA-HMPs. A) FRAP using 1 mW and 5 mW of laser power at 10 µm ROI size and 10% dye concentration. Inlays show confocal fluorescence microscopy images ($\lambda_{ex} = 488$ nm, Atto-488-labeled DNA, green) of DNA-HMPs before (*i* (1 mW), *j* (5 mW)) and after (*ii*, *iii* (1 mW), *jj*, *jjj* (5 mW)) bleaching. Scale bars: 10 µm. B) FRAP using 1 mW and 5 mW of laser power at 15 µm ROI size and 10% dye concentration. Inlays show confocal fluorescence microscopy images of DNA-HMPs before (*i* (1 mW), *j* (5 mW)) and after (*ii*, *iii* (1 mW), *jj*, *jjj* (5 mW)) bleaching. Scale bars: 10 µm. C) FRAP using 5 mW of laser power at 10 µm ROI size, 37°C and 10% dye concentration. Inlays show confocal fluorescence microscopy images of DNA-HMPs before (*i*) and after (*ii*, *iii*) bleaching. Scale bar: 10 µm. D) FRAP using 5 mW of laser power at 10 µm ROI size and 1% dye concentration. Inlays show confocal fluorescence microscopy images of DNA-HMPs before (*i*) and after (*ii*, *iii*) bleaching. Scale bar: 10 µm. Data is shown as combined mean ± error propagated standard deviation of triplicate measurements measuring three individual DNA-HMPs per replicate.

2.16 Figure S16: Fluorescence recovery after photobleaching (FRAP) on 6-arm flexible DNA-HMPs

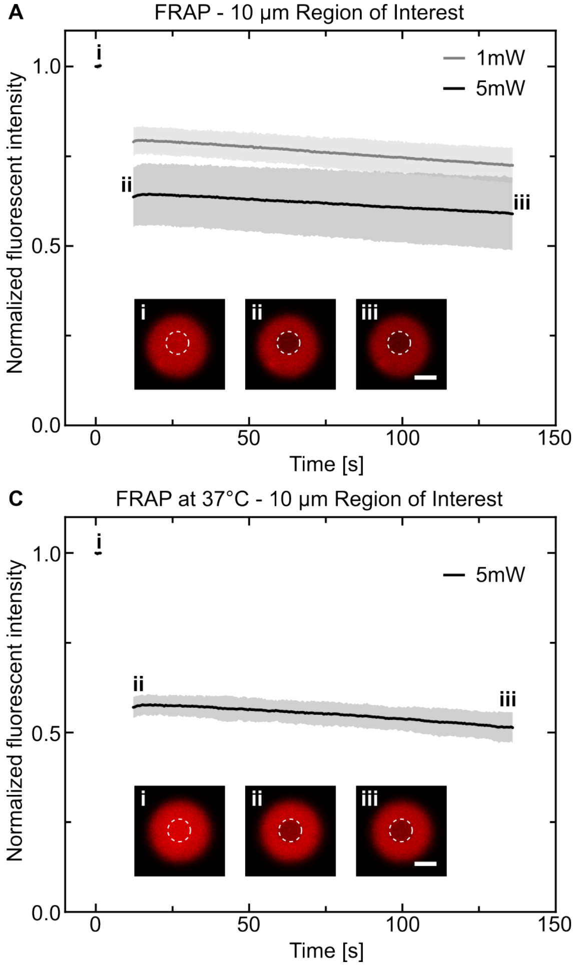

Figure S16: Fluorescence recovery after photobleaching (FRAP) on 6-arm flexible DNA-HMPs. A) FRAP using 1 mW and 5 mW of laser power at 10 µm ROI size and 1% dye concentration. Inlays show confocal fluorescence microscopy images (λ_{ex} = 640 nm, ATTO-647N-labeled DNA, red) of DNA-HMPs before (*i* (5 mW) and after (*ii*, *iii* (5 mW)) bleaching. Scale bar: 10 µm. B) FRAP using 1 mW and 5 mW of laser power at 15 µm ROI size and 1% dye concentration. Inlays show confocal fluorescence microscopy images of DNA-HMPs before (*i* (5 mW) and after (*ii*, *iii* (5 mW)) bleaching. Scale bar: 10 µm. C) FRAP using 5 mW of laser power at 10 µm ROI size, 37°C and 1% dye concentration. Inlays show confocal fluorescence microscopy images of DNA-HMPs before (*i* (5 mW) and after (*ii*, *iii* (5 mW)) bleaching. Scale bar: 10 µm. Data is shown as combined mean ± error propagated standard deviation of triplicate measurements measuring three individual DNA-HMPs per replicate.

2.17 Figure S17: Fluorescence recovery after photobleaching (FRAP) on 6-arm DNA-HMPs

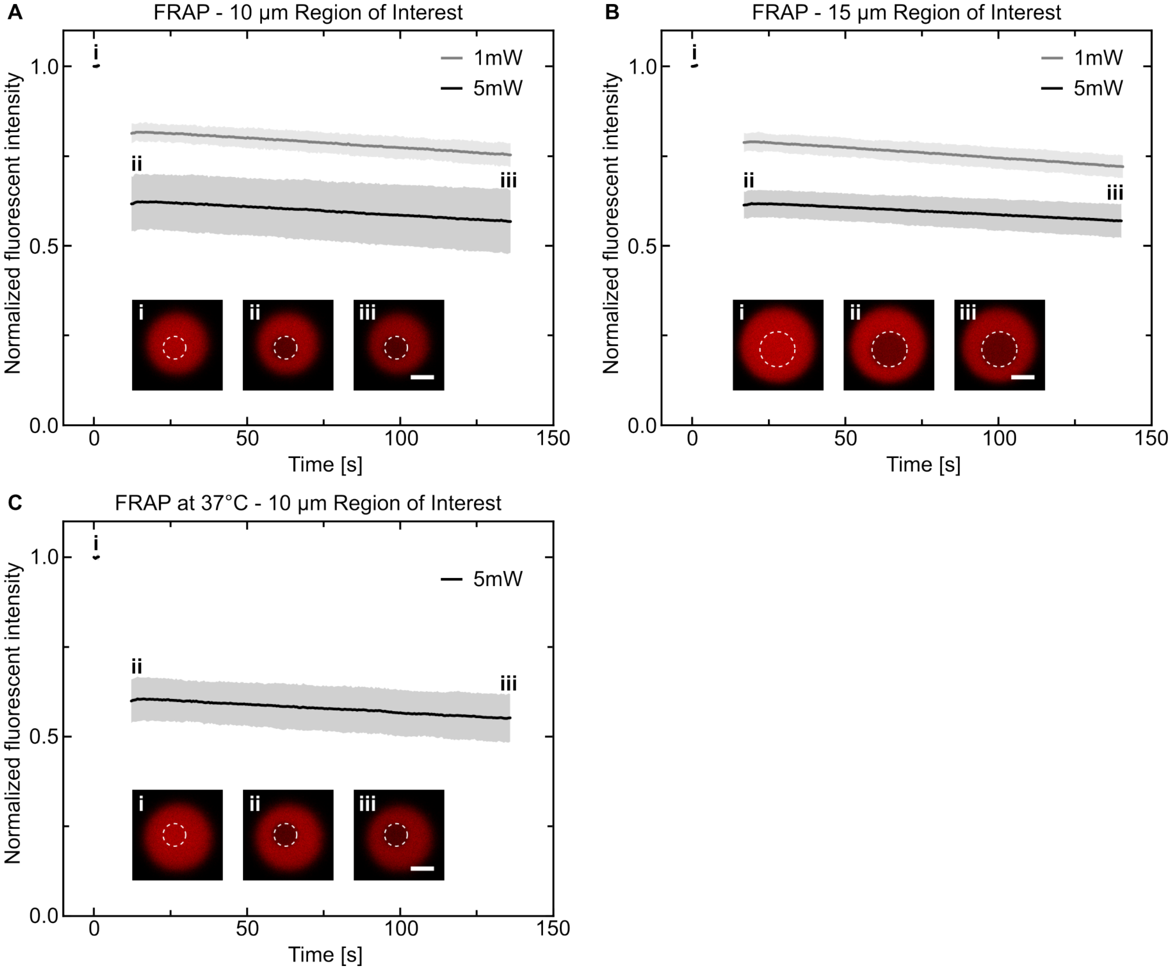

Figure S17: Fluorescence recovery after photobleaching (FRAP) on 6-arm DNA-HMPs. A) FRAP using 1 mW and 5 mW of laser power at 10 µm ROI size and 1% dye concentration. Inlays show confocal fluorescence microscopy images ($\lambda_{ex} = 640$ nm, ATTO-647N-labeled DNA, red) of DNA-HMPs before (*i* (5 mW) and after (*ii*, *iii* (5 mW)) bleaching. Scale bar: 10 µm. B) FRAP using 1 mW and 5 mW of laser power at 15 µm ROI size and 1% dye concentration. Inlays show confocal fluorescence microscopy images of DNA-HMPs before (*i* (5 mW) and after (*ii*, *iii* (5 mW)) bleaching. Scale bar: 10 µm. C) FRAP using 5 mW of laser power at 10 µm ROI size, 37°C and 1% dye concentration. Inlays show confocal fluorescence microscopy images of DNA-HMPs before (*i* (5 mW) and after (*ii*, *iii* (5 mW)) bleaching. Scale bar: 10 µm. Data is shown as combined mean ± error propagated standard deviation of triplicate measurements measuring three individual DNA-HMPs per replicate.

2.18 Figure S18: Controlling DNA-HMP size by water-in-oil droplet size

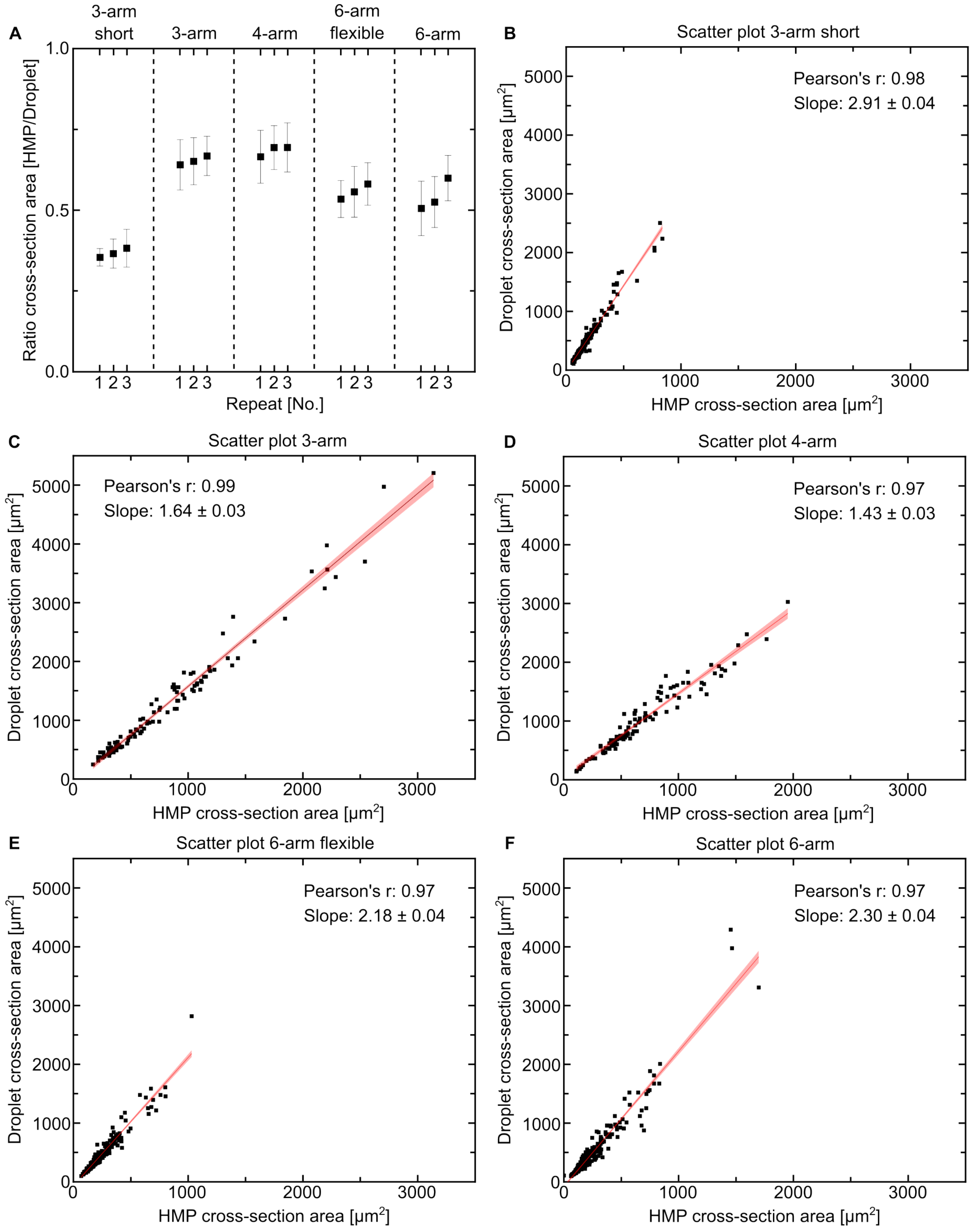

Figure S18: DNA-HMP condensation and size-control. A) Cross-section area of DNA-HMPs inside of water-in-oil droplets for all DNA nanostar designs. DNA-HMPs condense inside of water-in-oil droplets in a design-specific manner to 40-65% of the droplet size after formation. B-F) Scatter plots depicting DNA-HMP size over water-in-oil droplet size for all nanostar designs. Red lines correspond to linear fits of the data, red-shaded areas represent the 95% confidence intervals. Droplet size and HMP size are correlated.

2.19 Figure S19: Binding of DNA-HMP to a glass substrate after poly-l-lysine functionalization following electrostatic interaction

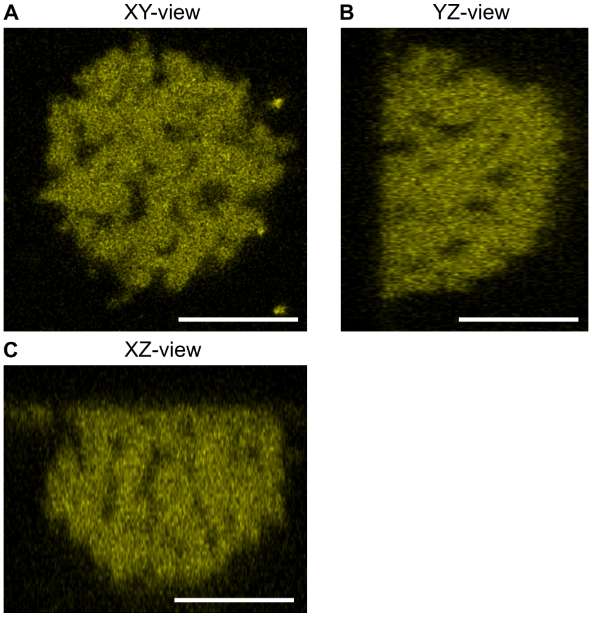

Figure S19: DNA-HMP bound to glass substrate following poly-l-lysine functionalization. A) Confocal fluorescence microscopy ($\lambda_{ex} = 561\,\text{nm}$, Cy3-labeled DNA, yellow) image of the xy-view of a 3-arm short DNA-HMP z-stack following electrostatic binding of the particle to a poly-l-lysine functionalized glass surface. B) Confocal fluorescence microscopy ($\lambda_{ex} = 561\,\text{nm}$, Cy3-labeled DNA, yellow) image of the yz-view of a 3-arm short DNA-HMP z-stack following electrostatic binding of the particle to a poly-l-lysine functionalized glass surface. C) Confocal fluorescence microscopy ($\lambda_{ex} = 561\,\text{nm}$, Cy3-labeled DNA, yellow) image of the xz-view of a 3-arm short DNA-HMP z-stack following electrostatic binding of the particle to a poly-l-lysine functionalized glass surface. Scale bars: 10 µm.

2.20 Figure S20: Dynamic mechanical analysis of 3-arm short DNA-HMPs

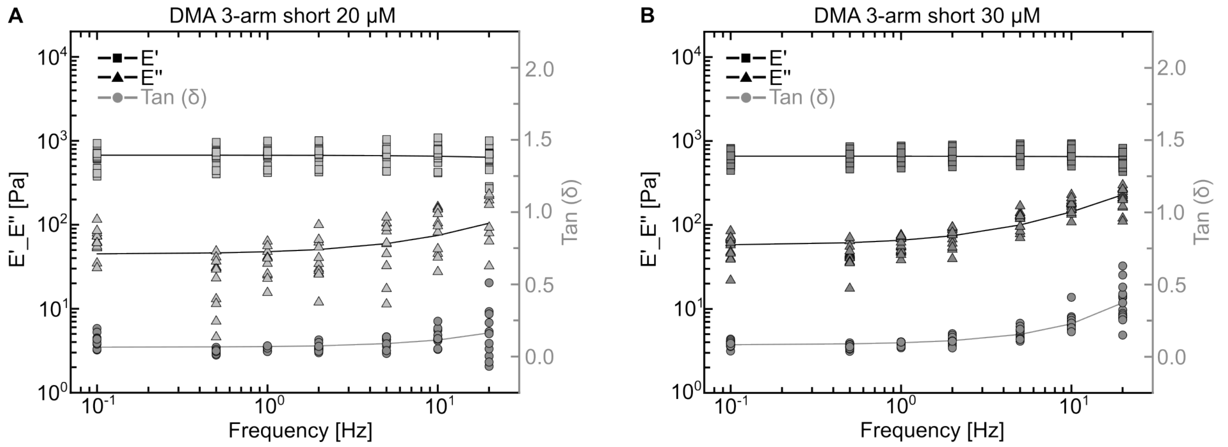

Figure S20: Further dynamic mechanical analysis (DMA) of 3-arm short DNA-HMPs. A) DMA plot of 3-arm short DNA-HMPs at 20 µM DNA concentration. The storage modulus E' (squares) and the loss modulus E" (triangles) are plotted on a logarithmic scale, tan (δ) (E"/E', filled circles) is plotted on a linear scale. Tan (δ) stays almost constant across all measured frequencies and only increases slightly at the highest measured frequency denoting the 3-arm short DNA-HMPs as behaving predominantly elastic. B) DMA plot of 3-arm short DNA-HMPs at 30 µM DNA concentration. The storage modulus E' (squares) and the loss modulus E" (triangles) are plotted on a logarithmic scale, tan (δ) (E"/E', filled circles) is plotted on a linear scale.

2.21 Figure S21: Dynamic mechanical analysis of 3-arm DNA-HMPs

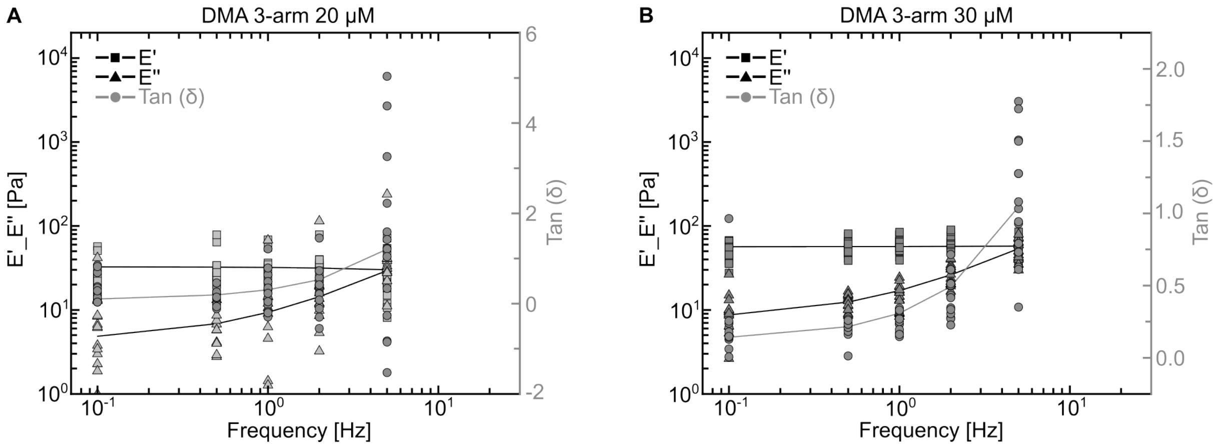

Figure S21: Further dynamic mechanical analysis (DMA) of 3-arm DNA-HMPs. A) DMA plot of 3-arm DNA-HMPs at 20 µM DNA concentration. The storage modulus E' (squares) and the loss modulus E" (triangles) are plotted on a logarithmic scale, tan (δ) (E"/E', filled circles) is plotted on a linear scale. Tan (δ) can be seen to increase more strongly already at lower frequencies showing clearly the cross-over point of E' and E" at 5 Hz, denoting the 3-arm DNA-HMPs as showing a more viscous response to higher mechanical stress than the 3-arm short DNA-HMPs. B) DMA plot of 3-arm DNA-HMPs at 30 µM DNA concentration. The storage modulus E' (squares) and the loss modulus E" (triangles) are plotted on a logarithmic scale, tan (δ) (E"/E', filled circles) is plotted on a linear scale.

2.22 Figure S22: Dynamic mechanical analysis of 4-arm DNA-HMPs

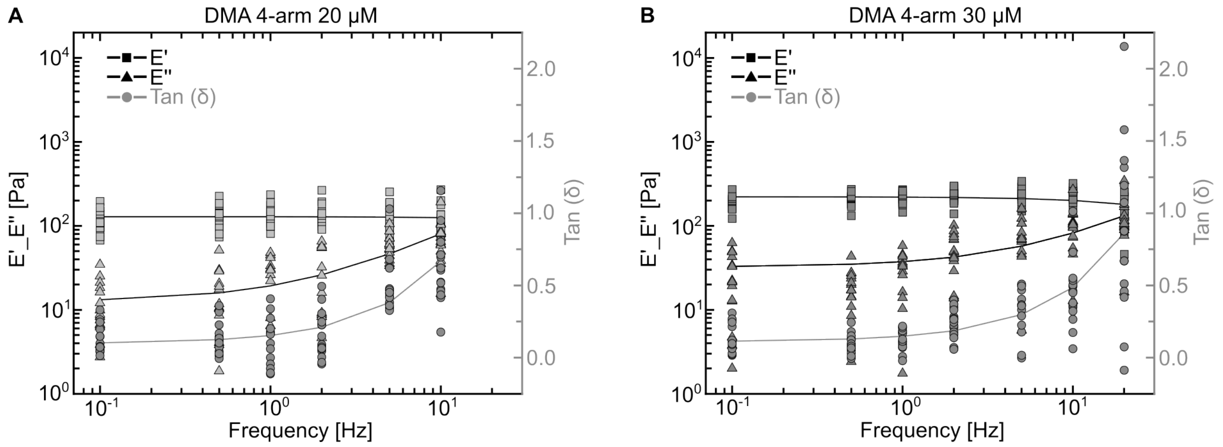

Figure S22: Further dynamic mechanical analysis (DMA) of 4-arm DNA-HMPs. A) DMA plot of 4-arm DNA-HMPs at 20 µM DNA concentration. The storage modulus E' (squares) and the loss modulus E" (triangles) are plotted on a logarithmic scale, tan (δ) (E"/E', filled circles) is plotted on a linear scale. Tan (δ) can be seen to increase more strongly already at lower frequencies showing clearly the cross-over point of E' and E" at 10 Hz, denoting the 4-arm DNA-HMPs as showing a more viscous response to higher mechanical stress than the 3-arm short DNA-HMPs, but less viscous than the 3-arm DNA-HMPs. B) DMA plot of 4-arm DNA-HMPs at 30 µM DNA concentration. The storage modulus E' (squares) and the loss modulus E" (triangles) are plotted on a logarithmic scale, tan (δ) (E"/E', filled circles) is plotted on a linear scale.

2.23 Figure S23: Dynamic mechanical analysis of 6-arm flexible DNA-HMPs

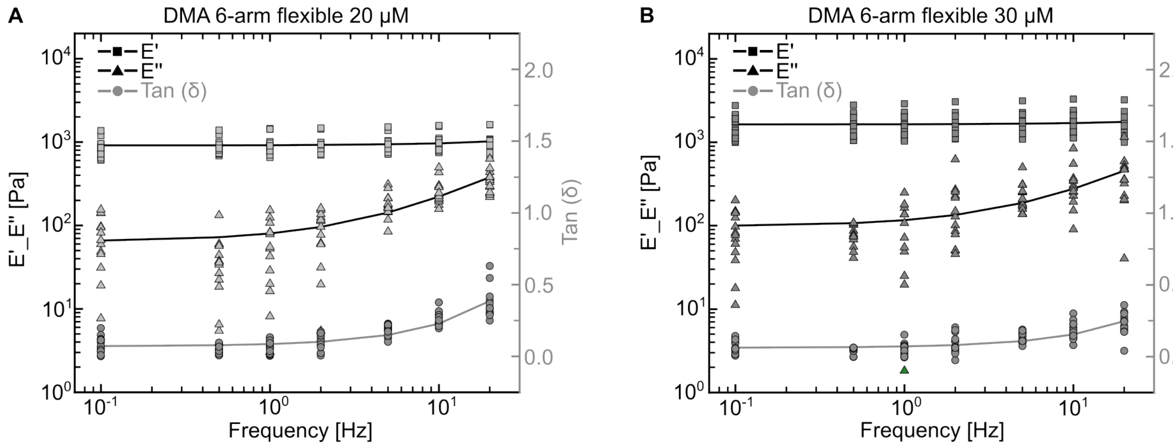

Figure S23: Further dynamic mechanical analysis (DMA) of 6-arm flexible DNA-HMPs. A) DMA plot of 6-arm flexible DNA-HMPs at 20 µM DNA concentration. The storage modulus E' (squares) and the loss modulus E" (triangles) are plotted on a logarithmic scale, tan (δ) (E"/E', filled circles) is plotted on a linear scale. Tan (δ) stays below 0.5 for all measured conditions with only a slight increase of E" at higher frequencies, denoting the 6-arm flexible DNA-HMPs as behaving mostly elastic. B) DMA plot of 6-arm flexible DNA-HMPs at 30 µM DNA concentration. The storage modulus E' (squares) and the loss modulus E" (triangles) are plotted on a logarithmic scale, tan (δ) (E"/E', filled circles) is plotted on a linear scale.

2.24 Figure S24: Dynamic mechanical analysis of 6-arm DNA-HMPs

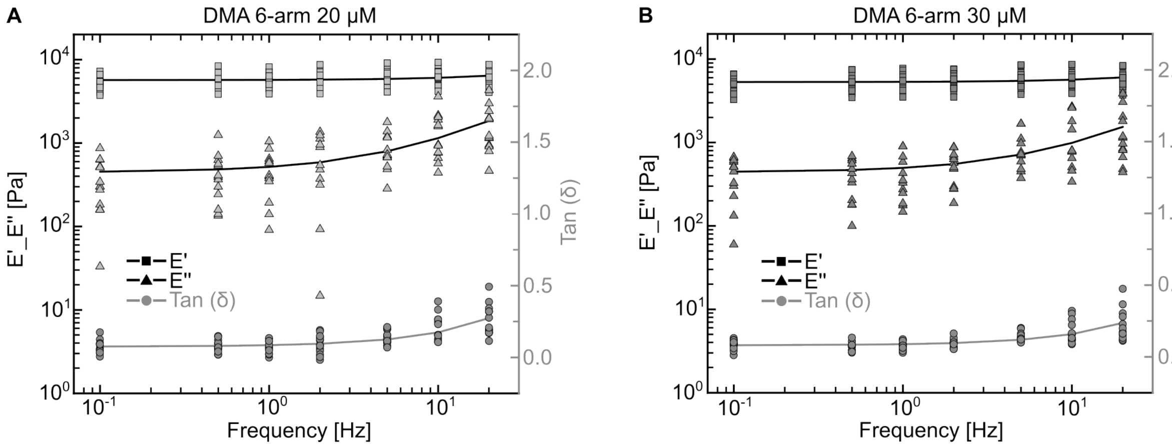

Figure S24: Further dynamic mechanical analysis (DMA) of 6-arm DNA-HMPs. A) DMA plot of 6-arm DNA-HMPs at 20 µM DNA concentration. The storage modulus E' (squares) and the loss modulus E" (triangles) are plotted on a logarithmic scale, tan (δ) (E"/E', filled circles) is plotted on a linear scale. Tan (δ) stays below 0.5 for all measured conditions with only a slight increase of E" at higher frequencies, denoting the 6-arm DNA-HMPs as behaving mostly elastic. B) DMA plot of 6-arm DNA-HMPs at 30 µM DNA concentration. The storage modulus E' (squares) and the loss modulus E" (triangles) are plotted on a logarithmic scale, tan (δ) (E"/E', filled circles) is plotted on a linear scale.

2.25 Figure S25: DNA-HMPs display different relaxation behaviors during microindentation

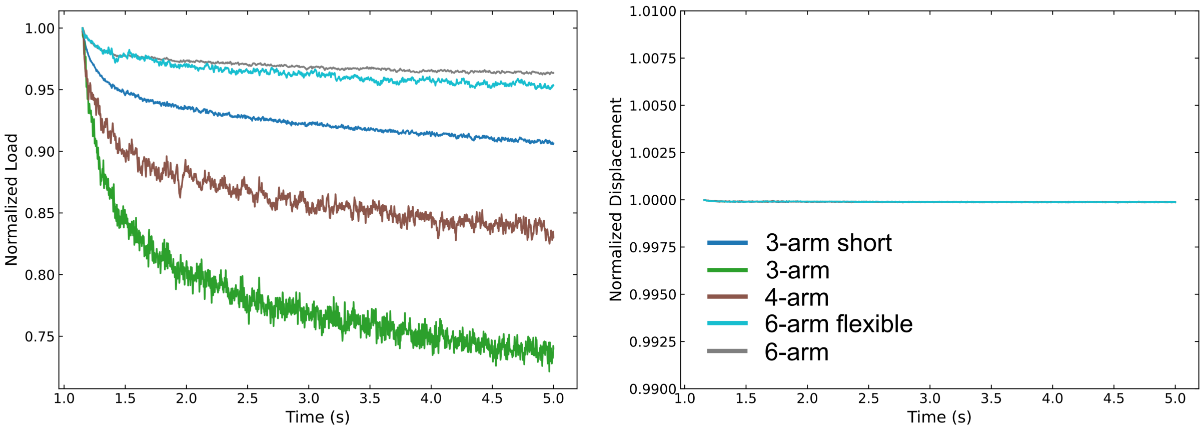

Figure S25: Relaxation behavior of DNA-HMPs across DNA nanostar designs. Directly after indentation prior to dynamic mechanical analysis, the DNA-HMPs are allowed to relax for 10 s. The normalized force detected by the cantilever during this relaxation period is plotted as a function of time for different DNA-HMP nanostars. 3-arm and 4-arm DNA-HMPs display a behavior consistent with a higher viscosity compared to the other designs (plot 1) with the 6-arm DNA-HMPs exhibiting the strongest elastic response. The normalized displacement of the cantilever is shown as a function of time for the same designs. Minimal initial displacement and variation are observed for all designs (plot 2).

2.26 Figure S26: Deformation of DNA-HMPs during RT-DC

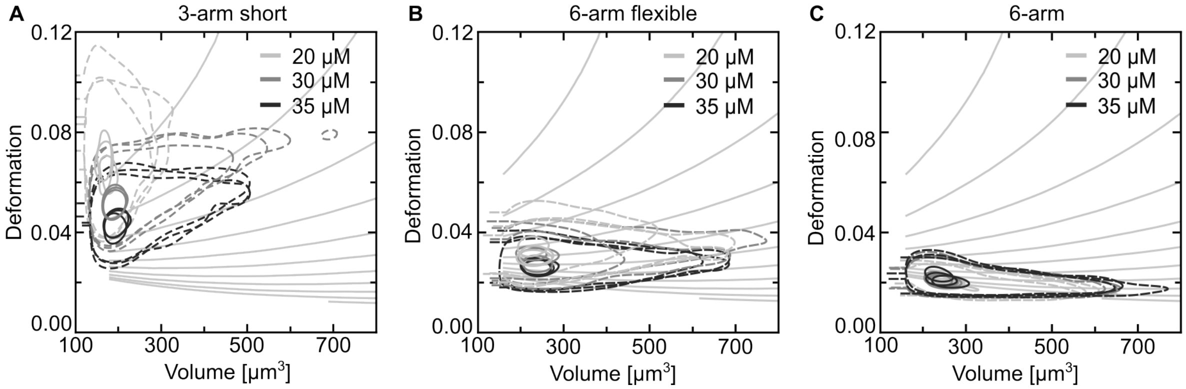

Figure S26: Full contour plots of DNA-HMP deformation during RT-DC showing all repeats. A) 3-arm short DNA-HMP deformation plotted against the volume for different DNA concentrations ($n_{20\,\mu M}$ = 45744, $n_{30\,\mu M}$ = 54771, $n_{35\,\mu M}$ = 52743). The data is presented using contour plots showing the 50th percentile (dashed line) and 95th percentile (solid line) of three independent measurements per condition. B) 6-arm flexible DNA-HMP deformation plotted against the volume for different DNA concentrations ($n_{20\,\mu M}$ = 23801, $n_{30\,\mu M}$ = 22632, $n_{35\,\mu M}$ = 18931). The data is presented using contour plots showing the 50th percentile (dashed line) and 95th percentile (solid line) of three independent measurements per condition. C) 6-arm DNA-HMP deformation plotted against the volume for different DNA concentrations ($n_{20\,\mu M}$ = 42430, $n_{30\,\mu M}$ = 42271, $n_{35\,\mu M}$ = 47844). The data is presented using contour plots showing the 50th percentile (dashed line) and 95th percentile (solid line) of three independent measurements per condition. Isoelasticity lines derived from numerical simulations are shown additionally, indicating stiffness changes where a steeper slope corresponds to softer particles.

2.27 Figure S27: Real-time deformability cytometry of 3-arm short DNA-HMPs

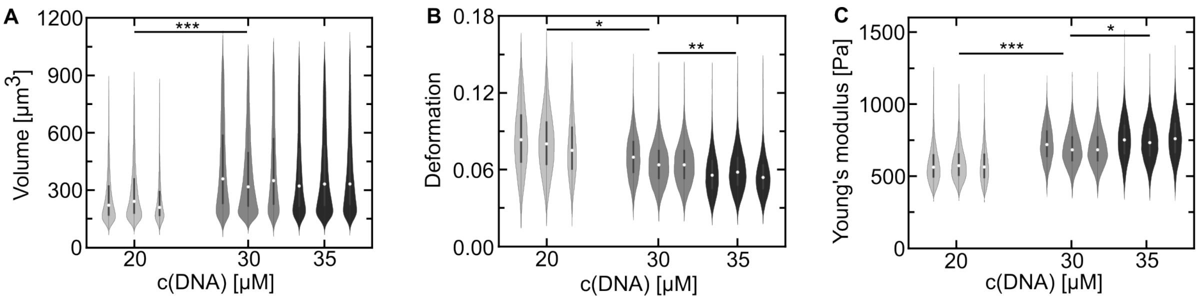

Figure S27: Analysis of 3-arm short DNA-HMPs using real-time deformability cytometry (RT-DC). A, B, C) Plots depicting changes in volume, deformation and Young's modulus of 3-arm short DNA-HMPs at 20 µM (n = 45744), 30 µM (n = 54771) and 35 µM (n = 52743) DNA concentration. The data is presented as violin plots showing the median value (white dot) of each measurement with boxplots encompassing the 25 - 75 % percentiles and a whisker length of 1.5 IQR. For each condition n = 3 independent experiments are displayed. DNA-HMP size increased significantly with an increase in DNA concentration from 20 µM, to 30 µM, while the size did not seem to change considerably when further increasing the DNA concentration to 35 µM (whole-population mean: $V_{20\,\mu M} = 273.7\,\mu m^3 \pm 2.1\,\mu m^3$, $V_{30\,\mu M} = 409.5\,\mu m^3 \pm 3\,\mu m^3$, $V_{35\,\mu M} = 397.1\,\mu m^3 \pm 2.9\,\mu m^3$). ***p-value: 0.0009. A significant decrease in deformation is shown for both 20 µM and 30 µM (also between 20 µM and 35 µM DNA-HMPs) as well as 30 µM and 35 µM DNA-HMPs (whole-population mean: $D_{20\,\mu M} = 0.081 \pm 0.0003$, $D_{30\,\mu M} = 0.069 \pm 0.0002$, $D_{35\,\mu M} = 0.058 \pm 0.0002$). *p-value: 0.015, ***p-value: 0.0005. Accordingly, the Young's modulus increased significantly between 20 µM and 30 µM DNA-HMPs as well as 30 µM and 35 µM DNA-HMPs (Whole population mean: $YM_{20\,\mu M} = 0.59\,kPa \pm 0.002\,kPa$, $YM_{30\,\mu M} = 0.71\,kPa \pm 0.002\,kPa$, $YM_{35\,\mu M} = 0.76\,kPa \pm 0.002\,kPa$). *p-value: 0.04. ***p-value: 0.0002. Statistical significance was analyzed using a linear mixed model (R-lme4) as integrated in Shape-Out (version 2.10.0) yielding ANOVA p-values. Error values correspond to the standard error of the mean.

2.28 Figure S28: Real-time deformability cytometry of 6-arm flexible DNA-HMPs

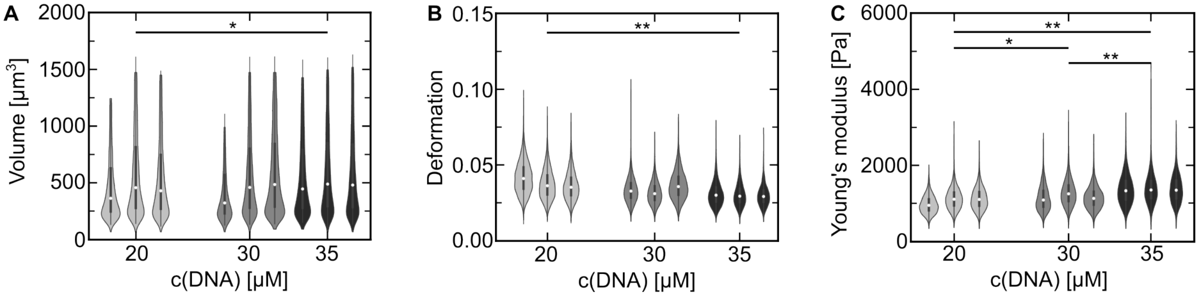

Figure S28: Analysis of 6-arm flexible DNA-HMPs using real-time deformability cytometry (RT-DC). A, B, C) Plots depicting changes in volume, deformation and Young's modulus of 6-arm flexible DNA-HMPs at 20 µM (n = 23801), 30 µM (n = 22632) and 35 µM (n = 18931) DNA concentration. The data is presented as violin plots showing the median value (white dot) of each measurement with boxplots encompassing the 25 - 75 % percentiles and a whisker length of 1.5 IQR. For each condition n = 3 independent experiments are displayed. DNA-HMP size increased significantly with an increase in DNA concentration from 20 µM to 35 µM (whole-population mean: $V_{20\,\mu M} = 540.4\,\mu m^3 \pm 6.3\,\mu m^3$, $V_{30\,\mu M} = 547.7\,\mu m^3 \pm 6.5\,\mu m^3$, $V_{35\,\mu M} = 591.9\,\mu m^3 \pm 6.8\,\mu m^3$). *p-value: 0.03. The deformation of the DNA-HMPs decreased significantly with an increase in DNA concentration from 20 µM to 35 µM (whole-population mean: $D_{20\,\mu M} = 0.039 \pm 0.0002$, $D_{30\,\mu M} = 0.035 \pm 0.0002$, $D_{35\,\mu M} = 0.031 \pm 0.0002$). **p-value: 0.0024. Likewise, the Young's modulus increased significantly from 20 µM to 35 µM between all concentrations (whole population mean: $YM_{20\,\mu M} = 1.09\,kPa \pm 0.006\,kPa$, $YM_{30\,\mu M} = 1.22\,kPa \pm 0.008\,kPa$, $YM_{35\,\mu M} = 1.41\,kPa \pm 0.009\,kPa$. *p-value: 0.034, **p-values: 0.007, 0.003. Statistical significance was analyzed using a linear mixed model (R-lme4) as integrated in Shape-Out (version 2.10.0) yielding ANOVA p-values. Error values correspond to the standard error of the mean.

2.29 Figure S29: Dynamic real-time deformability cytometry of 3-arm short DNA-HMPs

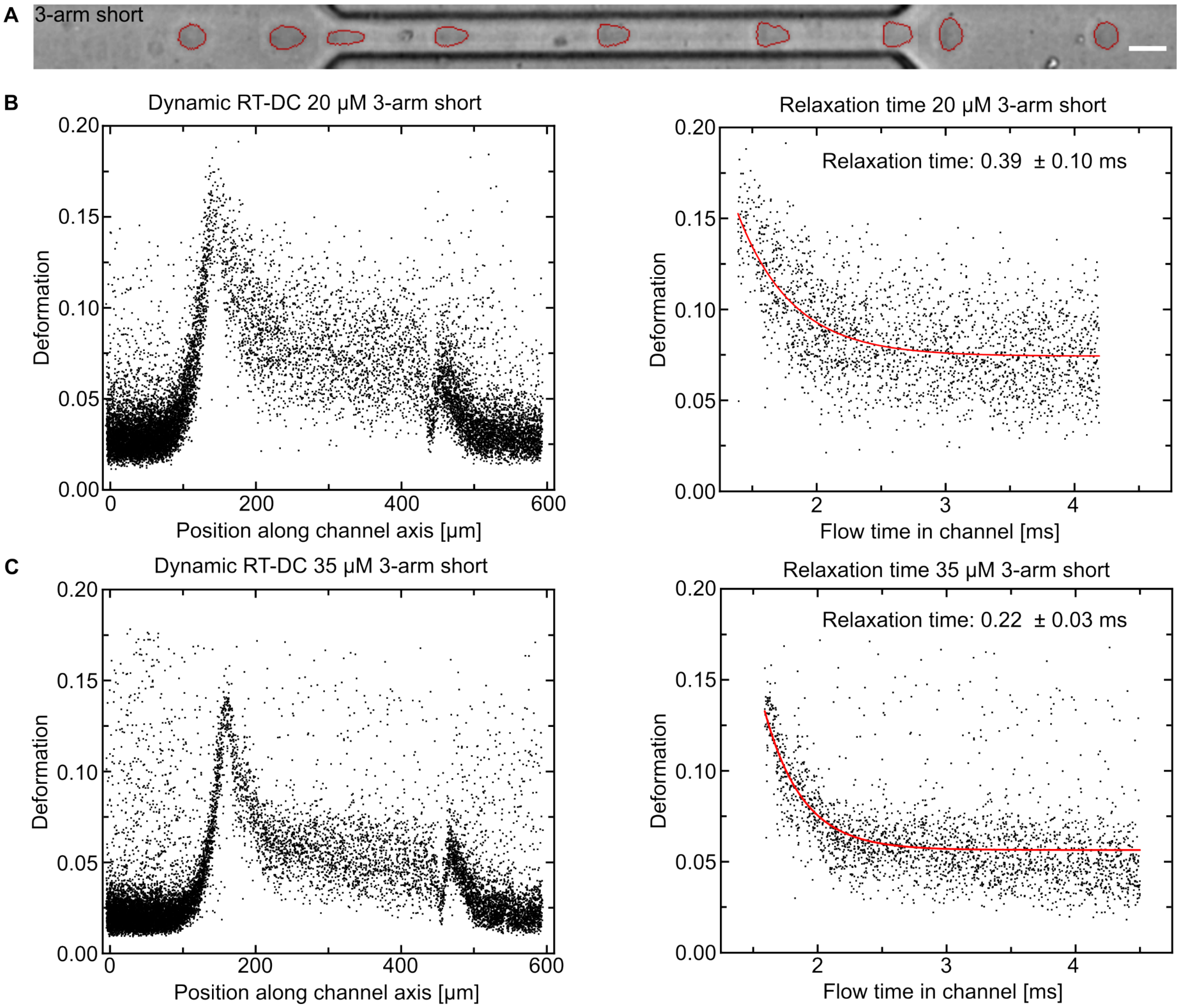

Figure S29: Dynamic real-time deformability cytometry (dRT-DC) of 3-arm short DNA-HMPs. A) Composite image of 3-arm short DNA-HMPs being deformed inside the flow channel during dRT-DC. The DNA-HMPs are spherical prior to channel-entry and initially deform strongly upon insertion into the channel. After the maximum deformation is reached, the particles relax and reach a steady-state deformation. After leaving the channel, the DNA-HMPs return to their spherical shape. Scale bar: 20 µm. B) Deformation of 20 µM DNA-HMPs plotted over the channel length during dRT-DC measurements. The DNA-HMPs initially deform strongly upon entering the channel. A steady-state of deformation is then reached within the channel following relaxation. The relaxation time of the DNA-HMPs was extracted from the decay of the exponential fit of the relaxation curve (DNA-HMP deformation over flow time). C) Deformation of 35 µM DNA-HMPs plotted over the channel length during dRT-DC measurements. The DNA-HMPs initially deform strongly upon entering the channel. A steady-state of deformation is then reached within the channel following relaxation. After the maximum deformation is reached, the particles relax and reach a steady-state of deformation. Deformation of 35 µM DNA-HMPs plotted over the flow time inside the RT-DC channel. The relaxation time of the DNA-HMPs was extracted from the decay of the exponential fit of the relaxation curve (DNA-HMP deformation over flow time). For calculation of the flow time see Experimental Section for more detail. Plotting and fitting of the data were conducted using OriginPro 2021 - Update 6 (Origin Lab Corporation).

2.30 Figure S30: Dynamic real-time deformability cytometry of 6-arm flexible DNA-HMPs

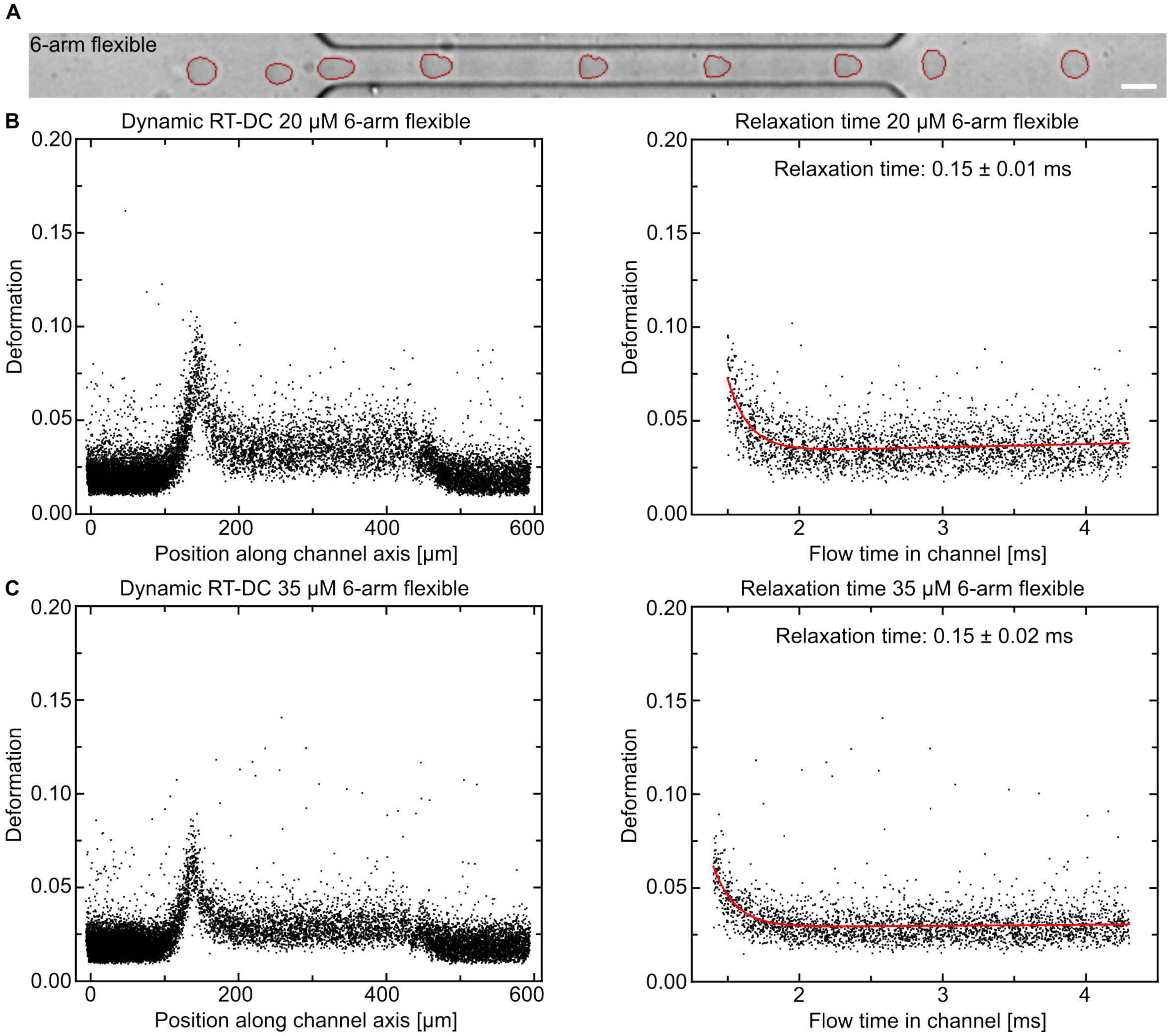

Figure S30: Analysis of 6-arm flexible DNA-HMPs using dynamic real-time deformability cytometry (dRT-DC). A) Composite image of 6-arm flexible DNA-HMPs being deformed inside the flow channel during dRT-DC. The DNA-HMPs are spherical prior to channel-entry and initially deform upon insertion into the channel, however much less than the 3-arm short DNA-HMPs. After the maximum deformation is reached, the particles relax and reach a steady-state deformation. After leaving the channel, the DNA-HMPs return to their spherical shape. Scale bar: 20 µm. B) Deformation of 6-arm flexible DNA-HMPs at 20 µM DNA concentration plotted over the channel length during dRT-DC measurements. The relaxation time of the DNA-HMPs was extracted from the decay of the exponential fit of the relaxation curve (DNA-HMP deformation over flow time). C) Deformation of 6-arm flexible DNA-HMPs at 35 µM DNA concentration plotted over the channel length during dRT-DC measurements. The relaxation time of the DNA-HMPs was extracted from the decay of the exponential fit of the relaxation curve (DNA-HMP deformation over flow time). For calculation of the flow time see Experimental Section. Plotting and fitting of the data were conducted using OriginPro 2021 - Update 6 (Origin Lab Corporation).

2.31 Figure S31: Real-time deformability cytometry of 6-arm DNA-HMPs

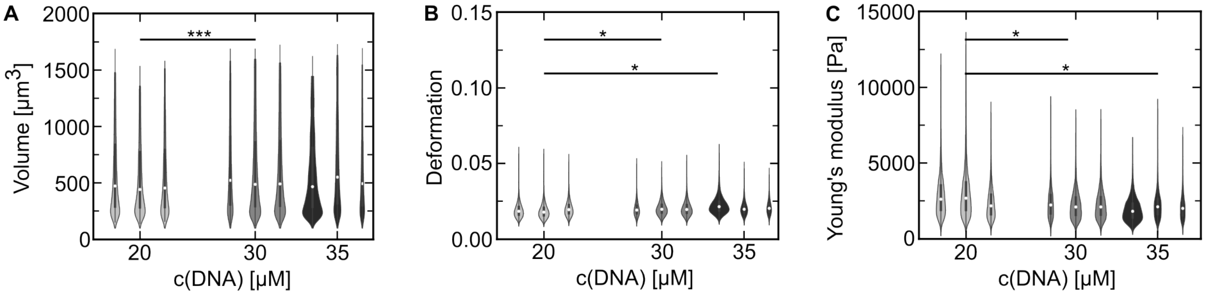

Figure S31: Analysis of 6-arm DNA-HMPs using real-time deformability cytometry (RT-DC). A, B, C) Plots depicting changes in volume, deformation and Young's modulus of 6-arm DNA-HMPs at 20 µM (n = 42430), 30 µM (n = 42271) and 35 µM (n = 47844) DNA concentration. The data is presented as violin plots showing the median value (white dot) of each measurement with boxplots encompassing the 25 - 75 % percentiles and a whisker length of 1.5 IQR. For each condition n = 3 independent experiments are displayed. DNA-HMP size increased significantly with an increase in DNA concentration from 20 µM to 30 µM (Whole-population mean volume: $V_{20\,\mu M} = 625.7\,\mu m^3 \pm 6.8\,\mu m^3$, $V_{30\,\mu M} = 629.1\,\mu m^3 \pm 7\,\mu m^3$, $V_{35\,\mu M} = 590.2\,\mu m^3 \pm 6.7\,\mu m^3$). ***p-value: 0.0002. The deformation of the DNA-HMPs increased significantly with an increase in DNA concentration from 20 µM to 30 µM and 35 µM (Whole-population mean deformation: $D_{20\,\mu M} = 0.019 \pm 0.0001$, $D_{30\,\mu M} = 0.021 \pm 0.0001$, $D_{35\,\mu M} = 0.022 \pm 0.0001$). *p-values: 0.039, 0.032. Likewise the Young's modulus decreased significantly between the same concentrations (Whole population mean: $YM_{20\,\mu M} = 2.84\,kPa \pm 0.03\,kPa$, $YM_{30\,\mu M} = 2.36\,kPa \pm 0.02\,kPa$, $YM_{35\,\mu M} = 2.15\,kPa \pm 0.02\,kPa$). *p-values: 0.043, 0.019. Statistical significance was analyzed using a linear mixed model (R-lme4) as integrated in Shape-Out (version 2.10.0) yielding ANOVA p-values. Error values correspond to the standard error of the mean.

2.32 Figure S32: Real-time deformability cytometry of 6-arm DNA-HMPs at 0.4 µL/s flow rate

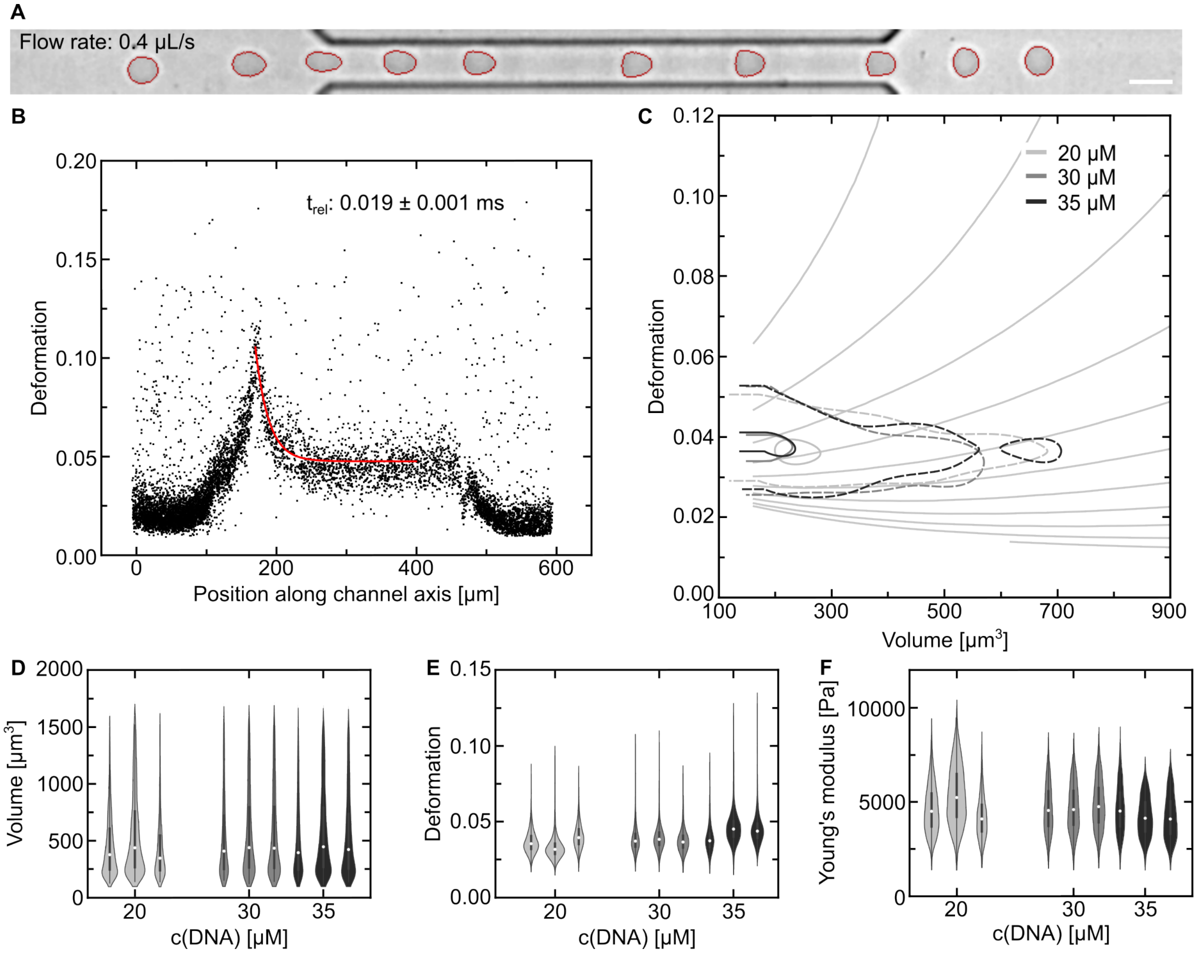

Figure S32: Analysis of the material properties of 6-arm DNA-HMPs using real-time deformability cytometry (RT-DC) at 0.4 µL/s flow rate. A) Bright field images of 6-arm DNA-HMPs inside the flow channel during dynamic RT-DC (dRT-DC). The red line indicates the detected contours of the HMPs. Scale bar: 20 µm. B) Deformation scatter plot of 35 µM 6-arm HMPs plotted over the channel length during dRT-DC. Each point corresponds to an individual DNA-HMP (n = 8000). The relaxation time t_{rel} of the DNA-HMPs was extracted from the exponential fit of the relaxation scatter plot (red curve). For calculation of the flow time see Experimental Section. C) Exemplary contour plot showing 6-arm DNA-HMP steady-state deformation over the particle volume for different DNA nanostar concentrations. The data is presented using contour plots showing the 50th percentile (dashed line) and 95th percentile (solid line). Isoelasticity lines derived from numerical simulations are shown additionally, indicating stiffness changes where a steeper slope corresponds to softer particles. Only one repeat per condition is depicted to improve readability. D, E, F) Plots depicting changes in volume, deformation and Young's modulus of 6-arm DNA-HMPs at 20 µM (n = 11306), 30 µM (n = 8247) and 35 µM (n = 9617) DNA concentration. The data is presented as violin plots showing the median value (white dot) of each measurement with boxplots encompassing the 25 - 75 % percentiles and a whisker length of 1.5 IQR. For each condition n = 3 independent experiments are displayed. Whole-population mean volume: $V_{20\,\mu M} = 487.9\,\mu m^3 \pm 5.1\,\mu m^3$, $V_{30\,\mu M} = 554.5\,\mu m^3 \pm 7\,\mu m^3$, $V_{35\,\mu M} = 541.1\,\mu m^3 \pm 6.3\,\mu m^3$. Whole-population mean deformation: $D_{20\,\mu M} = 0.0374 \pm 0.0001$, $D_{30\,\mu M} = 0.0391 \pm 0.0002$, $D_{35\,\mu M} = 0.0440 \pm 0.0002$. Whole population mean Young's modulus: $YM_{20\,\mu M} = 4.76\,kPa \pm 0.02\,kPa$, $YM_{30\,\mu M} = 4.75\,kPa \pm 0.02\,kPa$, $YM_{35\,\mu M} = 4.37\,kPa \pm 0.02\,kPa$. No significant differences were detected for the presented data. Statistical significance was analyzed using a linear mixed model (R-lme4) as integrated in Shape-Out (version 2.10.0) yielding ANOVA p-values. Errors correspond to the standard error of the mean.

2.33 Figure S33: Polyacrylamide gel electrophoresis of modified and unmodified elongated linker, 6-arm linker and flexible 6-arm linker

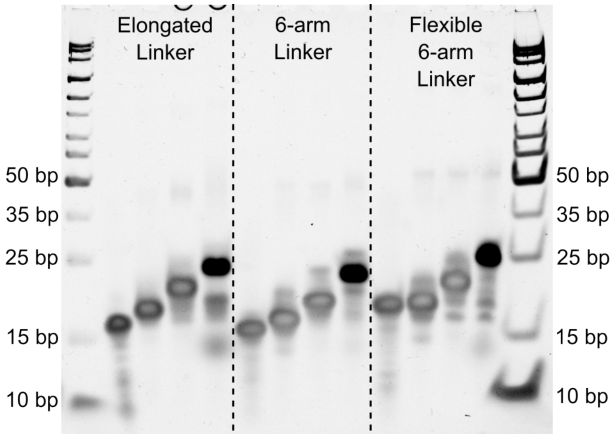

Figure S33: Polyacrylamide gel electrophoresis (PAGE) of the elongated linker, 6-arm-linker and flexible 6-arm-linker with and without modifications. In each set of four lanes the respective DNA linkers are depicted as follows. 1: The unmodified linker, 2: DBCO-modified linker, 3: RGD-modified linker and 4: 5-FAM-modified linker. The marked size increase of the modified linkers above the non-modified DNA indicates the coupling of the respective modifications to the intact DNA linkers. Note that due to the fluorescent nature of the 5-FAM-tag the band appears much darker.

2.34 Figure S34: Incorporation of 5-FAM-modified DNA linkers into 3-arm DNA-HMPs

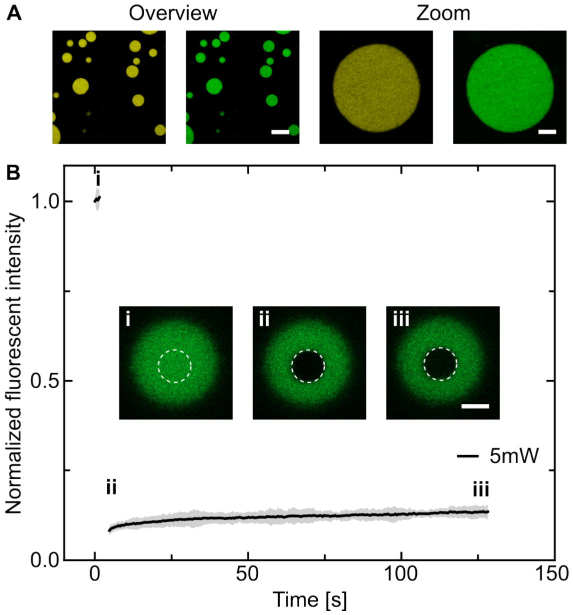

Figure S34: Uptake and distribution of 5-FAM-tagged DNA linkers into DNA-HMPs. A) Confocal fluorescence images (λ_{ex} = 561 nm, Cy3-labeled DNA, yellow, λ_{ex} = 488 nm, 5-FAM-labeled DNA linker, green) of 3-arm DNA-HMPs after overnight incubation with 5-FAM-modified DNA linkers. Overview scale bar: 50 µm; Zoom scale bar: 10 µm. The 5-FAM signal is distributed homogeneously throughout the DNA-HMP. B) FRAP on the incorporated 5-FAM signal using 5 mW of laser power at 10 µm ROI size. Inlays show confocal fluorescence microscopy images of DNA-HMPs before (*i*) and after (*ii*, *iii*) bleaching. Scale bar: 10 µm. FRAP data is shown as combined mean ± error propagated standard deviation of triplicate measurements measuring three individual DNA-HMPs per replicate. No recovery is observed, indicating successful covalent linkage.

2.35 Figure S35: Incorporation of 5-FAM-modified DNA linkers into 6-arm flexible and 6-arm DNA-HMPs

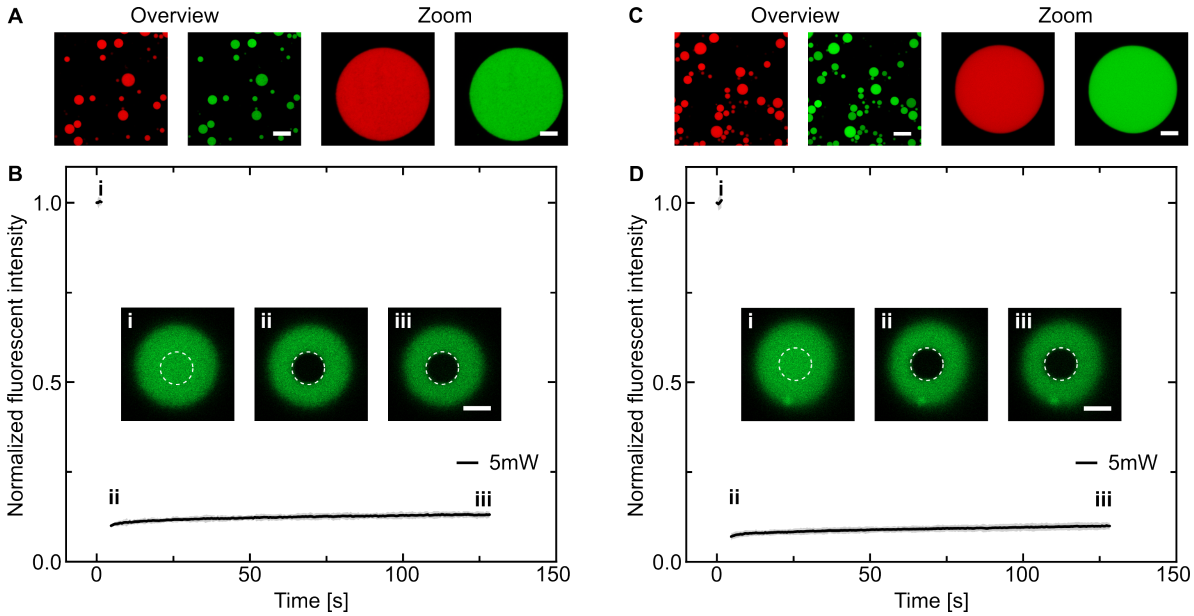

Figure S35: Uptake and distribution of 5-FAM-tagged DNA linkers into DNA-HMPs. A) Confocal fluorescence image ($\lambda_{ex} = 640$ nm, ATTO-647N-labeled DNA, red, and 5-FAM-labeled DNA linker, green) of 6-arm flexible DNA-HMPs after overnight incubation with 5-FAM-modified DNA linkers. Overview scale bar: 50 µm; Zoom scale bar: 10 µm. The 5-FAM signal is distributed homogeneously throughout the DNA-HMP. B) FRAP on the incorporated 5-FAM signal using 5 mW of laser power at 10 µm ROI size. Inlays show confocal fluorescence microscopy images of DNA-HMPs before (*i*) and after (*ii*, *iii*) bleaching. Scale bar: 10 µm. C) Confocal fluorescence images (ATTO-647N-labeled DNA, red, and 5-FAM-labeled DNA linker, green) of 6-arm DNA-HMPs after overnight incubation with 5-FAM-modified DNA linkers. Overview scale bar: 50 µm; Zoom scale bar: 10 µm. The 5-FAM signal is distributed homogeneously throughout the DNA-HMP. D) FRAP on the incorporated 5-FAM signal using 5 mW of laser power at 10 µm ROI size. Inlays show confocal fluorescence microscopy images of DNA-HMPs before (*i*) and after (*ii*, *iii*) bleaching. Scale bar: 10 µm. FRAP data is shown as combined mean ± error propagated standard deviation of triplicate measurements measuring three individual DNA-HMPs per replicate. No recovery is observed, indicating successful covalent linkage.

2.36 Figure S36: Analysis of 5-FAM-modified DNA linker uptake into DNA-HMPs

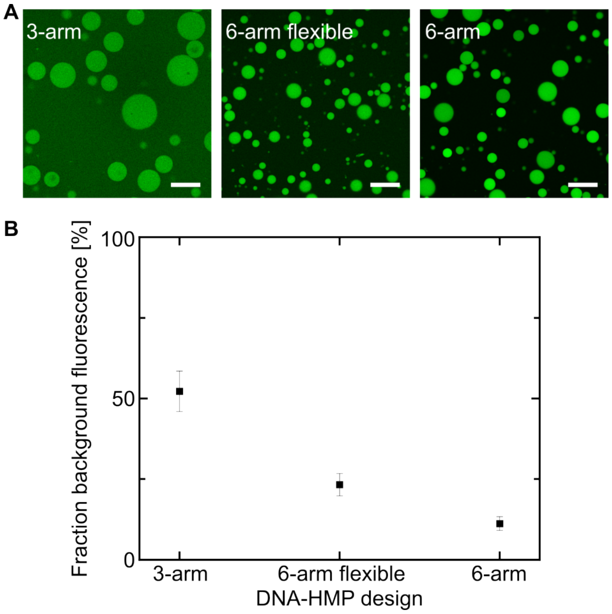

Figure S36: Uptake and distribution of 5-FAM-tagged DNA linkers into DNA-HMPs. A) Confocal fluorescence overview images ($\lambda_{ex} = 488\,\text{nm}$, 5-FAM-labeled DNA linker, green) of 3-arm, 6-arm flexible and 6-arm DNA-HMPs after overnight incubation with 5-FAM-modified linker prior to washing. B) Plot depicting the fraction of background fluorescence measured as background intensity over DNA-HMP intensity for 3-arm, 6-arm flexible and 6-arm DNA-HMPs after overnight incubation with 5-FAM-modified linker prior to washing. While the modified 3-arm linker shows uptake of roughly 50%, the 6-arm flexible and 6-arm linker show uptake of 75% and 90%. Data is shown as combined mean ± error propagated standard deviation of triplicate measurements of each design.

2.37 Figure S37: Analysis of DNA-HMP stability under cell culture conditions

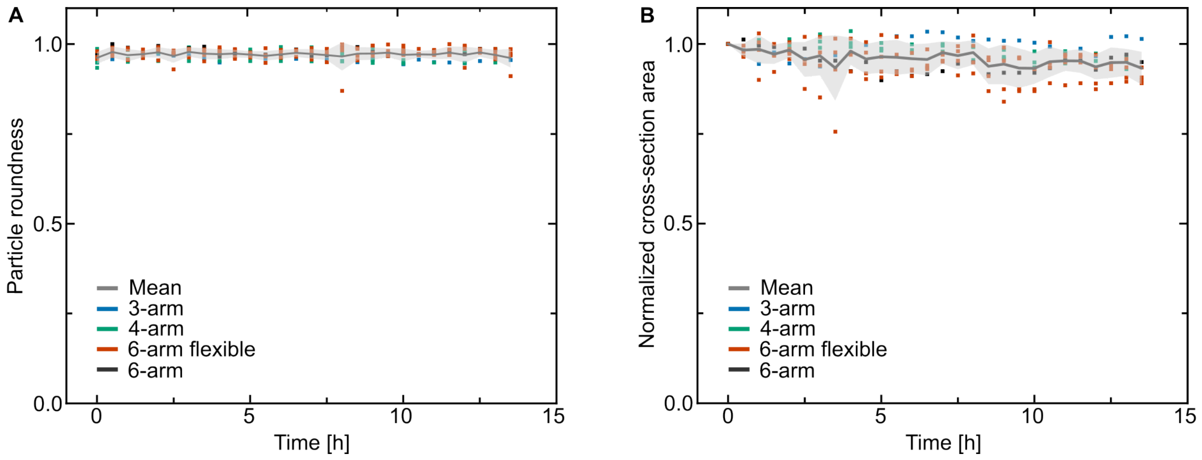

Figure S37: DNA-HMP stability in fibroblast culture. A) Individual (colored squares) and population mean ± standard deviation (gray line + shaded area) particle roundness of DNA-HMPs found outside of fibroblast spheroids in the culture medium plotted over time. B) Individual (colored squares) and population mean ± standard deviation (gray line + shaded area) normalized cross-section area of DNA-HMPs found outside of fibroblast spheroids in the culture medium plotted over time. For each DNA-HMP, area was normalized to the first frame. A total of 9 DNA-HMPs from 8 separate experimental field-of-views were analyzed.

2.38 Figure S38: DNA-HMP particle elongation and measured traction forces

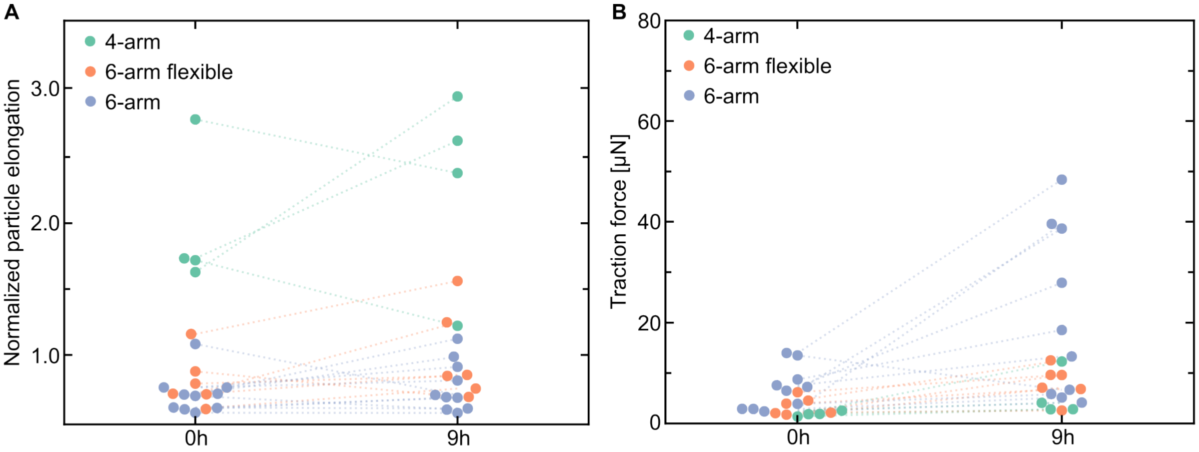

Figure S38: DNA-HMP particle elongation and measured traction forces inside fibroblast spheroids. A) Graph showing the normalized particle elongation of DNA-HMPs within fibroblast spheroid culture after 0 h and 9 h of culture. B) Graph showing traction forces applied by the fibroblasts on the DNA-HMPs within fibroblast spheroid culture after 0 h and 9 h of culture. Data are plotted as single data points, connecting lines denote the same DNA-HMPs at different time-points.

WILEY-VCH

2.39 Figure S39: DNA-HMP deformation as a function of radial position in fibroblast spheroids

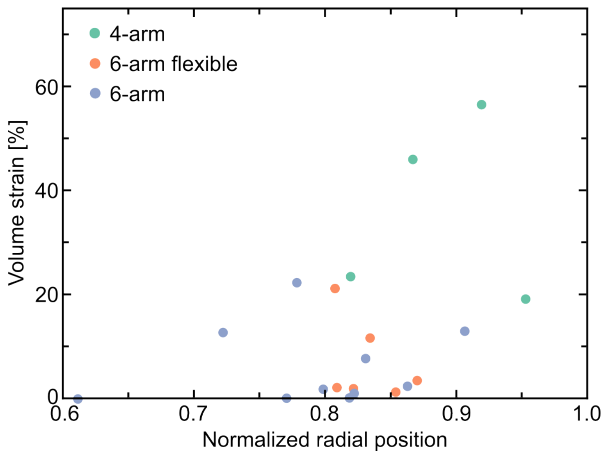

Figure S39: DNA-HMP deformation within fibroblast spheroids as a function of location. Graph depicts DNA-HMP deformation measured as volume strain within fibroblast spheroids after 9 h of culture showing larger deformations for softer DNA-HMPs and towards spheroid periphery. Data are plotted as single data points.

2.40 Figure S40: Extraction of drag force amplitude via force time series

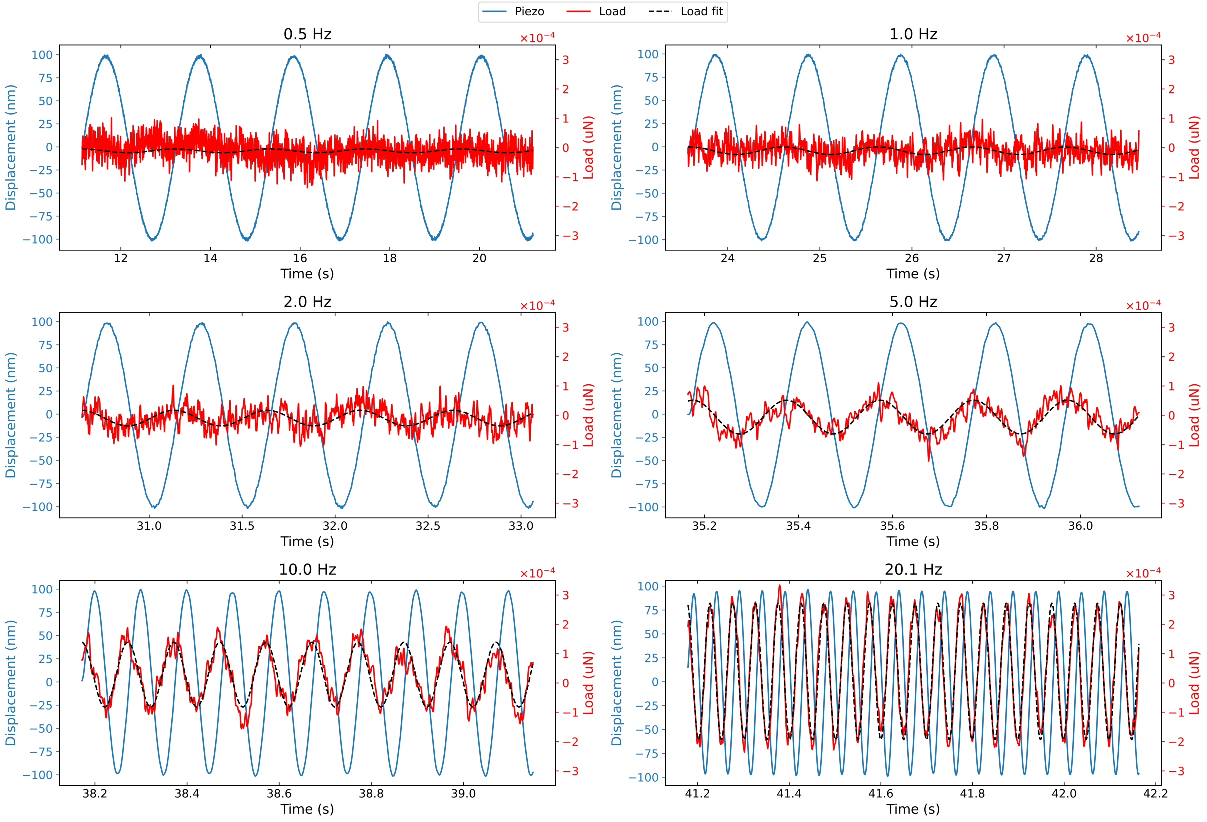

Figure S40: Drag force extraction from force time series. Force time series (red) measured by the cantilever, which is displaced in liquid by a piezoelectric actuator operating at varying frequencies. The displacement signal (blue) represents the actuator's measured position. Each subplot displays the dynamic response at different driving frequencies: 0.5 Hz, 1.0 Hz, 2.0 Hz, 5.0 Hz, 10.0 Hz, and 20.1 Hz. The black dashed line shows the sine-fitted load curve for each frequency, highlighting the increase in amplitude with frequency and the expected 90-degree phase delay, characteristic of a drag force.

2.41 Figure S41: Correction of the loss modulus by the viscous drag contribution

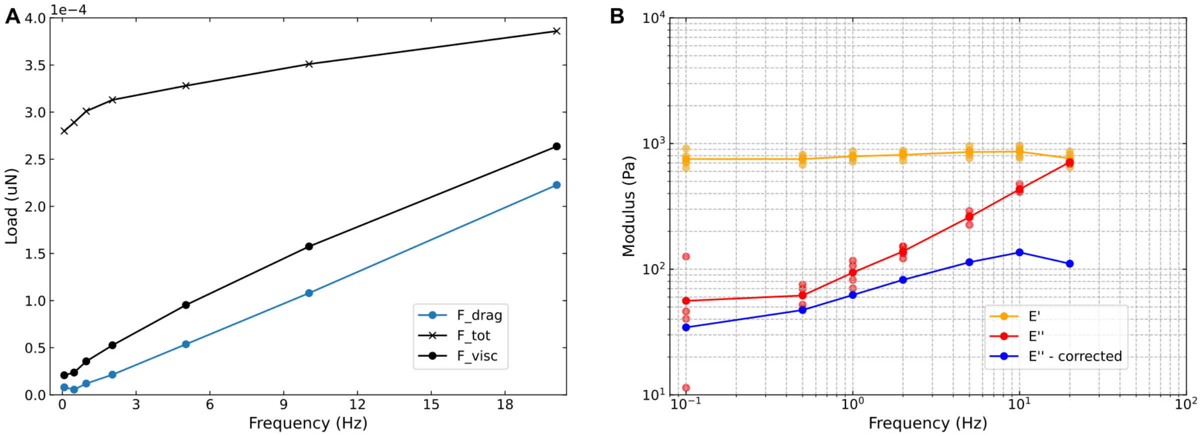

Figure S41: Correction of loss modulus E” by viscous drag. A) The load on the cantilever as a function of frequency is shown for three forces: The drag force, F_{drag} (blue dots), extracted as shown in Figure S40; the total force, F_{tot} (crosses), provided by the manufacturer’s analysis software; and the viscous force, F_{visc}, calculated using F_{tot} and the loss tangent, $tan(\delta)$. The data shown represents a typical measurement for 3-arm short DNA-HMPs, where F_{drag} accounts for the majority of F_{visc}. B) The storage modulus, E’, and loss modulus E” are plotted against frequency for n = 5 measurements of 3-arm short DNA-HMPs. After accounting for the contribution of F_{drag} to F_{visc}, a correction is made to E” (see Supplementary Note 1).

3 Supporting Videos

3.1 Video S1: Droplet-templated formation of 3-arm short DNA-HMP over time

Overlay of confocal fluorescence microscopy (λ_{ex} = 561 nm, Cy3-labeled DNA, yellow) and brightfield time-lapse of a 3-arm short DNA-HMP forming inside a water-in-oil droplet over the course of two hours. The DNA condenses across the whole water-in-oil droplet to form a single DNA-HMP. Scale bar: 10 µm.

3.2 Video S2: Fluorescence recovery after photobleaching of released DNA-HMP

Confocal fluorescence microscopy (λ_{ex} = 561 nm, Cy3-labeled DNA, yellow) time-lapse of a released 3-arm short DNA-HMP during a fluorescence recovery after photobleaching (FRAP) experiment. The bleached area does not recover after bleaching, confirming the formation of a stable gel-phased structure. Scale bar: 10 µm.

3.3 Video S3: Integration of 3-arm DNA-HMPs into 3D fibroblast spheroids

Overlay of a confocal fluorescence microscopy (λ_{ex} = 561 nm, td-tomato-labeled mouse liver fibroblasts, yellow, λ_{ex} = 405 nm, ATTO-390-labeled DNA, cyan) z-stack of 3-arm DNA-HMPs embedded into a fibroblast spheroid after 48 h of hanging drop co-culture. Scale bar: 100 µm.

3.4 Video S4: Integration of 4-arm DNA-HMPs into 3D fibroblast spheroids

Overlay of a confocal fluorescence microscopy (λ_{ex} = 561 nm, td-tomato-labeled mouse liver fibroblasts, yellow, λ_{ex} = 488 nm, ATTO-488-labeled DNA, green) z-stack of 4-arm DNA-HMPs embedded into a fibroblast spheroid after 48 h of hanging drop co-culture. Scale bar: 100 µm.

3.5 Video S5: Integration of 6-arm flexible DNA-HMPs into 3D fibroblast spheroids

Overlay of a confocal fluorescence microscopy (λ_{ex} = 561 nm, td-tomato-labeled mouse liver fibroblasts, yellow, λ_{ex} = 640 nm, ATTO-647-labeled DNA, red) z-stack of 6-arm flexible DNA-HMPs embedded into a fibroblast spheroid after 48 h of hanging drop co-culture. In some cases the fibroblasts invaded into 6-arm flexible DNA-HMPs, effectively breaking them open. We attribute this to the combination of high stiffness and more porous nature of this design. Scale bar: 100 µm.

3.6 Video S6: Integration of 6-arm DNA-HMPs into 3D fibroblast spheroids

Overlay of a confocal fluorescence microscopy (λ_{ex} = 561 nm, td-tomato-labeled mouse liver fibroblasts, yellow, λ_{ex} = 640 nm, ATTO-647-labeled DNA, red) z-stack of 6-arm DNA-HMPs embedded into a fibroblast spheroid after 48 h of hanging drop co-culture. Scale bar: 100 µm.

3.7 Video S7: Deformation of a 3-arm DNA-HMP in a 3D fibroblast spheroid over time

Overlay of a confocal fluorescence microscopy (λ_{ex} = 561 nm, td-tomato-labeled mouse liver fibroblasts, yellow, λ_{ex} = 405 nm, ATTO-390-labeled DNA, cyan) time-lapse of a 3-arm DNA-HMP being deformed in a fibroblast spheroid by the surrounding cells over the course of 13.5 h. Scale bar: 20 µm.

3.8 Video S8: Deformation of a 4-arm DNA-HMP in a 3D fibroblast spheroid over time

Overlay of a confocal fluorescence microscopy ($\lambda_{ex} = 561$ nm, td-tomato-labeled mouse liver fibroblasts, yellow, $\lambda_{ex} = 488$ nm, ATTO-488-labeled DNA, green) time-lapse of a 4-arm DNA-HMP being deformed in a fibroblast spheroid by the surrounding cells over the course of 14 h. Scale bar: 20 µm.

3.9 Video S9: Deformation of a 6-arm flexible DNA-HMP in a 3D fibroblast spheroid over time

Overlay of confocal fluorescence microscopy ($\lambda_{ex} = 561$ nm, td-tomato-labeled mouse liver fibroblasts, yellow, $\lambda_{ex} = 640$ nm, ATTO-647-labeled DNA, red) time-lapse of a 6-arm flexible DNA-HMP being deformed in a fibroblast spheroid by the surrounding cells over the course of 13.5 h. Scale bar: 20 µm.

3.10 Video S10: Deformation of a 6-arm DNA-HMP in a 3D fibroblast spheroid over time

Overlay of a confocal fluorescence microscopy ($\lambda_{ex} = 561$ nm, td-tomato-labeled mouse liver fibroblasts, yellow, $\lambda_{ex} = 640$ nm, ATTO-647-labeled DNA, red) time-lapse of a 6-arm DNA-HMP being deformed in a fibroblast spheroid by the surrounding cells over the course of 13.5 h. Scale bar: 20 µm.

4 Supplementary Note 1: Correction for drag force

When a cantilever oscillates in a viscous medium, it experiences a force even in the absence of physical contact with a surface. This force is due to hydrodynamic drag, which depends on the viscosity and density of the medium, the size of the cantilever, and the frequency of oscillation. Thus, it needs to be determined for any given set of experimental parameters. If not accounted for, this viscous drag can be mistakenly attributed to the properties of the material being tested during dynamic mechanical measurements. To account for the viscous drag in our measurements, we applied the following steps:

Measurement of the drag force: We measured the viscous drag force during DMA as described in the experimental section *Measurement of hydrodynamic drag forces during microindentation*. The force amplitude at each frequency of the freely oscillating cantilever was then determined by fitting a sine wave to the data (Figure S40). Based on five individual measurements we calculated an average drag force F_{drag}.

Calculating the viscous force: Using the analysis data extracted from the original DMA measurements from the analysis software DataViewer (V2.5.0, Optics11Life), the loss tangent $tan(\delta)$ is calculated as E''/E'. Together with the detected load amplitude A, this is taken to calculate $F_{\text{visc}} = A * sin(\delta)$ (Figure S41).

Calculating geometrical conversion factor: The analysis software applies a geometrical conversion factor, to convert the force to a modulus, which we back-calculate as: $C = E''/F_{\text{visc}}$.

Correcting the loss modulus: Finally, we correct the loss modulus E'' by subtracting the drag force contribution as measured earlier via $E''_{\text{corrected}} = E'' - C * F_{\text{drag}}$, which can also be written as $E''_{\text{corrected}} = E''(1 - F_{\text{drag}}/F_{\text{visc}})$.

5 Supplementary Note 2: Estimation of c[RGD] ligand density on the surface of DNA-HMPs

We assume an average DNA-HMP diameter of 25 µm (Figure 1C), a DNA-HMP volume fraction of 20% after centrifugation (Experimental Section) and 100% uptake of the RGD-modified linkers. Hence, 10 µM of added modified linker thus yield an effective concentration of 50 µM RGD homogeneously distributed across the whole volume of the DNA-HMPs (Figures S34/S35).

Having calculated ligand concentration per HMP, we next assess the fibroblast-accessible HMP volumes, assuming 0.5 - 2 µm invadopodia length into our porous hydrogels [1, 2].

We can thus calculate the volumes of the resulting spherical shells as follows:

$$V_s = \frac{4}{3} \cdot \pi \cdot (r_1^3 - r_2^3) \tag{1}$$

yielding 943 µm^3 and 3332 µm^3 shell volumes for the different invading depths of 0.5 - 2 µm.

From this, we then calculate the number of RGDs (N_R) in each of these volumes by assuming a total concentration of 50 µM in the shell from above:

$$N_R = (V \cdot 50\mu M) \cdot N_A \tag{2}$$

to yield roughly 28 Mio. RGD ligands for V_0 and 100 Mio. for V_2.

To understand what this particle number means in terms of RGD density on the surface, we then project it onto the surface area of the whole sphere using the equation of the surface area of a sphere:

$$A = 4 \cdot \pi \cdot r^2 \tag{3}$$

given that the cells would enter the penetration volumes and thus be able to attach to the ligands in it. For the given diameter of 25 µm, we get a surface area of 1963 µm^2. Using the number of RGDs calculated above, we thus calculate RGD densities of $\approx$ 14000 RGD/µm^2 and $\approx$ 50000 RGD/µm^2 for invading depths of 0.5 µm and 2 µm, respectively.

Finally, assuming a hydrodynamic radius of the RGD-modified DNA oligo of roughly 2.2 nm [3] and the length of the oligo of 25 nt, we estimate every strand to occupy an area of 4.84 nm^2. Given the particle number calculated earlier, we thus get a total density of $\approx$ 68 000 nm^2 and $\approx$ 240 000 nm^2 and thus an effective occupancy of 7 - 24% of the RGD on the DNA-HMP surface for the different invading depths. Assuming even spacing, we can thus estimate ligand density on the DNA-HMP surface to a spacing of 4 - 14 nm, should 100% of the RGD-modified linker be taken up.

These values are well comparable both with the size of the nanostars which are on the order of 20 nm in diameter, as well as with values of RGD-packing densities from literature, in which a 10 nm spacing is preferred given the size of integrin at 8 - 12 nm [4] and good cell attachment being found until an RGD

spacing distance of 70 nm [5, 6].

While, of course, 100% linker conversion and eventual uptake are unlikely, we quantified the fraction of unbound 5-FAM signal over the fluorescence signal of 5-FAM inside of DNA-HMPs after overnight incubation and no washing of the DNA-HMPs (Figure S36) in order to estimate the fraction of actually uptaken compounds. While we see roughly 50% uptake for the elongated linker, we can find uptake of 75 - 90% for the 6-arm linkers, most likely due to better linking conversion of the 5-FAM construct. Thus, we conclude that we can achieve at least 50% of the possible particle density given above per reaction. The lower estimate for the ligand density of RGD on the DNA-HMP surface thus comes to a ligand spacing of roughly 8 - 28 nm and thus still within the relevant range for cell attachment.

References

[1] A. Mogilner and B. Rubinstein, “The physics of filopodial protrusion,” *Biophysical journal*, vol. 89, no. 2, pp. 782–795, 2005.

[2] A. Parekh and A. M. Weaver, “Regulation of invadopodia by mechanical signaling,” *Experimental cell research*, vol. 343, no. 1, pp. 89–95, 2016.

[3] M. Reichl, M. Herzog, F. Greiss, M. Wolff, and D. Braun, “Understanding the similarity in thermophoresis between single- and double-stranded dna or rna,” *Physical review. E, Statistical, nonlinear, and soft matter physics*, vol. 91, no. 6, p. 062709, 2015.

[4] J.-P. Xiong, T. Stehle, R. Zhang, A. Joachimiak, M. Frech, S. L. Goodman, and M. A. Arnaout, “Crystal structure of the extracellular segment of integrin alpha vbeta3 in complex with an arg-gly-asp ligand,” *Science (New York, N.Y.)*, vol. 296, no. 5565, pp. 151–155, 2002.

[5] M. Arnold, E. A. Cavalcanti-Adam, R. Glass, J. Blümmel, W. Eck, M. Kantlehner, H. Kessler, and J. P. Spatz, “Activation of integrin function by nanopatterned adhesive interfaces,” *Chemphyschem : a European journal of chemical physics and physical chemistry*, vol. 5, no. 3, pp. 383–388, 2004.

[6] X. Wang, C. Yan, K. Ye, Y. He, Z. Li, and J. Ding, “Effect of rgd nanospacing on differentiation of stem cells,” *Biomaterials*, vol. 34, no. 12, pp. 2865–2874, 2013.
